# Strontium isotope evidence for a trade network between southeastern Arabia and India during Antiquity

**DOI:** 10.1038/s41598-020-79675-3

**Published:** 2021-01-11

**Authors:** Saskia E. Ryan, Vladimir Dabrowski, Arnaud Dapoigny, Caroline Gauthier, Eric Douville, Margareta Tengberg, Céline Kerfant, Michel Mouton, Xavier Desormeau, Antoine Zazzo, Charlène Bouchaud

**Affiliations:** 1 Archéozoologie, Archéobotanique: Sociétés, Pratiques et Environnements (AASPE, UMR 7209), Muséum National d’Histoire Naturelle, CNRS, CP56, 55 rue Buffon, 75005 Paris, France; 2 Laboratoire des Sciences du Climat et de l’Environnement, LSCE/IPSL, UMR CEA-CNRS-UVSQ, Université Paris-Saclay, 91191 Gif-sur-Yvette, France; 3 Institut Català de Paleoecologia Humana i Evolució Social (IPHES-CERCA), Zona Educacional 4, Campus Sescelades URV (Edifici W3), 43007 Tarragona, Spain; 4 Institut Français du Proche-Orient, B.P. 11-1424, Beyrut, Lebanon

**Keywords:** Geochemistry, Biogeochemistry

## Abstract

Cotton (*Gossypium* sp.), a plant of tropical and sub-tropical origin, appeared at several sites on the Arabian Peninsula at the end of the 1st mill. BCE-beginning of the 1st mill. CE. Its spread into this non-native, arid environment is emblematic of the trade dynamics that took place at this pivotal point in human history. Due to its geographical location, the Arabian Peninsula is connected to both the Indian and African trading spheres, making it complex to reconstruct the trans-continental trajectories of plant diffusion into and across Arabia in Antiquity. Key questions remain pertaining to: (1) provenance, i.e. are plant remains of local or imported origin and (2) the precise timing of cotton arrival and spread. The ancient site of Mleiha, located in modern-day United Arab Emirates, is a rare and significant case where rich archaeobotanical remains dating to the Late Pre-Islamic period (2nd–3rd c. CE), including cotton seeds and fabrics, have been preserved in a burned-down fortified building. To better understand the initial trade and/or production of cotton in this region, strontium isotopes of leached, charred cotton remains are used as a powerful tracer and the results indicate that the earliest cotton finds did not originate from the Oman Peninsula, but were more likely sourced from further afield, with the north-western coast of India being an isotopically compatible provenance. Identifying the presence of such imported cotton textiles and seeds in southeastern Arabia is significant as it is representative of the early diffusion of the crop in the region, later to be grown extensively in local oases.

## Introduction

The Arabian Peninsula is situated at the crossroads of several geographic regions: eastern Africa, the Indian subcontinent, the Near East and is well connected to the Mediterranean. Owing to this critical position, Arabia, through its inhabitants and traders, has contributed to the diffusion of animal and plant species in the western Indian Ocean bordering regions since prehistoric times^1–3^. At the turn of the 1st millennium BCE-CE, intercultural communication and long-distance trade between the Arabian Peninsula and Indian and African sub-continents was well-established. Cotton (*Gossypium* sp.), a plant of tropical origin appeared on the Arabian Peninsula during this period^4,5^, and provides important evidence for such exchange.

Cotton belongs to the Malvaceae family and the genus *Gossypium*, which comprises approximately 45 herbaceous and woody perennial wild species growing at tropical and sub-tropical latitudes. Two species were domesticated in the Old World. *G. arboreum* on the Indian subcontinent, was probably domesticated somewhere in the north-western Indian sub-continent, between the 6th and the 4th mill. BCE^6^, before spreading to the south of the Indian subcontinent from the 3rd mill. BCE onwards^7^. *G. herbaceum* was domesticated in Africa. The precise place of origin and the exact chronology remain unknown, but the earliest archaeological traces of cotton use are located in north-eastern Africa and date back to the extreme end of the 1st mill. BCE-beginning of the 1st mill. CE^4,8^. Textiles and texts show importation and introduction of cotton in Mesopotamia and Bahrain Island during the 1st mill. BCE^9–11^. The first evidence of archaeological cotton on the Arabian Peninsula (apart from Bahrain Island) appears at the turn of the era at Hegra, in current north-western Saudi Arabia. The high proportion of cotton seeds among the archaeobotanical assemblages, their location in all parts of the residential area and their distribution from the late 1st to the early 4th c. CE likely indicates that cotton was cultivated locally in the oasis during Antiquity^4,5^.

During the beginning of the 1st mill. CE, the growing number of seeds, textiles and texts in north-eastern Africa, the Middle East and the Indian subcontinent point to several production centres and foci of cotton diffusion. This spread of cotton into arid Arabian environments is a proxy for the trade dynamics that were intensifying during this period and is evidence for interaction between Arabian and external settlements. The ancient trade of goods and materials from one region to another can be traced using the radiogenic properties of strontium and its isotopic ratio (^87^Sr/^86^Sr)—either by measuring the strontium isotope composition of the humans that migrated with the materials^12^ (using tissues such as tooth enamel, bone, etc.), or more directly by measurement of the materials themselves. Strontium (Sr) is a particularly useful geochemical tracer as it is passed from bedrock to plants with no significant fractionation through biological processes^13,14^. Therefore, plants reflect the geological region in which they grew, which itself is dependent on the composition and age of the bedrock and can be locally characterised by specific ^87^Sr/^86^Sr values. Determining the provenance of modern plants i.e. food authentication, has proven successful for several different botanical materials and products (see Coelho et al.^15^ for a review), including the use of stable isotopes to investigate the provenance of modern cotton^16^. Archaeological studies are less numerous, but include the study of glass (made of plant ash and sand)^17,18^, cereals^19^ and grape seeds^20^. Radiogenic strontium analysis of ancient textiles has proven to be a successful tool for determining provenance^21–29^.

**Figure 1:**
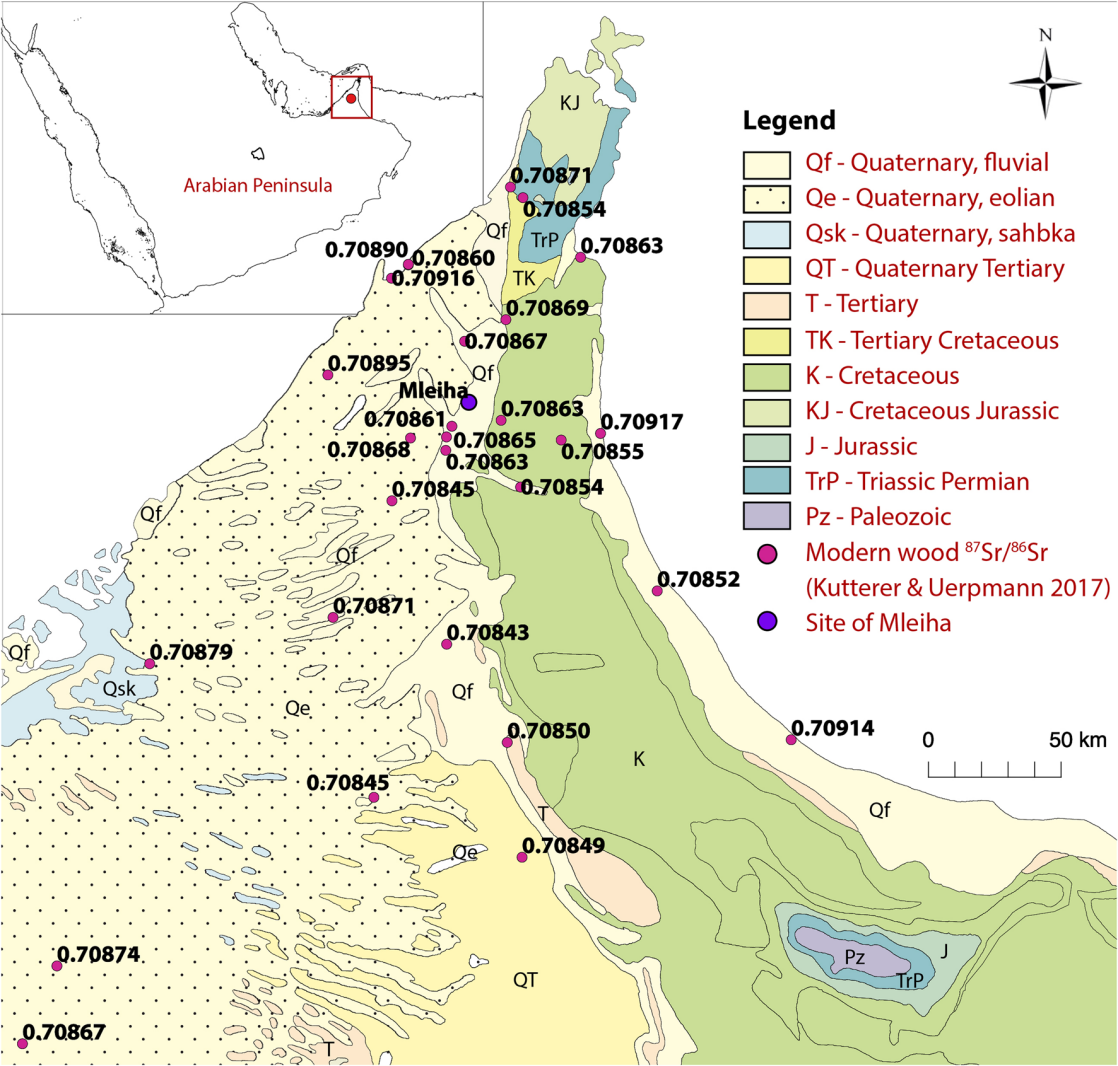
Geological map of the study area, created using QGIS 2.8.2 Wien^39^ with a USGS Open Data basemap^40,41^. Strontium isotope values of the modern wood are those measured by Kutterer and Uerpmann^32^.

The relative abundance of strontium isotopes (^87^Sr and ^86^Sr) in a bedrock is linked to the initial presence of the radioactive isotope of Rubidium (^87^Rb). Through time, ^87^Rb decays forming additional ^87^Sr, and consequently increases the ^87^Sr/^86^Sr ratio, explaining the link between ^87^Sr/^86^Sr and bedrock age. While the geological setting of a region can be used to estimate the local range in strontium isotope values, it is not always an accurate representation of the local bio-available range (the strontium incorporated into plants and animals)—often expressed as a mean ^87^Sr/^86^Sr value ± 2 standard deviations (SD)^30,31^, measured from plants that were known to have grown locally. This assessment is used to garner a local reference dataset with which archaeological material can be compared, as has been done for the Oman Peninsula^32^. Bronze Age fauna have also been used for this purpose on a broader scale, covering areas of western Arabia, Central Asia, and south Asia^33^. Here, we aim to determine if the ancient cotton seeds and textiles recovered from Mleiha (2nd–3rd c. CE) (Fig. 1) (see Supporting Information for full geological description), a key site in Arabia (U.A.E.) during the Late Pre-Islamic Period, were grown close to the site or traded from further afield, based on their strontium isotope composition—while in tandem investigating the corresponding archaeological evidence for Pre-Islamic trade networks in the region. A fire at the site enabled exceptional preservation conditions of a great diversity of organic remains^34^, including cotton seeds and textiles, providing a rare opportunity to examine a snapshot of the Late Pre-Islamic period, at a site which was accessible from, and had connections with, other settlements on the Arabian Peninsula^35^, and much further afield^36–38^.

## Results

### Strontium isotopes

Strontium isotope values from archaeological cotton seeds from Mleiha exhibit a range in values between 0.7097–0.7128 (mean of 0.7110 ± 0.0016, n = 3) (Supporting Information, Table S1). Sr isotope (^87^Sr/^86^Sr) values from processed cotton textiles have an overlapping but wider range of 0.7088–0.7141 (mean of 0.7105 ± 0.0020, n = 6). The strontium isotope values of hydrofluoric acid (HF) and hydrochloric acid (HCl) leachates from four of the textile remains were analysed to determine the composition of any adhered contaminants (Supporting Information, Table S2). All bar one of the acid leachate values are less radiogenic than those of the residual textiles [SR 434 HF (0.7089) had a slightly more radiogenic value than the residual cotton (0.7088)]. A ratio of 0.7098 was measured from one raw cotton boll. Two archaeological seeds of wheat (*Triticum aestivum/durum/turgidum*) and barley (*Hordeum vulgare*), which were almost certainly grown locally (based on their presence in huge quantities and their common occurrence at sites in Arabia since the Bronze Age^42^), have the same value of 0.7087. This value corresponds to the lower part of the isotopic range in strontium measured from archaeological cotton seeds and cotton textiles. Finally, a local modern sample of wood charcoal has a measured value of 0.7086; this suggests a tight range in local values.

### Radiocarbon dates

The radiocarbon ages of the samples range between 1820 ± 30 and 1863 ± 20 BP. The calibrated ages range from 83 to 321 cal CE. As the samples are short lived and come from the same context we consider them to be contemporaneous. Radiocarbon ages passed the Chi^2^ test (T = 1.7 11 n = 6), indicating that they were indeed not significantly different and were combined using the R Combine function in Oxcal, providing a calibrated range of 127–224 cal CE (Supporting Information, Table S3).

## Discussion

### The chronology of cotton in Arabia

The radiocarbon dates of the cotton found at Mleiha represent the earliest known evidence of cotton on the Oman Peninsula, while post-dating references to cotton in Bahrain (6th–4th c. BCE^43^) and overlapping in chronology with cotton finds at Hegra, on the western Arabian Peninsula (see Supporting Information, Fig. S4 for chronological details). In the late 19th c. CE, small scale production of cotton in Oman is mentioned, but it is noted that it would not have been enough to satisfy the local demand^44^. Therefore, the cotton remains at Mleiha may signify the initial introduction of cotton into the region, where there is no earlier evidence for cultivation of this crop, and yet later adaptation of the local desert environment allowed growth of cotton in oases.

### Comparing the local Sr range to archaeological cotton finds

**Figure 2:**
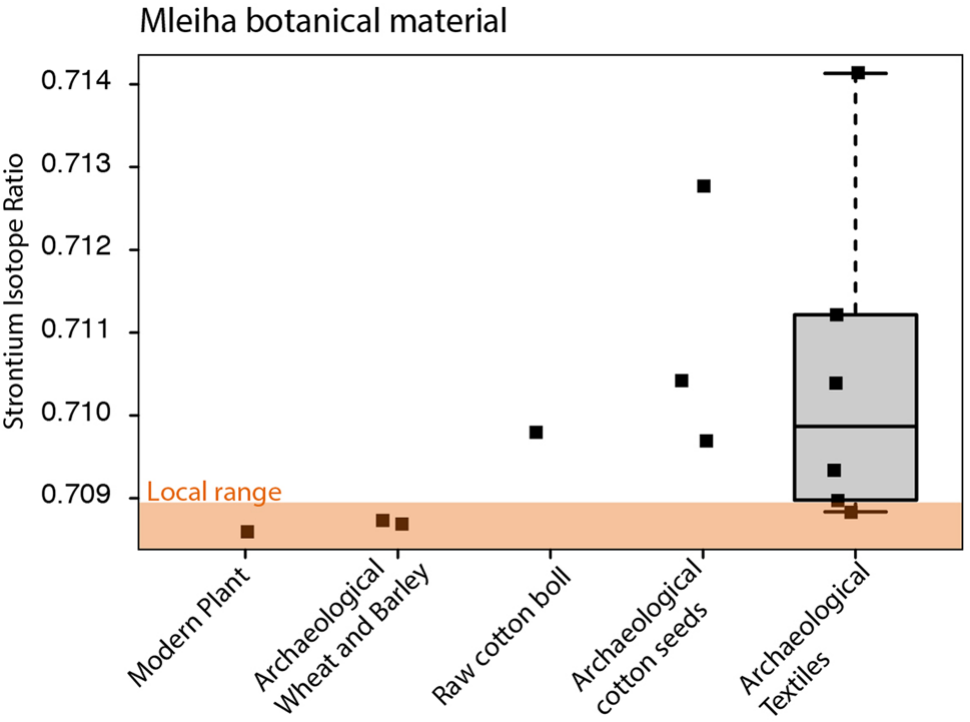
Strontium isotope variation of the botanical remains found at Mleiha. Local range (marked by orange band) is defined by modern vegetation (n = 25) from southeastern Arabia^32^.

The ^87^Sr/^86^Sr of three archaeological cotton seeds, six cotton textiles and one boll are more radiogenic and have a greater degree of variance than a local modern plant sample analysed from Mleiha (Fig. 2) (for sample details, see Supporting Information, Table S4). In tandem with the modern sample, archaeological non-cotton plants (wheat and barley), that almost certainly grew locally, were used as an additional proxy of local strontium isotope values for the site and in doing so, specifically address what the strontium composition was during the period of occupation. The values of both local modern and archaeological cereals are very similar to one another, suggesting no notable changes to the strontium isotope values have occurred from potential modern contaminants e.g. fertiliser use.

Wood of trees and shrubs was used by Kutterer and Uerpmann^32^ to establish a database of bioavailable strontium for the southeast of the Arabian Peninsula. The strontium isotope ratios of these wood samples (n = 28) collected in the United Arab Emirates range from 0.7084 to 0.7092 with a mean of 0.7087 ± 0.0002, or when three statistical outliers are excluded, a range of 0.7084 to 0.7090 with a mean of 0.7086 ± 0.0001 (n = 25). These outliers, which exhibit the most radiogenic values (0.70917, 0.70916, 0.70914), are from near-coastal plants and correspond with the Sr isotope composition of modern seawater (0.709179 ± 0.000002^45^, the ocean being homogenous in ^87^Sr/^86^Sr), likely resulting from sea spray aerosol deposition. More specifically, wood samples collected in close proximity to Mleiha (n = 5) have a range from 0.7086 to 0.7087^32^. These ratios are in agreement with the values observed by Gregoricka^33^ from Bronze Age fauna from the UAE: 0.7085–0.7090. The local strontium isotope values for Mleiha and the UAE are relatively unradiogenic and such values are common over much of southeastern Arabia, extending considerable distances from the site itself (Fig. 1).

Given that the strontium isotope values of the archaeological cotton material, both seeds and textiles, are distinct from the defined local and extended region’s strontium isotope composition, it is unlikely that the archaeological cotton was grown locally, despite the fact that agronomically it would have been possible to do so. Mleiha is situated in an environment with similar climatic conditions to those of Hegra and Qal’et al.-Bahrain, sites at which cotton is postulated to have been grown (Supporting Information, Fig. S5). However, as the local plant values are consistent with the Quaternary sediments that underlay the region and given the relatively homogenous geological context of sedimentary deposits, values as radiogenic as those that have been measured from the textiles (0.7088–0.7141, n = 6) and cotton seeds (0.7097–0.7128, n = 3) would not be expected if grown in the setting of Mleiha. Textile sample SR 434 (^87^Sr/^86^Sr = 0.7088) marks the lower limit of the range exhibited from the archaeological textiles and does not overlap with local values if compared only with modern wood samples collected from close proximity to Mleiha (n = 5) (which range from 0.7086 to 0.7087^32^), but is within the range observed from entire UAE more broadly (range from 0.7084 to 0.7090, n = 25 excluding outliers^32^) so could have been grown on the Oman Peninsula, considering the isotope value alone. It should be noted that the textile samples were leached successively in HF and HCl to remove burial contaminants (see “Materials and methods” for details). As all (n = 7) bar one of the acid leachates are less radiogenic than the residual textiles (Supporting Information, Table S2), should the leaching procedure have been unsuccessful in entirely removing any exogenous strontium, the incomplete decontamination would result in an underestimation of cotton grown outside of the local area^46,47^. This is because burial contamination would skew the strontium isotope signature towards that of the local region in which they were found. Therefore, measurement of the Sr isotope composition of these archaeological textiles provides a conservative indication of cotton that is unlikely to have been cultivated in the local area.

### Textual and archaeological evidence of trade links

The archaeological material found at Mleiha during the last phase of occupation (PIR-D, 150–250 CE) shows that the population at the site was involved in the long-distance trade networks that were established across the Indian Ocean during this period^36,37,48–50^. This archaeological material evidence, used in combination with the geochemical data, is particularly useful for identifying likely source regions and sites with which residents of Mleiha potentially traded. Specifically, this context revealed Indian pottery, as well as some East-African lamps and Egyptian amphora—confirming strong trade connections by that point^36–38^. Seeds, textiles and textual sources point to several potential cotton production regions at that time, namely the Indian sub-continent, Mesopotamia, the north-western Arabian Peninsula and north-eastern Africa (see an overview in Bouchaud et al.^51^, Supporting Information, Fig. S5).

Indian pottery observed in building H at Mleiha have been extensively studied using X-ray fluorescence (XRF) spectrometry. Sherds from several archaeological sites in western India were compared with coarse-ware vessels sampled at Mleiha and the results show that Maharashtra and Gujarat States of India were probable source areas for at least two types of wares and acted as a focal point of connection between major caravan routes^49^. Archaeobotanical and textile occurrences observed in the Indo-Pakistani area demonstrate the presence of millennia-old cotton agriculture from the Neolithic period until present-day^7^. Moreover, the discoveries of peppercorns (*Piper nigrum*) from Kerala State in southern India and grains of Asian rice (*Oryza sativa*) from western India in building H at Mleiha also highlight the trade activities with the Indian sub-continent^52^. Given this evidence for established trade links between the Indian sub-continent and Mleiha, it constitutes a probable place of cultivation for the cotton found on site.

Textual references from the regions surrounding the north-western Indian Ocean also allow identification of potential cotton production and trade centres that may have provided the cotton remains. The anonymous author of the *Periplus Maris Erythraei* (*PME*), a Greek handbook compiled during the 1st c. CE by an Alexandrian sailor as a guide for merchants engaged in long-distance trade in the Red Sea and Indian Ocean, mentions several ports/cities where cotton was sold or traded in the same regions^53^, see details in Supporting Information, Table S5, Fig. S5. Although cotton may have been traded from these sites, it should also be noted that it may have been grown in regions far from trading towns/ports. The *PME* also indicates several cotton production and/or manufacturing regions, namely Ozênê (Madhya Pradesh), Masalia (Andhra Pradesh) and Abêria (Between Barygaza and Ozênê)^53^ (see details in Supporting Information, Table S5).

The presence of Indian pottery and other distinctive food products, such as rice and pepper, suggest an origin from the Indian sub-continent, but an East African or Mesopotamian origin for the cotton remains cannot be completely ruled out given the presence of the Egyptian amphorae, glass vessels, bitumen and Nubian lamps on site^34,38,54^. However, the bulk of the archaeological and archaeobotanical material found at Mleiha is more suggestive of a strong Indo-Arabian trade network.

### Potential source regions based on isotopic data

**Figure 3:**
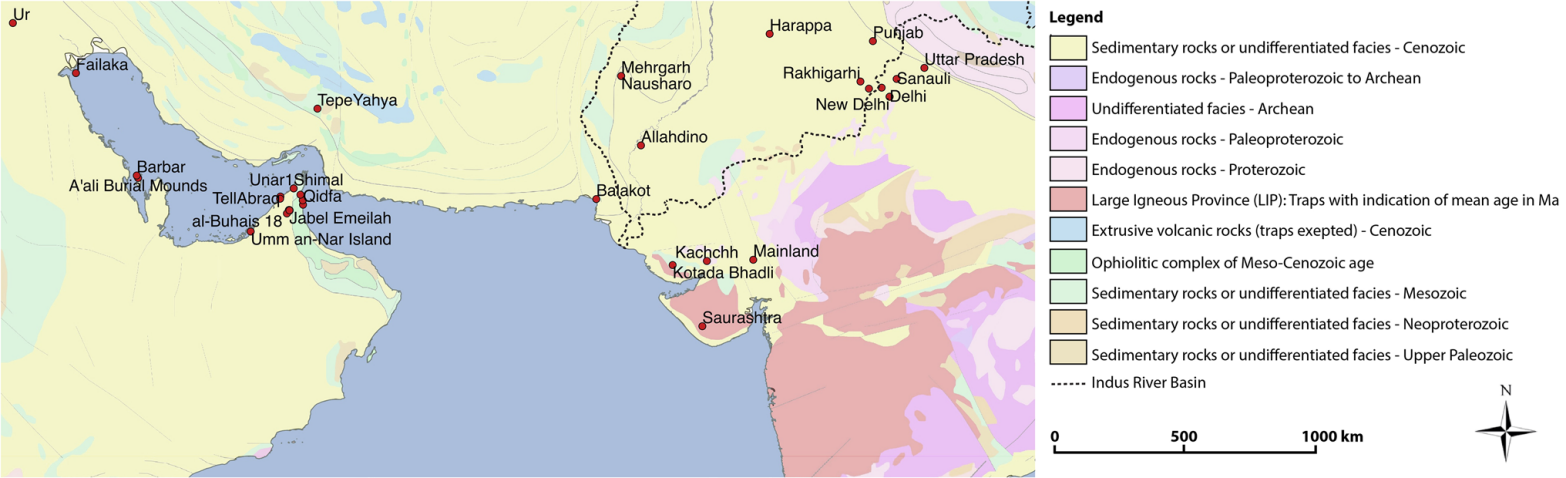
Sites mentioned in the text that have associated faunal, human and/or botanical strontium isotope data. The geological basemap^56^ was accessed using OneGeology^57^, with the addition of Indus River Basin outline^58^ and was edited using QGIS 2.8.2 Wien^39^. For further site details see Supporting Information, Table S6.

A scarcity of bio-available strontium isotope data for east Africa, particularly north-eastern Africa, makes it difficult to define regions with comparable strontium isotope ranges to that of the cotton at Mleiha. Although for such a diverse and widespread area, such a range in values is certainly possible, as has been shown in a study of elephant ivory provenance^55^. That being said, given the widespread evidence for the trade of material goods between the Indo-Pakistani region and eastern Arabia, we focussed on examining existing bioavailable strontium isotope data for these areas, as well as the regions surrounding the Persian Gulf (see Fig. 3 for locations and Supporting Information, Table S6 for compiled data). Although such datasets are not spatially comprehensive, the information they provide aids with honing in on likely source regions, from further afield, for cotton and its products.

**Figure 4:**
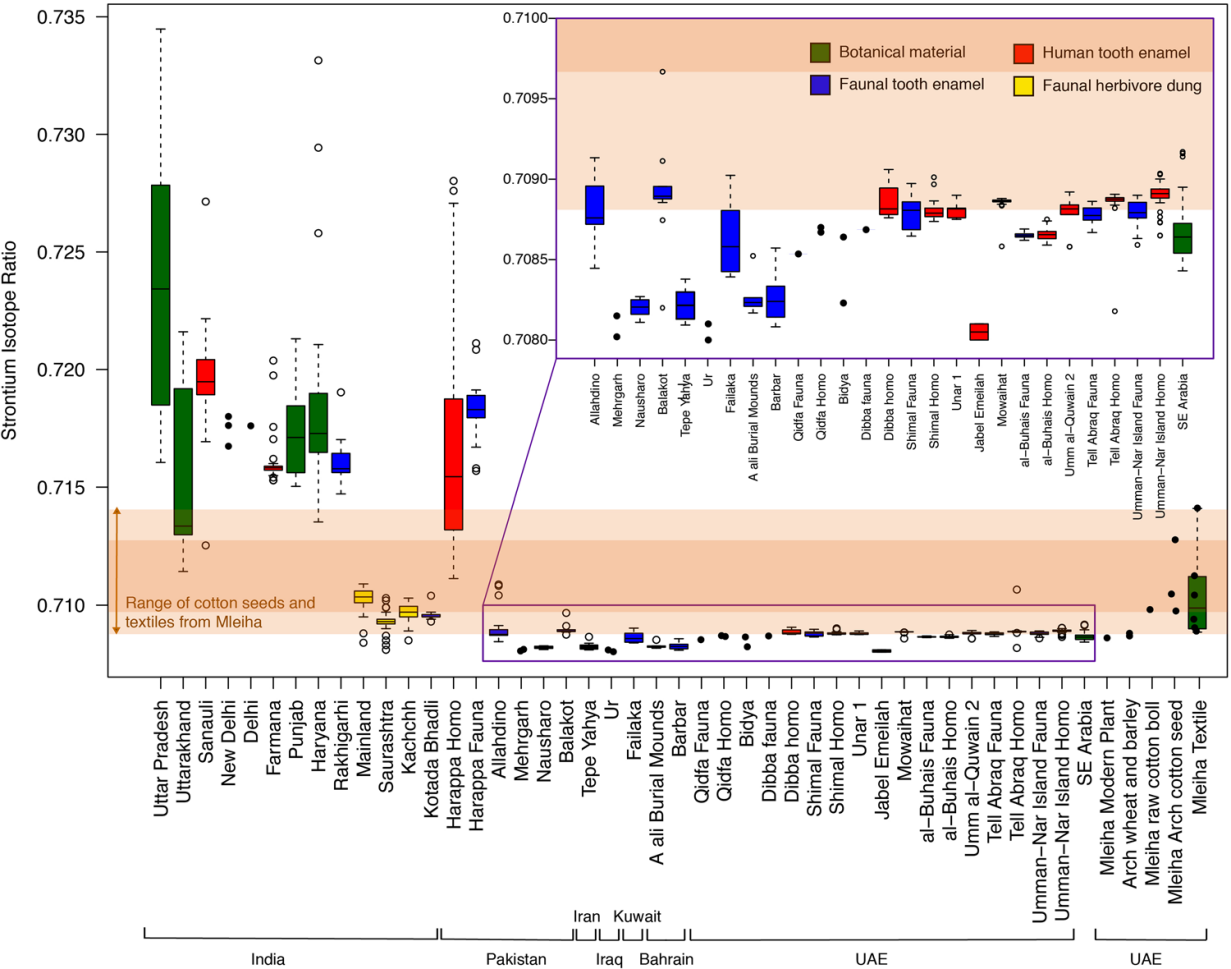
Strontium isotope variation across southeastern Arabia and the Indo-Pakistani region, including the botanical remains found at Mleiha. Data^32,33,60–65^. Dark and light orange bands represent the range of archaeological cotton seeds and textiles from Mleiha, respectively.

Two archaeological sites in Iran and Iraq, Tepe Yahya and Ur respectively, were found to have values too poor in radiogenic ^87^Sr to correspond to that of the Mleiha cotton remains (Fig. 4). Similarly, while there is some evidence for cotton use in Bahrain in Antiquity, in the form of textile remains (600–400 BCE)^43^ and one textual reference (325–324 BCE)^59^, the ^87^Sr/^86^Sr values measured from Bronze Age fauna, suggest that the local strontium isotope signature for Bahrain (mean of 0.7083 ± 0.0001, n = 20)^33^ is incompatible with the Mleiha cotton.

While the bioavailable strontium isotope values of southeastern Arabia have a small strontium isotope range^32^ (inconsistent with that of the archaeological cotton), values from western India exhibit a greater degree of spatial isotope variation that covers that observed in the Mleiha cotton. Several studies from the vast Indo-Pakistani region have measured the strontium isotope ratios of archaeological faunal and human enamel and modern herbivore dung^32,33,60–67^ (see Fig. 3 for geographical locations and Fig. 4 for isotope comparison of site values) and taken together, these data provide an overview of potential source regions of the cotton found at Mleiha.

The Indus Valley, one potential source region, consists of Tertiary and Quaternary alluvium deposited by the Indus River and the geology of the surrounding regions spans a large range of ^87^Sr/^86^Sr values (0.708–0.822)^63^ (see Fig. 3 for river basin location). The Sr isotope composition of the region is divided, with north-western young volcanic and ultramafic rocks dominated by low ^87^Sr/^86^Sr sources (0.704–0.707), and northern sites influenced by highly radiogenic Himalayan sediments (> 0.716). To the west, intermediate radiogenic sedimentary rocks control the strontium composition (0.710–0.712)^68^. Analyses of archaeological fauna support that the Indo-Gangetic Plains are suitably geochemically distinct for application to archaeological provenance studies over wide scales; fauna from the region are broadly consistent with the existing geological strontium isotope data, suggesting that both proxies are useful in determining local ranges of isotopic variation^64^. Fauna from Harappa, a site in Pakistan and many of the sites in India have significantly higher and more variable strontium isotope values than other sites examined here (Fig. 4)^63,64^, which in most cases are too radiogenic to have been the regions where the cotton was grown. It should be noted that domestic animals and/or humans are not an ideal medium for characterising the local bioavailable range as they may have consumed imported food or have had an extensive home range that averages values of a wider region than what could be considered ‘local’^13,69^. However, compiling existing measurements of ^87^Sr/^86^Sr values derived from archaeological animal bone can provide an approximation and compensates for regions where there is a lack of data.

Many of the notable trading sites during this period in the western Indian region (Supporting Information, Fig. S5) are located on the Deccan Trap basalts (red-coloured region in Fig. 3). Uncontaminated Deccan Trap basalts have ^87^Sr/^86^Sr of ∼0.704^70^ but ranges of 0.706–0.710 are not uncommon due to crustal silicate contamination^71^. Rivers draining these basalts and plants growing on such terrains would be expected to be poor in radiogenic ^87^Sr. The Bushe Formation basalts have the highest Sr ratios (0.713–0.719) among the Deccan Trap basalts, being the most contaminated by granitic crust^72^, and can be found widely across the central and eastern sections of the Deccan Trap basalts. The cotton found at Mleiha (seeds and textiles, with means of 0.7110 ± 0.0016 and 0.7105 ± 0.0020, respectively) do not correspond with such ranges observed from the Deccan Trap basalts, with certain regions mostly having values that are too low, and in some cases (such as the Bushe formation) too high, to correspond with the measured strontium isotope composition of the textiles and seeds. However, faunal herbivore dung from Saurashtra, a site in western India which overlies Deccan Trap Basalts exhibited a range of 0.7093 ± 0.0004 (n = 58)^61^. The material from this site, as well as Mainland and Kachchh in the Gujarat region of India^61^ share an overlapping range of values with the cotton at Mleiha (within the orange bands of Fig. 4). These isotope data, combined with the actual presence of ancient cotton production in the Gujarat region and archaeological evidence for connection between this region and southeastern Arabia put forward a strong argument for the cotton at Mleiha having been sourced in western India.

In any case, the travel distances involved with trade between Arabia and the Indo-Pakistani regions should not be underestimated. We cannot say if the material was imported from a single location with a relatively high degree of local variance, or multiple, geochemically distinct sites. Nor can we pinpoint specific locations based on the geochemical values alone, given the vast geographic extent. But any location that exhibits a range which encapsulates that of the Mleiha cotton (such as Gujarat and the west, near-coastal Indus Valley), can be considered as potential source regions. Nonetheless, it can be concluded that unlike the Mleiha archaeological wheat and barley and the local modern plants—the cotton seeds and textiles are not from the immediate vicinity of Mleiha, nor are they from another part of southeastern Arabia. They most likely came from vast distances away, likely in the western Indian provinces.

## Conclusions

The strontium isotope values of the archaeological cotton seeds and textiles are inconsistent with those observed from modern plants growing in the immediate vicinity and extended region surrounding Mleiha (UAE), the site from which they were excavated. They can therefore be considered as non-‘local’ and derive from a location with slightly but significantly higher ^87^Sr/^86^Sr values. Given that the low radiogenic strontium isotope values that characterise the site of Mleiha are common to much of southeastern Arabia, it appears that the more radiogenic, non-local cotton remains, were likely sourced from considerable distances away, with regions of modern-day western India being the most likely potential place of origin. This hypothesis is based on the fact that comparable strontium isotope values can be found within such regions, and archaeological and textual evidence points towards developed cotton production centres in these Indo-Pakistani regions. Furthermore, archaeobotanical and archaeological material from Mleiha clearly indicates a trade network between southeastern Arabia and India. These independent data sources show that long-distance sea-borne trade links between the Oman peninsula and India were flourishing during the 2nd–3rd c. CE. The presence of such non-local textiles and seeds found on site also suggests that the growing of cotton in nearby oases was not yet common practice, or at least in its infancy.

## Materials and methods

### Site and samples

The site of Mleiha is situated in the Oman peninsula (lat. 25.119, long. 55.877) (Fig. 1). The site was occupied during the Late Pre-Islamic period (PIR), that is the 3rd c. BCE to the mid 3rd c. CE. A fortified building in sector H, dating to the later phase of occupation [PIR-D (150–250 CE)] (Supporting Information, Fig. S1) was destroyed in a fire which allowed the exceptional preservation of botanical assemblages including common oasis crops, like barley (*Hordeum vulgare*), lentil (*Lens culinaris*) and date palm (*Phoenix dactylifera*)^34^ and foreign taxa such as pepper (*Piper nigrum*) and Asian rice (*Oryza sativa*) which indicate strong trade connections between Mleiha and the Indian subcontinent^52^, but also cotton remains which are the object of this study. In total, 31 whole seeds and 79 fragments as well as 7 raw fibre clusters have been retrieved in the building. For isotope analyses, three separate seeds as well as a boll have been selected for study, as well as six pieces of textile (see Supporting Information, Tables S1, S4 for full sample details). In order to gauge the local isotope range that exists within the region, one modern wood fragment of a shrub from the Amaranthaceae family, gathered on the alluvial plain of Mleiha, was selected. Furthermore, two archaeological caryopses (grains) of barley (*Hordeum vulgare*) and wheat (*Triticum aestivum/durum/turgidum*), were analysed.

### Pre-treatment of botanical remains: removal of contaminants by leaching

The entire acid digestion process and subsequent Sr purification were achieved under a class 100 laminar flow hood in a class 1000 clean room. Exogenous Sr was removed from carbonized cotton seeds by leaching them in 5 ml 6 M HCl for 24 h, an adapted procedure of that found to be the most effective of several different leaches at removing some but not all contaminants^46^. The solutions were centrifuged, the supernatant removed and the leached seeds were rinsed in ultrapure Milli-Q water three times and dried.

In the case of the recovered textiles—microscopic inspection revealed the presence of sand grains embedded between the fibres. A room temperature 20% hydrofluoric (HF) acid leach for 1 h, under ultrasonic treatment, was used to remove these grains. Subsequently, the HF supernatant was removed, leaving behind the residual textile sample. This portion was then rinsed three times with ultrapure Milli-Q water. The same leaching procedure was used again with 1 M HCl in place of the HF. The residual textile was then dried, as were the leachates. This procedure followed an adapted method outlined in Frei and Bjerregaard^21^, which was designed to decontaminate textile samples of silicates that are rich in Sr.

### Digestion and ion chromatographic procedures

The residual plant samples were weighed into porcelain crucibles with lids and ashed in a muffle furnace at 550 °C. The ashed sample was dissolved in a 1:1 mixture of 8 M HNO_3_ and 30% H_2_O_2_ for 24 h at 100 °C for 18 h in Teflon, after which the solutions were dried down on a hotplate at 80 °C. Samples were taken up in a 3 M HNO_3_ solution and loaded onto cation exchange columns that had been charged with SrSpec (Eichrome Inc.) resin which has been intensively pre-cleaned. Strontium was eluted in 2.5 ml ultrapure Milli-Q.

### Mass spectrometry

Strontium isotope analyses of the archaeological seeds (n = 5) and textiles (n = 6), the raw cotton boll (n = 1) and the modern plant sample (n = 1) were performed on a ThermoScientific Neptune^Plus^ Multi-Collector Inductively Coupled Plasma Mass Spectrometer (MC-ICPMS), at the Laboratoire des Sciences du Climat et de l'Environnement (LSCE, France). The purified strontium fractions were adjusted to a strontium concentration of 20 μg/l by dilution with 0.5 N HNO_3_. The LSCE has recently updated the analytical method for measurement of Sr isotopes using MC-ICPMS previously published by Palmiotto et al.^73^. Notably, an APEX Omega is now used as the desolvating system with a 100 µl/min nebulizer. The sensitivity for a 20 ppb Sr solution is around 30 V on the ^88^Sr peak and the blank contribution (0.5 N HNO_3_) is around 0.05 V. The samples and standards are analysed in a static multi-collection mode in a single block of 90 cycles with an integration time of 4 s per cycle. No isobaric correction is required for Ca dimers/argides and only minor corrections for ^87^Rb to ^87^Sr are considered. A correction is also applied for krypton (Kr) isobaric interferences (^86^Kr on ^86^Sr). In general, the chemical purification of Sr renders the effects of doubly charged Rare Earth Elements (REE) on the Sr isotope masses negligible due to the very low absolute REE abundances. Nevertheless, the REE effects, especially on the ^82^Kr/^83^Kr ratio, are systematically checked. The reproducibility of the ^87^Sr/^86^Sr measurements was evaluated through replicate analyses of the NBS 987 standard. A mean value of 0.710231 ± 0.000005 (n = 27) was obtained in this study. Isotopic ratios were corrected using a standard-sample bracketing method and normalised to the NBS 987 standard value of 0.710245 and the corresponding external reproducibility of 14 ppm (2σ) was determined for this run. For each sample, the ^87^Sr/^86^Sr value is reported with a 2σ uncertainty, taking into account the standard reproducibility and the measurement standard error of each sample.

### Radiocarbon dating

Four short lived samples (three grape pips and one indeterminate seed) and two pieces of cotton textiles were selected from different contexts of Building H. They were prepared for radiocarbon dating at the ^14^C lab of the Muséum national d'Histoire naturelle (MNHN) using the classical acid-alkali-acid (AAA) method. Briefly, the samples were first immersed in 1 N HCl for 1 h at room temperature and then rinsed in distilled water. They were then immersed in 0.01 N NaOH for 20 min at room temperature and then rinsed. Finally, they were immersed again in 1 N HCl for 1 h, then rinsed and dried overnight at 90 °C. The seeds were combusted in a vacuum line and ca. 1 mg C of purified CO_2_ was sealed in a glass tube, then sent to the Accelerator Mass Spectrometer (AMS).

Graphitization and ^14^C measurements were carried out at the AMS Laboratory at the University of Arizona, Tucson, AZ, USA and at the Laboratoire de Mesure du Carbone 14 (LMC14) at Saclay, France. The textile samples were graphitized using an automated AGE 3 device and ^14^C measurements were performed on 1 mg C targets using the compact AMS ECHoMICADAS at LSCE (Gif-sur-Yvette, France). The radiocarbon ages were calibrated using the Oxcal 4.3.2. software and the IntCal 13 atmospheric curve^74,75^.

## Supplementary Information

Supplementary Information
