# Author Correction: Strontium isotope evidence for a trade network between southeastern Arabia and India during Antiquity

**DOI:** 10.1038/s41598-021-86256-5

**Published:** 2021-03-19

**Authors:** Saskia E. Ryan, Vladimir Dabrowski, Arnaud Dapoigny, Caroline Gauthier, Eric Douville, Margareta Tengberg, Céline Kerfant, Michel Mouton, Xavier Desormeau, Antoine Zazzo, Charlène Bouchaud

**Affiliations:** 1 Archéozoologie, Archéobotanique: Sociétés, Pratiques et Environnements (AASPE, UMR 7209), Muséum National d’Histoire Naturelle, CNRS, CP56, 55 rue Buffon, 75005 Paris, France; 2 Laboratoire des Sciences du Climat et de l’Environnement, LSCE/IPSL, UMR CEA-CNRS-UVSQ, Université Paris-Saclay, 91191 Gif‑sur‑Yvette, France; 3 Institut Català de Paleoecologia Humana i Evolució Social (IPHES-CERCA), Zona Educacional 4, Campus Sescelades URV (Edifici W3), 43007 Tarragona, Spain; 4 Institut Français du Proche-Orient, B.P. 11‑1424, Beyrut, Lebanon

Correction to: *Scientific Reports* https://doi.org/10.1038/s41598-020-79675-3, published online 11 January 2021

This Article contained an error. Camille Noûs, listed as the tenth author on the paper, is fictitious and therefore does not fulfil the requirements for authorship. The name is now removed from the author list. Additionally, the following sentence was added in the Acknowledgements:

"Camille Noûs, a fictitious author, embodies the ideals of the collegial construction of the standards of science through developing the methodological framework, the state-of-the-art, and by ensuring post-publication follow-up (https://www.cogitamus.fr/camilleen.html), and is hereby acknowledged."

