# Supplementary material for: Strontium isotope evidence for a trade network between southeastern Arabia and India during Antiquity
Source: Sci Rep. 2021 Jan 11;11:303. doi: 10.1038/s41598-020-79675-3 (PMC7801716; doi:10.1038/s41598-020-79675-3)
Supplement: Supplementary file 1 — Supplementary Information [file 41598_2020_79675_MOESM1_ESM.docx]

**Supporting Information for**

Strontium isotope evidence for a trade network between southeastern Arabia and India during Antiquity

Saskia E. Ryan^1,2^*, Vladimir Dabrowski^1^, Arnaud Dapoigny^2^, Caroline Gauthier^2^, Eric Douville^2^, Margareta Tengberg^1^, Céline Kerfant^3^, Michel Mouton^4^, Xavier Desormeau^1^, Antoine Zazzo^1#^, Charlène Bouchaud^1#^

^1^Archéozoologie, Archéobotanique: Sociétés, Pratiques et Environnements (AASPE, UMR 7209), Muséum national d'Histoire naturelle, CNRS, CP56, 55 rue Buffon, 75005, Paris, France.

^2^Laboratoire des Sciences du Climat et de l’Environnement, LSCE/IPSL, UMR CEA-CNRS-UVSQ, Université Paris-Saclay, F-91191 Gif-sur-Yvette, France.

^3^Institut Català de Paleoecologia Humana i Evolució Social (IPHES), Zona Educacional 4 Campus Sescelades URV (Edifici W3), 43007 Tarragona, Spain.

^4^Institut français du Proche-Orient, B.P. 11-1424 Beyrut, Lebanon

^#^Equal contribution

*Corresponding author: Saskia E. Ryan

**Email:**  saskia.ryan@mnhn.fr

**This file includes:**

Supplementary text

Figures S1 to S5

Tables S1 to S6

SI References

**Presentation of the site**

The site of Mleiha is situated in the Oman peninsula (lat. 25.119, long. 55.877) (**Fig. S1**), on the western sandy and gravelly foothills of the Omani mountains, also known as el-Hajar. Groundwater tables set in the limestone bedrock are present to the east of the site and fertile loamy deposits offer suitable conditions for maintaining agriculture nearby, as is the case today with extended date palm gardens ^1^. The site was occupied during the Late Pre-Islamic period (PIR), that is the 3^rd^ c. BCE to the mid 3^rd^ c. CE. Excavations have been led by the French Archaeological Mission in the United Arab Emirates from 1986 to 2001, under the direction of R. Boucharlat then M. Mouton (CNRS, France). Four different phases of occupation have been defined according to pottery discoveries during which long-distance trade has been noted: PIR-A (300-150 BCE), PIR-B (150-0 BCE), PIR-C (0-150 CE) and PIR-D (150-250 CE). The later phase is characterized by the construction of two fortified buildings which testify to the general context of unrest in the region ^2–5^.

The fortified building in sector H is situated in the north-east part of the archaeological site. This mud-brick building is almost square in shape (30mx32m) and composed of 15 rooms surrounding and opening onto a central courtyard (**Fig. S1**). Luxury goods show that the building belonged to people of elite status. The loss of such material and the fire that destroyed the building suggest that its sudden abandonment was due to a single violent event. The fire has allowed the exceptional preservation of botanical assemblages including common oasis crops, like cereals, pulses and fruits (date palm (*Pheonix dactylifera*), grape (*Vitis vinifera*), pomegranate (*Punica granatum*)) ^6^ and foreign taxa such as pepper (*Piper nigrum*) and Asian rice (*Oryza sativa*) which indicate strong trade connections between Mleiha and the Indian subcontinent ^7^, but also cotton (*Gossypium sp.*) remains.

The geology of wider southeast Arabia is primarily composed of Quaternary sediments, particularly to the west of the Oman peninsula where sand dunes, gravel fans and sabkas (supratidal coastal zone sediments) are present ^8^ (**Fig. 1**). Large expanses of sand dunes in this desertic region are interjected by outcrops of calcareous sandstone and sandy limestones ^9^. To the east of the region are the Hajar Mountains, surrounded by fan sediments composed largely of dolomite, limestone, and chert – eroded sediments from the Oman Ophiolite Belt of the Hajar Mountains ^9,10^. Specifically, the site of Mleiha sits on such deposits, beneath which is a Late Mesozoic limestone bedrock. Silurian ultrabasic rocks and gabbro, with smaller outcrops of limestone, sandstone, granite, and metamorphic rocks, make up the southern area of the mountain range ^11^.

**Samples**

*Cotton seeds and bolls*

To collect macrobotanical remains, sediment samples were taken from the burnt layers of the building and processed using the flotation method, which permits them to float on the surface and to be gathered with fine-mesh sieves (0.5 mm). In this way, several charred cotton seeds and unprocessed cotton bolls have been picked out from the samples with a binocular microscope Nikon SMZ645, meeting identification criteria, in the archaeobotany laboratory of the National Museum of natural History in Paris.

In total, 31 whole cotton seeds and 79 fragments as well as 7 raw fibres clusters have been retrieved in the building (**Fig. S2**). In addition, five whole and five fragmentary seeds hypothetically identified are present in the assemblage. Two whole seeds still bear fibres on their surface, one of them corresponding to fuzz and the other to lint fibres. A textile fragment attached to one boll may represent storage in the form of a bag. With the exception of three whole seeds found in the room 1530 and two potential raw fibres clusters in the courtyard 1524, all others were recovered from the small room 1500 near the entrance. All cotton remains represent less than 1% of the whole building archaeobotanical assemblage (4% in the 1500 room assemblage). For isotopic analyses, three fragmentary seeds as well as a boll have been selected from this latter room.

*2.2.2 Cotton textiles*

Considerable corpus of charred textile remains have been unearthed from the building and only a preliminary analysis has been undertaken to date. Among the total of 190 pieces of textile studied, 118 of them correspond to cotton (see **Fig. S3** for example specimen). They were present in almost all the rooms of the building. Most of them have a common plain weave tabby twisted Z, while some of them are twisted in S/Z or only S. Six pieces of textile from the rooms 1500 and 1529 as well as the courtyard 1524 have been selected for isotopic analyses (**Table S3)**.

2.2.3. Other plant samples

In order to gauge the local isotopic range that exists within the region, one wood fragment of modern shrub from the Amaranthaceae family, gathered on the alluvial plain of Mleiha, was selected. Furthermore, archaeological caryopses (grains) of barley (*Hordeum vulgare*) and free-threshing wheat (*Triticum aestivum/durum/turgidum*), recovered from the room 1540, were also selected. As these seeds represent crops that were likely grown near the site, they are considered to be good indicators for the past local isotopic range.


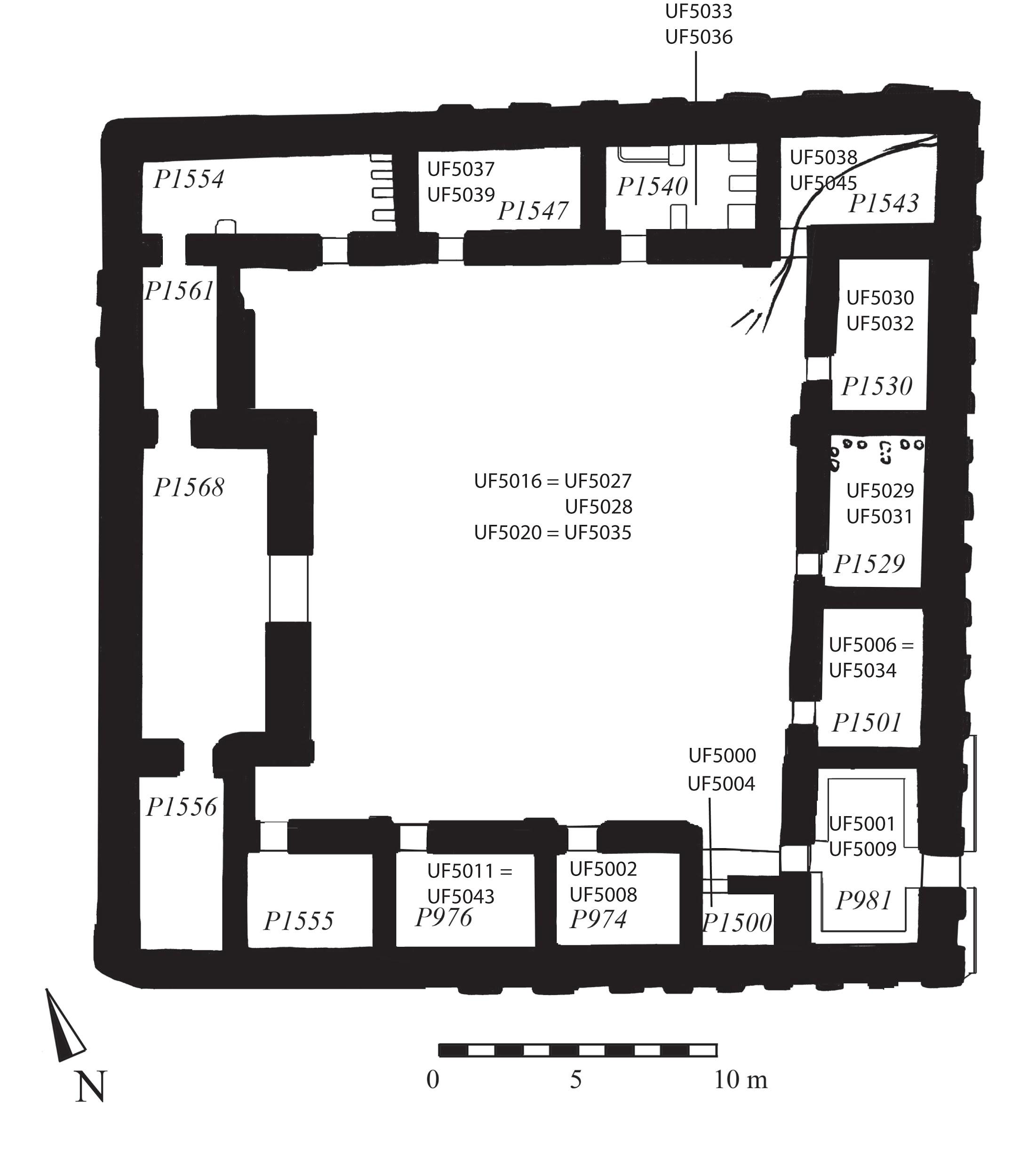


**Figure S1**: General plan of the building H.


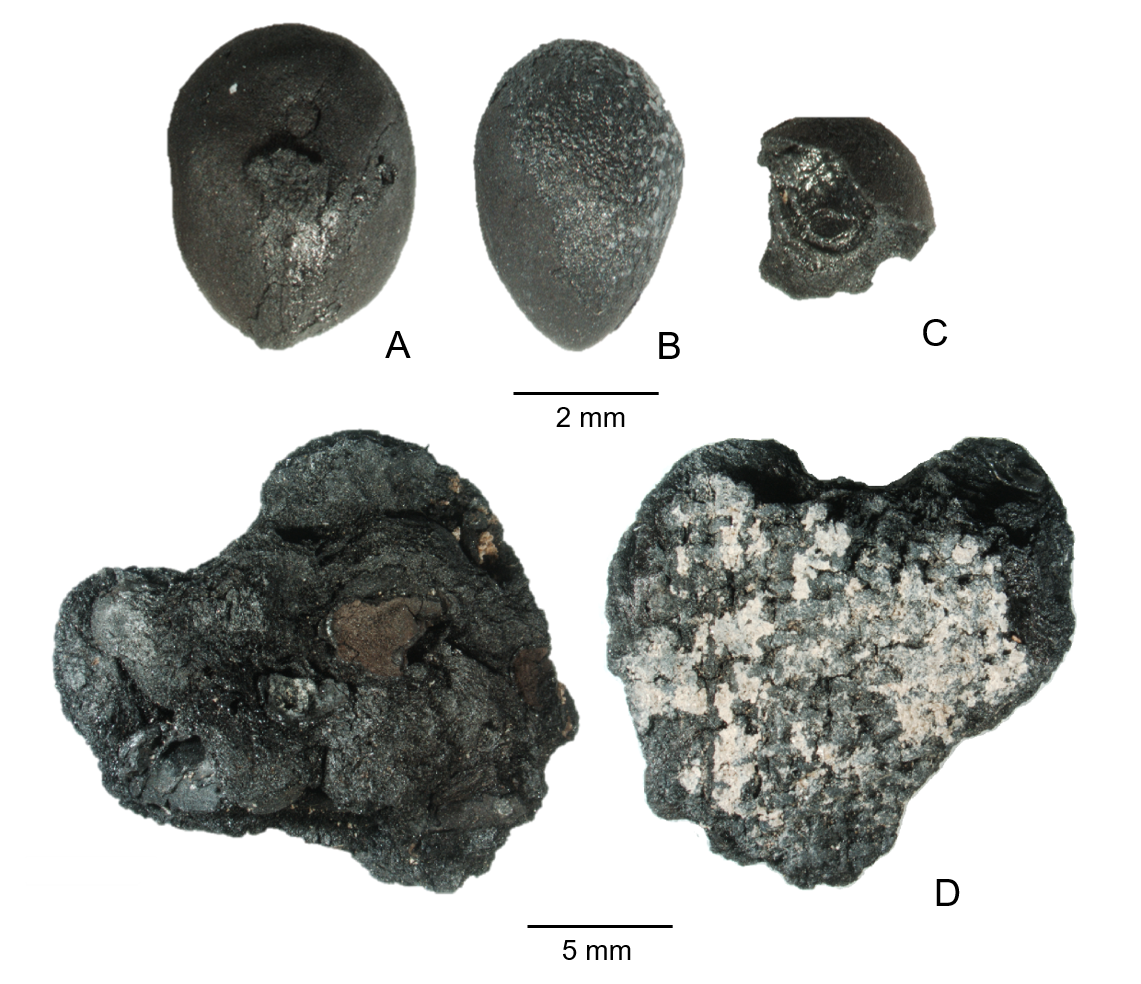


**Figure S2**. Cotton seeds and bolls found in Mleiha. A-B: Two cotton seeds with the distinctive longitudinal raphe (A) and fibres (fuzz) still present on the surface (B); C: Cotton seed fragment with the button-like chalaza on the inside; D: One boll with cotton seeds visible (left) and a piece of textile (bag?) still attached (right).


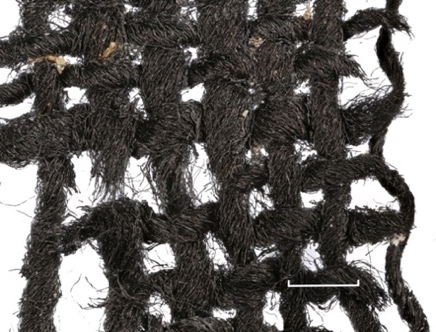


1mm

**Figure S3**. Archaeological cotton fabric from Mleiha (courtesy of the French Archaeological mission of Mleiha, dir. M. Mouton). Small sub-samples of fabric were sectioned from the larger textiles.


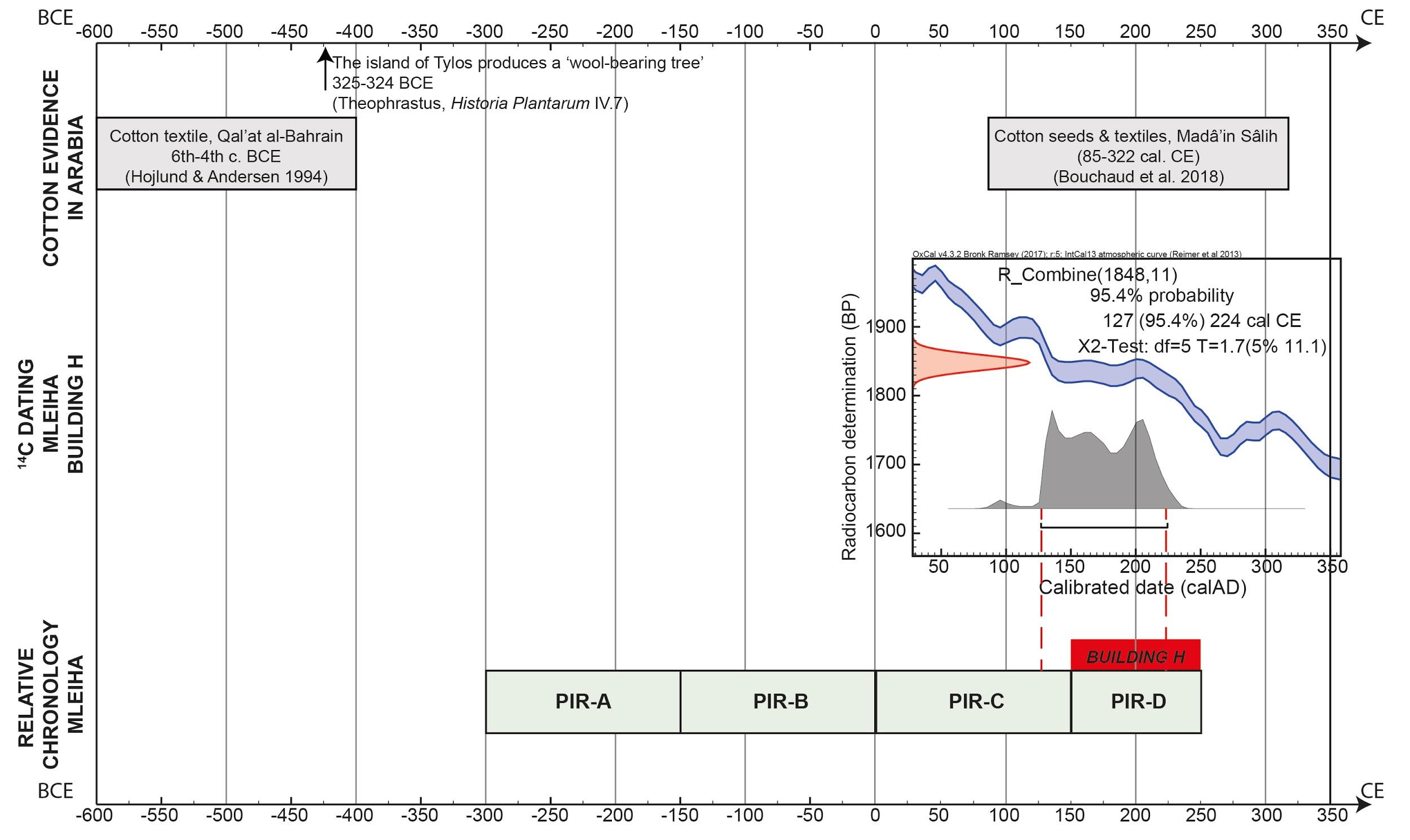


**Figure S4.** Relative and absolute chronological setting of cotton in Arabia and Mleiha occupation.

**Figure S5.** Sites with evidence of cotton, in the form of textual references and/or presence of archaeological cotton seeds/textiles, dating from the mid first mill. BCE to the 3^rd^ c. CE. See **Supporting Information, Table S4** for details.

| Site | Lab ID | Arch/  Modern | Material | Plant | Scientific name | Field ID | ^87^Sr/^86^Sr* | 2σ uncertainty |
| --- | --- | --- | --- | --- | --- | --- | --- | --- |
| Mleiha | SR 395 | Modern | Wood charcoal | Amaranthaceae | Amaranthaceae | ML_AMAR_Bois | 0.708602 | 0.000020 |
| Mleiha | SR 419 | Arch. | Seed | Barley | *Hordeum* | ML UF5036 Hordeum | 0.708738 | 0.000014 |
| Mleiha | SR 420 | Arch. | Seed | Wheat | *Triticum* | ML UF5036 Triticum | 0.708695 | 0.000014 |
| Mleiha | SR 421 | Arch. | Seed | Cotton | *Gossypium* | ML UF5004 10.008 (I) | 0.710422 | 0.000014 |
| Mleiha | SR 422 | Arch. | Seed | Cotton | *Gossypium* | ML UF5004 10.008 (II) | 0.712766 | 0.000020 |
| Mleiha | SR 423 | Arch. | Seed | Cotton | *Gossypium* | ML UF5004 10.008 (III) | 0.709696 | 0.000014 |
| Mleiha | SR 434 | Arch. | Textile | Cotton | *Gossypium* | ML P1500 5004 10184 | 0.708836 | 0.000014 |
| Mleiha | SR 435 | Arch. | Textile | Cotton | *Gossypium* | ML P1524 5020 10135 B | 0.711216 | 0.000014 |
| Mleiha | SR 436 | Arch. | Textile | Cotton | *Gossypium* | ML P1524 5027 10134 | 0.710391 | 0.000017 |
| Mleiha | SR 437 | Arch. | Textile | Cotton | *Gossypium* | ML P1524 5035 10280 | 0.714131 | 0.000029 |
| Mleiha | SR 446 | Arch. | Raw fibers | Cotton | *Gossypium* | ML P1500 5004 10008 | 0.709800 | 0.000014 |
| Mleiha | SR 447 | Arch. | Textile | Cotton/  Linen/ F.L.? | *Gossypium/Linum* | ML P1529 5031 10253.5 | 0.708978 | 0.000014 |
| Mleiha | SR 448 | Arch. | Textile | Cotton | *Gossypium* | ML 5004 10008 | 0.709343 | 0.000050 |
|  | | | | |  |  |  |  |

**Table S1.** ^87^Sr/^86^Sr of botanical material from the site of Mleiha. *Corrected ratio + standard bracketing.

| Sample ID | HCL leach | | HF leach | | Residual Textile | |
| --- | --- | --- | --- | --- | --- | --- |
|  | ^87^Sr/^86^Sr* | 2σ uncertainty | ^87^Sr/^86^Sr* | 2σ uncertainty | ^87^Sr/^86^Sr* | 2σ uncertainty |
| Sr 434 | 0.708643 | 0.000019 | 0.708893 | 0.000019 | 0.708836 | 0.000014 |
| Sr 435 | 0.708638 | 0.000019 | 0.709159 | 0.000019 | 0.711216 | 0.000014 |
| Sr 436 | 0.708621 | 0.000019 | 0.708714 | 0.000019 | 0.710391 | 0.000017 |
| Sr 437 | 0.708650 | 0.000019 | 0.713272 | 0.000019 | 0.714131 | 0.000029 |

**Table S2.** ^87^Sr/^86^Sr of botanical material from the site of Mleiha. *Corrected ratio + standard bracketing.

| **target #** | **UF** | **sample type** | **species** | **age** | **error** | **Calibrated range Cal CE (95.4% confidence interval)** | |
| --- | --- | --- | --- | --- | --- | --- | --- |
|  |  |  |  |  |  | **from** | **to** |
| AA90698 | - | seed | sp. indet | 1863 | 20 | 83 | 220 |
| SacA 24648 | UF5020 | seed | *Vitis vinifera* | 1820 | 30 | 90 | 321 |
| SacA 24649 | UF5009 | seed | *Vitis vinifera* | 1850 | 30 | 85 | 235 |
| SacA 24650 | UF5031 | seed | *Vitis vinifera* | 1835 | 30 | 86 | 246 |
| ECHo 1841 | UF 5020 | textile | *Gossypium* sp. | 1845 | 25 | 87 | 238 |
| ECHo 1842 | UF 5004 | textile | *Gossypium* sp. | 1855 | 25 | 85 | 230 |
| R_Combine |  |  |  |  |  | 127 | 224 |

**Table S3.** Radiocarbon dates of seeds and textiles from Mleiha.

| **Remains** | **Room** | **Function** | **Excavation unit** | **Sample number** | **Twisting direction (textile)** | **Weave (textile)** |
| --- | --- | --- | --- | --- | --- | --- |
| Textile | P1500 | Administrative? | UF 5004 | 10184.2 | Z/Z | Tabby |
| Textile | P1500 | Administrative? | UF 5004 | 10008.1 | Z/Z | Tabby |
| Textile | P1524 | Courtyard | UF 5020 | 10135.1 | Z/Z | Tabby |
| Textile | P1524 | Courtyard | UF 5027 | 10354 | Z/Z |  |
| Textile | P1524 | Courtyard | UF 5035A | 10280.1 | Z5/S1 |  |
| Textile | P1529 | Storage room | UF 5031 | 10253.5 | S/X (potentially mixed with linen/F.L) |  |
| Seed | P1500 | Administrative? | UF 5004 | 10008 (I) |  |  |
| Seed | P1500 | Administrative? | UF 5004 | 10008 (II) |  |  |
| Seed | P1500 | Administrative? | UF 5004 | 10008 (III) |  |  |
| Raw fibre cluster | P1500 | Administrative? | UF 5004 | 10008 |  |  |

**Table S4.** Details of archaeological cotton samples.

| **Place** | **Region** | **Country** | **Classic Mleiha pottery analysis** | **Petrographic analysis of Mleiha pottery sherds** | **Other archaeological material in Mleiha** | **Archaeobotanical evidence from Mleiha** | **Cotton seed** | **Cotton textile** | **Others** | **Texts: Commercial ports/sites with cotton products** | **Texts: Production place** | **Texts: Other information** |
| --- | --- | --- | --- | --- | --- | --- | --- | --- | --- | --- | --- | --- |
| **Tillya-tepe** |  | Afghanistan |  |  |  |  |  | 1st c. CE (Allsen 1997: 68, quoted in Bulliet 2009: 6, n. 10) ^12^ |  |  |  |  |
| **Tylos** |  | Bahrain |  |  |  |  |  |  |  |  | Observation at the time of Alexander the Great’s military expedition to India (325-324 BCE) quoted by Theophrastus (*Historia Plantarum* IV.7)^13^ ‘‘the island also produces the ‘wool-bearing’ tree in abundance. This has a leaf like that of the vine but small, and bears no fruit; but the vessel in which the ‘wool’ is contained is as large as a spring apple, and closed, but when it is ripe, it unfolds and puts forth the ‘wool’ of which they weave their fabrics, some of which are cheap and some very expensive’ (Amigues 2010: 159) |  |
| **Qal'at al-Bahrain** |  | Bahrain |  |  |  |  |  | Several cotton textiles in a bathtub cofﬁn, 600-400 BCE ^14^. |  |  |  |  |
| **Wari-Bateshwar** |  | Bangladesh |  |  |  |  | 400-100 BCE ^15^ |  |  |  |  |  |
| **Karadong** | Taklamakan south | China |  |  |  |  |  | Printed tabby, probably imported, 3rd c. CE (Desrosiers, comm. pers.) |  |  |  |  |
| **Loulan** | Xinjiang, Taklamakan Northeast | China |  |  |  |  |  | 2nd-3rd c. CE ^16,17^ |  |  |  |  |
| **Astana** | Xinjiang, Taklamakan north | China |  |  |  |  | 3rd-9th c. CE ^18^ |  |  |  |  |  |
|  | Mareotis | Egypt | Amphora type AE3 in building CW; ^5^ |  |  |  |  |  |  |  |  |  |
| **Qasr Ibrim** | Lower Nubia | Egypt |  |  |  |  | Charred and desiccated seeds, complete bolls, capsules, 25 BCE-1812 CE (see synthesis in Bouchaud et al. 2018)^19^ | Desiccated textiles, 200-1200 CE (see synthesis in Yvanez&Wozniak 2019) ^20^ |  |  |  |  |
| **Karanog** | Lower Nubia | Egypt |  |  |  |  |  | Desiccated textiles, 100-300 CE ^21^ |  |  |  |  |
| **Ballana** | Lower Nubia | Egypt |  |  |  |  |  | Desiccated textiles, 50-350 CE ^22^ |  |  |  |  |
| **Qustul** | Lower Nubia | Egypt |  |  |  |  |  | Desiccated textiles, 50-350 CE ^22^ |  |  |  |  |
| **Kalabsha (Wadi Qitna)** | Lower Nubia | Egypt |  |  |  |  |  | Desiccated shrouds, 3th-5th CE ^23^ |  |  |  |  |
| **Kellis (Ismant el-Kharab)** | Dakhleh oasis | Egypt |  |  |  |  | Seeds, bolls, empty capsules, 2nd-4th CE ^24,25^ | Desiccated textiles (tabby), 2nd-4th CE ^26^ |  |  | Two sales receipts dated to 237 and 257 CE referring to cotton harvests (*O. Kellis* 68, 69, Worp and Hope 2004) + *Kellis Agricultural Account Book* in 360-370 CE (Bagnall 1997)^27^ |  |
| **Trimithis (Amheida)** | Kharga oasis | Egypt |  |  |  |  | Charred and desiccated seeds, 3rd c. CE ^28^ | Desiccated textiles, 3rd c. CE ^29^ |  |  |  |  |
| **Kysis (Douch)** | Kharga oasis | Egypt |  |  |  |  |  |  |  |  | Land registry (*P.land.* 7.142, Wagner 1987; in Bagnall 2008 ^30^ |  |
| **Qasr Sumayra** | Kharga oasis north | Egypt |  |  |  |  | Desiccated seeds in mudbricks, 1st-4th c. CE ^19^ |  |  |  |  |  |
| **Oxyrhynchus** | Nile valley | Egypt |  |  |  |  |  |  |  |  |  | Private letter mentionning "your mother made for you the cotton tunic", 2nd-3rd c. CE (*P.Oxy*. 59.3991, Bagnall and Cribiore 2006: 355-356) ^31^ |
| **Psobthis** | Bahariya oasis | Egypt |  |  |  |  |  |  |  |  |  | Private letter mentionning transport of cotton tunic from Psobthis to Oxyrhynchus, 2nd-3rd c. CE (*SB*.6.9025, Bagnall 2008)^30^ |
| **Berenike** | Red Sea coast | Egypt |  |  |  |  |  | Desiccated sails fragments, 1st-5th c. CE ^32–35^ |  |  |  |  |
| **Myos Hormos** | Red Sea coast | Egypt |  |  |  |  |  | Desiccated textiles, late 1st-early 2nd c. CE ^35–38^ |  |  |  |  |
| **Tebtynis (Umm el-Breigât)** | Fayum | Egypt |  |  |  |  |  | 1 desiccated textile, 2nd half 3rd c. CE ^39^ |  |  |  |  |
|  | Lower Nubia, Upper Egypt, Delta | Egypt and Nubia |  |  | Brown Ware with Chalky Grits, Lamps in building CW;^5^ |  |  |  |  |  |  |  |
| **Doghlauri / Dedoplis Gora / Aradetis Gora** |  | Georgia |  |  |  |  |  | mineralised or charred fibres among non-palynomorph elements, 83 CE. ^40^ |  |  |  |  |
| **Nevasa** | Maharashtra | India | Building H & CW; ^5,6^ | ^41^ |  |  |  |  |  |  |  |  |
| **Tagara (Ter)** | Maharashtra | India | Building H; ^6^ | ^41^ |  |  |  |  |  | *PME* 51:17.13 ^42^"from Tagara large quantities of cloth of ordinary quality, all kinds of cotton garments..." |  |  |
| **Nasik** | Maharashtra | India | Building H; ^6^ | ^41^ |  |  |  |  |  |  |  |  |
| **Junnar** | Maharashtra | India | Building H; ^6^ | ^41^ |  |  |  |  |  |  |  |  |
| **Paunar** | Maharashtra | India | Building H; ^6^ |  |  |  |  |  |  |  |  |  |
| **Bhokardan** | Maharashtra | India | Building H; ^6^ |  |  |  |  |  |  |  |  |  |
| **Prakasha** | Maharashtra | India | Building H; ^6^ |  |  |  |  |  |  |  |  |  |
| **Dwarka** | Gujarat | India | Building H; ^6^ | ^41^ |  |  |  |  |  |  |  |  |
| **Bet Dwarka island** | Gujarat | India | Building H; ^6^ |  |  |  |  |  |  |  |  |  |
| **Padri** | Gujarat | India | Building H; ^6^ | ^41^ |  |  |  |  |  |  |  |  |
| **Prabhas Patan (Somnath)** | Gujarat | India | Building H; ^6^ | ^41^ |  |  |  |  |  |  |  |  |
| **Navdatoli** | Madhya Pradesh | India | Building CW; ^5^ |  |  |  |  |  |  |  |  |  |
| **Piklihal** | Karnataka | India |  |  |  |  |  |  | Impression on pottery, 3rd c. BC ^43^ |  |  |  |
| **Bairat** | Rajasthan | India |  |  |  |  |  | Ghosh 1990: 334-335; quoted in Wild&Wild 2014b ^44^ |  |  |  |  |
| **Rairh** | Rajasthan | India |  |  |  |  |  | Ghosh 1990: 334-335; quoted in Wild&Wild 2014b ^44^ |  |  |  |  |
| **Barygaza** | Gujarat | India |  |  |  |  |  |  |  | *PME* 49:16.29-30 ^42^ "Barygaza exported cotton cloth of all kinds in addition to silk" |  |  |
| **Ozênê** | Madhya Pradesh | India |  |  |  |  |  |  |  |  | *PME* 48:16.14-16 ^42^ "There is in this region [sc. of Barygaza] towards the east a city called Ozênê, the former seat of the royal court, from which everything that contributes to the region's prosperity, including what contributes to trade with us, is brought down to Baragiza: onyx; agate (?); Indian garments of cotton..." |  |
| **Poduke (Arikamedu)** | Tamil Nadu | India |  |  |  |  |  |  |  | *PME* quoted by Seland 2016^45^ as "likely centre also for the processing and redistribution of cotton textiles from the South Indian hinterland." |  |  |
| **Argaru** | Tamil Nadu | India |  |  |  |  |  |  |  | *PME* 59: 20.2-3 ^42^ "It exported cotton garments called Argaritides |  |  |
| **Masalia (Masulipatam)** | Andhra Pradesh | India |  |  |  |  |  |  |  |  | *PME* 62: 20.21-22 ^42^ "a great many cotton garments are produced here" |  |
| **Gangês (Tâmralıpti?=Tamluk)** | Purba Medinipur | India |  |  |  |  |  |  |  | *PME* 63:21.5-6 ^42^ "On it is a port of trade with the same name as the river, Gangês, through wich are shipped out malabathron, Gangetic nard, pearls, and cotton garments of the very finest quality, the so-called Gangetic." |  |  |
| **Abêria** | Between Barygaza and Ozênê | India |  |  |  |  |  |  |  |  | *PME* 41:14.8 ^42^ "The region, very fertile, produces grain, rice, sesame oil, ghee, cotton, and the Indian cloths made from it, thos of ordinary quality" |  |
|  | Gujarat? | India |  |  |  | Rice (*Oryza sativa*) in building H ^7^ |  |  |  |  |  |  |
|  | Kerala | India |  |  |  | Pepper (*Piper nigrum*) and rice (*Oryza sativa*) (?) in building H ^7^ |  |  |  |  |  |  |
| **Ajanta** |  | India |  |  |  |  |  |  | 1st c. BCE-AD 480 Painting showing cotton preparation ^46^ |  |  |  |
| **Ufalda** | Uttaranchal | India |  |  |  |  | 0-600 CE ^47^ |  |  |  |  |  |
| **Sanghol** | Punjab | India |  |  |  |  | 1st-3rd c. CE ^48^ |  |  |  |  |  |
| **Charda** |  | India |  |  |  |  | 200-100 BCE (Chandala 2002 quoted in Fuller 2008) ^47^ |  |  |  |  |  |
| **Kodumanal** | Tamil Nadu | India |  |  |  |  | 300 BCE-300 CE ^49^ |  |  |  |  |  |
| **Perur** | Tamil Nadu | India |  |  |  |  | 300 BCE-300 CE ^49^ |  |  |  |  |  |
| **Mangudi** | Tamil Nadu | India |  |  |  |  | 300 BCE-300 CE ^49^ |  |  |  |  |  |
| **Kausambi** | Uttar Pradesh | India |  |  |  |  | 550-250 BCE (Chanchala 1995, quoted in Fuller 2008) ^47^ |  |  |  |  |  |
| **Hulaskhera** | Uttar Pradesh | India |  |  |  |  | 600 BCE-250 CE (Chanchala 1992, quoted in Fuller 2008) ^47^ |  |  |  |  |  |
| **Sarethi** | Uttar Pradesh | India |  |  |  |  | 3 charred cotton seeds, 100 BCE-300 CE ^50^ |  |  |  |  |  |
| **Paithan** | Maharashtra | India |  |  |  |  | from 300 BCE to 700 CE ^51^ |  |  |  |  |  |
|  |  | India/Africa |  |  | Ivory; ^5,6^ |  |  |  |  |  |  |  |
| **Kirkouk** |  | Iraq |  |  | Bitumen in building CW; ^52^ |  |  |  |  |  |  |  |
| **Kifri** |  | Iraq |  |  | Bitumen in building CW; ^52^ |  |  |  |  |  |  |  |
| **Hit** |  | Iraq |  |  | Bitumen in dwelling DA; ^52^ |  |  |  |  |  |  |  |
| **Abu el-Jir** |  | Iraq |  |  | Bitumen in dwelling DA; ^52^ |  |  |  |  |  |  |  |
| **Minab?** | Hormozgan | Iran | Fine Orange Painted Ware in buildings H & CW; ^5,6^ |  |  |  |  |  |  |  |  |  |
| **Arjan** | Fars/Khuzestan | Iran |  |  |  |  |  | 12 cotton fabrics, adorned with elaborated fringes and golden rosettes in one tomb of a member of the merchant aristocracy (650-575 BC) ^53^ |  |  |  |  |
| **At-Tar** |  | Iraq |  |  |  |  |  | Fujii 1987 quoted in Quillien 2019 ^53^ |  |  |  |  |
| **Uruk** |  | Iraq |  |  |  |  |  | Fragment of cotton fabric in one burial jar in one residential area (7th-6th c. BC ?) ^53^ |  |  |  | Kidinnû to make the garments of the gods for rituals and sending of fabrics (605-486 BCE) + temple archive of the Bit-Res in which a textile "karpasu" for the garments of the gods is quoted (253 BCE) ^53^ |
| **Nineveh** |  | Iraq |  |  |  |  |  |  |  |  | Cultivation of trees bearing whool in the gardens of the royal palace and harvesting of fibres to make clothes in the Sennacherib royal inscription (704-681 BCE) ^53^ |  |
| **Sippar** |  | Iraq |  |  |  |  |  |  |  |  |  | Several mentions of kidinnû (cotton) in cuneiform sources from 9th to 6th c. BCE about dedicating and manufacturing garments' gods of the Ebabbar temple as well as distribution from the temple and payment as rent from merchants ^53^ |
| **Babylone** |  | Iraq |  |  |  |  |  |  |  |  | Unique mention of kidinnû at Babylon levied as tax for the temple of Nergal in private archive (501 BCE) ^53^ | Private archive in which kid-ni-tu is mentionned in a dowry (281 BCE) ^53^ |
| **Kalhu (Nimrud)** |  | Iraq |  |  |  |  |  | One cotton fabric among several linen textiles. Fine fabric, no dye, golden decorations in Neo-Assyrian tomb of royal women (2nd half of the 8th c. BC) ^53^ |  |  |  |  |
| **Jason's tomb (Jerusalem)** |  | Israel |  |  |  |  |  | Cotton net, 210 cal. CE ^54,55^ |  |  |  |  |
| **Qumran** |  | Israel |  |  |  |  |  | 100 BCE-68 CE ^56^ |  |  |  |  |
| **Khirbet Qazone** |  | Jordan |  |  |  |  |  | 1st-3rd c. CE ^57^ |  |  |  |  |
| **Aila** |  | Jordan |  |  |  |  | charred seeds, 2nd-mid 3rd c. CE ^58^ |  |  |  |  |  |
| **Jarma** |  | Lybia |  |  |  |  | 2nd-6th c. CE ^59–61^ |  |  |  |  |  |
| **Shaibkan Dheri?** | Sardheri | Pakistan | Black storage jars in building H; ^6^ |  |  |  |  |  |  |  |  |  |
|  | Sind? | Pakistan |  |  |  | Rice (*Oryza sativa*) in building H ^7^ |  |  |  |  |  |  |
| **Hund** | Peshwar | Pakistan |  |  |  |  | 200 BCE-1400 CE ^47^ |  |  |  |  |  |
| **Hegra (Madâ'in Sâlih)** |  | Saudi Arabia |  |  |  |  | Charred seeds in habitats, 85-416 cal CE ^19,62^ | Desiccated fabrics in Nabataean tomb, cal. 49 BCE-227 CE ^19,62^ |  |  |  |  |
| **Jetavana** | Anuradhapura | Sri Lanka |  |  |  |  |  | 3rd c. CE ^43^ |  |  |  |  |
| **Kirinda** | Southern coast | Sri Lanka |  |  |  |  | cal.end 3rd-4th c. CE ^63^ |  |  |  |  |  |
| **Mouweis** | Central Sudan | Sudan |  |  |  |  | 86-421 cal. CE ^19^ |  |  |  |  |  |
| **Hamadab** | Central Sudan | Sudan |  |  |  |  | 1st-3rd c. CE ^64^ |  |  |  |  |  |
| **Meroe** | Central Sudan | Sudan |  |  |  |  |  | Desiccated textiles in tumbs, 0-150 CE ^21,65,66^ |  |  |  |  |
|  | Lower Nubia | Sudan |  |  |  |  |  |  |  |  | Aizana inscription "I burned their settlements, both of walled houses and straw huts, and my people captured their corn, their bronze and iron and dried meat (?) and destroyed the figures of their gods and their provisions of corn and cotton, and east them into the Seda-river" ^67^ |  |
| **Sai** | Upper Nubia | Sudan |  |  |  |  |  | Desiccated textiles in tumbs, 150-300 CE ^68^ |  |  |  |  |
| **Aksha** | Lower Nubia | Sudan |  |  |  |  |  | Desiccated textiles in tumbs, 50 BCE-150 CE ^69^ |  |  |  |  |
| **Semna South** | Lower Nubia | Sudan |  |  |  |  |  | Desiccated textiles in tumbs, 200-400 CE ^20,70^ |  |  |  |  |
| **Dura Europos** |  | Syria |  |  |  |  |  | Desiccated textile, 3rd c. CE ^71^ |  |  |  |  |
| **Palmyra** |  | Syria |  |  |  |  |  | Desiccated textiles, 1st-2nd c. CE ^72,73^ |  |  |  |  |
| **Khao Sam Kaeo** | Chumphon province | Thailand |  |  |  |  | Charred funicular cap, 4th-1st c. BCE ^74^ |  |  |  |  |  |
| **Ban Don Ta Phet** |  | Thailand |  |  |  |  |  | Textile, 4th c. BCE ^75,76^ |  |  |  |  |
|  |  | Vietnam |  |  |  |  |  |  |  |  | End 3rd c. CE ^77^: "Dans le district d'Anding de [la commanderie de] Jiaozhi (actuel Vietnam du Nord), il existe un arbre appelé mumiam ; il est grand (ou "mesure une toise" soit environ 2,5 mètres). Son fruit ressemble à un godet à alcool ; il contient de la bourre (mian, bourre de soie) comme celle que produit le vers à soie. on peut aussi en faire un tissu (hu, tissu de chanvre, par opposition à ho, tissu de soie) appelé "haixie" ou parfois "maobu" (tissu de laine ou de poil" |  |
|  | Southern Mesopotamia |  | Glazed ware in buildings H & CW; ^5,6^ |  |  |  |  |  |  |  |  |  |
|  | Mesopotamia |  |  |  | Glass vessels in buildings H & CW; ^5,6^ |  |  |  |  |  |  |  |
| **Skhalta** | Caucasus south, Tetritskaro district |  |  |  |  |  |  | 4th-3rd c. BCE^40^ |  |  |  |  |

**Table S5.** Sites with evidence of cotton, in the form of textual references and/or presence of archaeological cotton seeds/textiles, dating from the first mill. BCE to the 3^rd^ c. CE. PME = Periplus Maris Erythraei, 1^st^ c. CE. Place names in parentheses are modern names.

| **Citation** | **Period** | **Age range** | **Medium*** | **Individual** | **Site** | **Country** | **Tooth** | **87Sr/86Sr** | **Taxon** | **Region** | **Context** |
| --- | --- | --- | --- | --- | --- | --- | --- | --- | --- | --- | --- |
| Lagad et al 2017^78^ | Modern Rice | | Modern Rice | 1 | Uttar Pradesh | India |  | 0.717670 | *Oryza Sativa* |  |  |
| Lagad et al 2017 | Modern Rice | | Modern Rice | 2 | Uttar Pradesh | India |  | 0.730554 | *Oryza Sativa* |  |  |
| Lagad et al 2017 | Modern Rice | | Modern Rice | 3 | Uttar Pradesh | India |  | 0.731270 | *Oryza Sativa* |  |  |
| Lagad et al 2017 | Modern Rice | | Modern Rice | 4 | Uttar Pradesh | India |  | 0.728748 | *Oryza Sativa* |  |  |
| Lagad et al 2017 | Modern Rice | | Modern Rice | 5 | Uttar Pradesh | India |  | 0.734477 | *Oryza Sativa* |  |  |
| Lagad et al 2017 | Modern Rice | | Modern Rice | 6 | Uttar Pradesh | India |  | 0.720616 | *Oryza Sativa* |  |  |
| Lagad et al 2017 | Modern Rice | | Modern Rice | 7 | Uttar Pradesh | India |  | 0.726931 | *Oryza Sativa* |  |  |
| Lagad et al 2017 | Modern Rice | | Modern Rice | 8 | Uttar Pradesh | India |  | 0.724690 | *Oryza Sativa* |  |  |
| Lagad et al 2017 | Modern Rice | | Modern Rice | 9 | Uttar Pradesh | India |  | 0.720690 | *Oryza Sativa* |  |  |
| Lagad et al 2017 | Modern Rice | | Modern Rice | 10 | Uttar Pradesh | India |  | 0.719993 | *Oryza Sativa* |  |  |
| Lagad et al 2017 | Modern Rice | | Modern Rice | 27 | Uttar Pradesh | India |  | 0.717254 | *Oryza Sativa* |  |  |
| Lagad et al 2017 | Modern Rice | | Modern Rice | 28 | Uttar Pradesh | India |  | 0.724692 | *Oryza Sativa* |  |  |
| Lagad et al 2017 | Modern Rice | | Modern Rice | 29 | Uttar Pradesh | India |  | 0.717546 | *Oryza Sativa* |  |  |
| Lagad et al 2017 | Modern Rice | | Modern Rice | 30 | Uttar Pradesh | India |  | 0.716049 | *Oryza Sativa* |  |  |
| Lagad et al 2017 | Modern Rice | | Modern Rice | 31 | Uttar Pradesh | India |  | 0.719312 | *Oryza Sativa* |  |  |
| Lagad et al 2017 | Modern Rice | | Modern Rice | 32 | Uttar Pradesh | India |  | 0.728840 | *Oryza Sativa* |  |  |
| Lagad et al 2017 | Modern Rice | | Modern Rice | 33 | Uttar Pradesh | India |  | 0.726309 | *Oryza Sativa* |  |  |
| Lagad et al 2017 | Modern Rice | | Modern Rice | 34 | Uttar Pradesh | India |  | 0.723425 | *Oryza Sativa* |  |  |
| Lagad et al 2017 | Modern Rice | | Modern Rice | 35 | Uttar Pradesh | India |  | 0.723425 | *Oryza Sativa* |  |  |
| Lagad et al 2017 | Modern Rice | | Modern Rice | 36 | Uttar Pradesh | India |  | 0.733900 | *Oryza Sativa* |  |  |
| Lagad et al 2017 | Modern Rice | | Modern Rice | 37 | Uttar Pradesh | India |  | 0.722521 | *Oryza Sativa* |  |  |
| Lagad et al 2017 | Modern Rice | | Modern Rice | 38 | Uttar Pradesh | India |  | 0.725733 | *Oryza Sativa* |  |  |
| Lagad et al 2017 | Modern Rice | | Modern Rice | 39 | Uttar Pradesh | India |  | 0.717591 | *Oryza Sativa* |  |  |
| Lagad et al 2017 | Modern Rice | | Modern Rice | 40 | Uttar Pradesh | India |  | 0.717390 | *Oryza Sativa* |  |  |
| Lagad et al 2017 | Modern Rice | | Modern Rice | 11 | Uttarakhand | India |  | 0.713362 | *Oryza Sativa* |  |  |
| Lagad et al 2017 | Modern Rice | | Modern Rice | 12 | Uttarakhand | India |  | 0.711428 | *Oryza Sativa* |  |  |
| Lagad et al 2017 | Modern Rice | | Modern Rice | 13 | Uttarakhand | India |  | 0.712935 | *Oryza Sativa* |  |  |
| Lagad et al 2017 | Modern Rice | | Modern Rice | 14 | Uttarakhand | India |  | 0.720543 | *Oryza Sativa* |  |  |
| Lagad et al 2017 | Modern Rice | | Modern Rice | 15 | Uttarakhand | India |  | 0.721605 | *Oryza Sativa* |  |  |
| Lagad et al 2017 | Modern Rice | | Modern Rice | 41 | Uttarakhand | India |  | 0.713049 | *Oryza Sativa* |  |  |
| Lagad et al 2017 | Modern Rice | | Modern Rice | 42 | Uttarakhand | India |  | 0.717827 | *Oryza Sativa* |  |  |
| Valentine 2013^79^ | first half of the 2nd millennium BC | | Human tooth enamel | SYC-1 | Sanauli | India | RM1 | 0.72078 | *Homo* |  | YC1/2hearth |
| Valentine 2013 | first half of the 2nd millennium BC | | Human tooth enamel | SYC-2 | Sanauli | India | RM2 | 0.72042 | *Homo* |  | YC1/2hearth |
| Valentine 2013 | first half of the 2nd millennium BC | | Human tooth enamel | SU3-1/21 | Sanauli | India | RM1/2 | 0.72064 | *Homo* |  | unknown3 |
| Valentine 2013 | first half of the 2nd millennium BC | | Human tooth enamel | S20-p4 | Sanauli | India | LP4 | 0.72714 | *Homo* |  | 20 |
| Valentine 2013 | first half of the 2nd millennium BC | | Human tooth enamel | S20-3 | Sanauli | India | LM3 | 0.72213 | *Homo* |  | 20 |
| Valentine 2013 | first half of the 2nd millennium BC | | Human tooth enamel | S22-1 | Sanauli | India | RM1 | 0.71709 | *Homo* |  | 22 |
| Valentine 2013 | first half of the 2nd millennium BC | | Human tooth enamel | S22-2 | Sanauli | India | RM2 | 0.71693 | *Homo* |  | 22 |
| Valentine 2013 | first half of the 2nd millennium BC | | Human tooth enamel | S25-1 | Sanauli | India | LM1 | 0.71938 | *Homo* |  | 25 |
| Valentine 2013 | first half of the 2nd millennium BC | | Human tooth enamel | S25-2 | Sanauli | India | LM2 | 0.71957 | *Homo* |  | 25 |
| Valentine 2013 | first half of the 2nd millennium BC | | Human tooth enamel | S25-3 | Sanauli | India | LM3 | 0.71984 | *Homo* |  | 25 |
| Valentine 2013 | first half of the 2nd millennium BC | | Human tooth enamel | S29-p3 | Sanauli | India | LP3 | 0.71962 | *Homo* |  | 29 |
| Valentine 2013 | first half of the 2nd millennium BC | | Human tooth enamel | S34-1/21 | Sanauli | India | RM1/2 | 0.71984 | *Homo* |  | 34 |
| Valentine 2013 | first half of the 2nd millennium BC | | Human tooth enamel | S36-d22 | Sanauli | India | LdM2 | 0.71881 | *Homo* |  | 36 |
| Valentine 2013 | first half of the 2nd millennium BC | | Human tooth enamel | S36-1 | Sanauli | India | RM1 | 0.71876 | *Homo* |  | 36 |
| Valentine 2013 | first half of the 2nd millennium BC | | Human tooth enamel | S37-1 | Sanauli | India | LM1 | 0.72182 | *Homo* |  | 37 |
| Valentine 2013 | first half of the 2nd millennium BC | | Human tooth enamel | S37-2 | Sanauli | India | LM2 | 0.72216 | *Homo* |  | 37 |
| Valentine 2013 | first half of the 2nd millennium BC | | Human tooth enamel | S37-3 | Sanauli | India | LM3 | 0.72213 | *Homo* |  | 37 |
| Valentine 2013 | first half of the 2nd millennium BC | | Human tooth enamel | S38-1 | Sanauli | India | RM1 | 0.71925 | *Homo* |  | 38 |
| Valentine 2013 | first half of the 2nd millennium BC | | Human tooth enamel | S38-2 | Sanauli | India | RM2 | 0.71906 | *Homo* |  | 38 |
| Valentine 2013 | first half of the 2nd millennium BC | | Human tooth enamel | S44A-1 | Sanauli | India | RM1 | 0.71923 | *Homo* |  | 44A |
| Valentine 2013 | first half of the 2nd millennium BC | | Human tooth enamel | S44A-2 | Sanauli | India | RM2 | 0.71900 | *Homo* |  | 44A |
| Valentine 2013 | first half of the 2nd millennium BC | | Human tooth enamel | S44A-3 | Sanauli | India | RM3 | 0.71891 | *Homo* |  | 44A |
| Valentine 2013 | first half of the 2nd millennium BC | | Human tooth enamel | S44B-1 | Sanauli | India | LM1 | 0.72164 | *Homo* |  | 44B |
| Valentine 2013 | first half of the 2nd millennium BC | | Human tooth enamel | S44B-2 | Sanauli | India | LM2 | 0.72119 | *Homo* |  | 44B |
| Valentine 2013 | first half of the 2nd millennium BC | | Human tooth enamel | S44B-3 | Sanauli | India | LM3 | 0.72175 | *Homo* |  | 44B |
| Valentine 2013 | first half of the 2nd millennium BC | | Human tooth enamel | S44C-1 | Sanauli | India | RM1 | 0.71757 | *Homo* |  | 44C |
| Valentine 2013 | first half of the 2nd millennium BC | | Human tooth enamel | S44C-2 | Sanauli | India | RM2 | 0.71762 | *Homo* |  | 44C |
| Valentine 2013 | first half of the 2nd millennium BC | | Human tooth enamel | S49-2 | Sanauli | India | RM2 | 0.72101 | *Homo* |  | 49 |
| Valentine 2013 | first half of the 2nd millennium BC | | Human tooth enamel | S54-1 | Sanauli | India | LM1 | 0.71894 | *Homo* |  | 54 |
| Valentine 2013 | first half of the 2nd millennium BC | | Human tooth enamel | S54-2 | Sanauli | India | RM2 | 0.71807 | *Homo* |  | 54 |
| Valentine 2013 | first half of the 2nd millennium BC | | Human tooth enamel | S55-1/21 | Sanauli | India | LM1/2 | 0.71254 | *Homo* |  | 55 |
| Valentine 2013 | first half of the 2nd millennium BC | | Human tooth enamel | S58-i2 | Sanauli | India | LI2 | 0.71863 | *Homo* |  | 58 |
| Valentine 2013 | first half of the 2nd millennium BC | | Human tooth enamel | S61-1 | Sanauli | India | RM1 | 0.71866 | *Homo* |  | 61 |
| Valentine 2013 | first half of the 2nd millennium BC | | Human tooth enamel | S61-2 | Sanauli | India | RM2 | 0.71851 | *Homo* |  | 61 |
| Valentine 2013 | first half of the 2nd millennium BC | | Human tooth enamel | S61-3 | Sanauli | India | RM3 | 0.71895 | *Homo* |  | 61 |
| Valentine 2013 | first half of the 2nd millennium BC | | Human tooth enamel | S67-d22 | Sanauli | India | RdM2 | 0.72209 | *Homo* |  | 67 |
| Valentine 2013 | first half of the 2nd millennium BC | | Human tooth enamel | S69A-1 | Sanauli | India | LM1 | 0.71983 | *Homo* |  | 69A |
| Valentine 2013 | first half of the 2nd millennium BC | | Human tooth enamel | S69A-2 | Sanauli | India | LM2 | 0.71950 | *Homo* |  | 69A |
| Valentine 2013 | first half of the 2nd millennium BC | | Human tooth enamel | S69A-3 | Sanauli | India | LM3 | 0.71899 | *Homo* |  | 69A |
| Valentine 2013 | first half of the 2nd millennium BC | | Human tooth enamel | S69B-1 | Sanauli | India | LM1 | 0.71816 | *Homo* |  | 69B |
| Valentine 2013 | first half of the 2nd millennium BC | | Human tooth enamel | S69C-2 | Sanauli | India | LM2 | 0.71953 | *Homo* |  | 69C |
| Valentine 2013 | first half of the 2nd millennium BC | | Human tooth enamel | S69C-3 | Sanauli | India | LM3 | 0.71909 | *Homo* |  | 69C |
| Valentine 2013 | first half of the 2nd millennium BC | | Human tooth enamel | S71-1 | Sanauli | India | LM1 | 0.71974 | *Homo* |  | 71 |
| Valentine 2013 | first half of the 2nd millennium BC | | Human tooth enamel | S71-2 | Sanauli | India | LM2 | 0.71978 | *Homo* |  | 71 |
| Valentine 2013 | first half of the 2nd millennium BC | | Human tooth enamel | S74A-1 | Sanauli | India | LM1 | 0.71900 | *Homo* |  | 74A |
| Valentine 2013 | first half of the 2nd millennium BC | | Human tooth enamel | S74A-2 | Sanauli | India | LM2 | 0.71910 | *Homo* |  | 74A |
| Valentine 2013 | first half of the 2nd millennium BC | | Human tooth enamel | S74A-3 | Sanauli | India | LM3 | 0.72005 | *Homo* |  | 74A |
| Valentine 2013 | first half of the 2nd millennium BC | | Human tooth enamel | S74B-d22 | Sanauli | India | RdM2 S80-1 | 0.71893 | *Homo* |  | 74B |
| Valentine 2013 | first half of the 2nd millennium BC | | Human tooth enamel | 80 | Sanauli | India | 80 | 0.71940 | *Homo* |  | RM1 S80-2 |
| Valentine 2013 | first half of the 2nd millennium BC | | Human tooth enamel | RM2 | Sanauli | India |  | 0.71991 | *Homo* |  |  |
| Valentine 2013 | first half of the 2nd millennium BC | | Human tooth enamel | S80-3 | Sanauli | India | RM3 | 0.71875 | *Homo* |  | 80 |
| Valentine 2013 | first half of the 2nd millennium BC | | Human tooth enamel | S85-1 | Sanauli | India | LM1 | 0.72133 | *Homo* |  | 85 |
| Valentine 2013 | first half of the 2nd millennium BC | | Human tooth enamel | S85-2 | Sanauli | India | LM2 | 0.72069 | *Homo* |  | 85 |
| Valentine 2013 | first half of the 2nd millennium BC | | Human tooth enamel | S93-1 | Sanauli | India | LM1 | 0.71953 | *Homo* |  | 93 |
| Valentine 2013 | first half of the 2nd millennium BC | | Human tooth enamel | S95-d22 | Sanauli | India | RdM2 | 0.71933 | *Homo* |  | 95 |
| Valentine 2013 | first half of the 2nd millennium BC | | Human tooth enamel | S97-1 | Sanauli | India | LM1 | 0.71905 | *Homo* |  | 97 |
| Valentine 2013 | first half of the 2nd millennium BC | | Human tooth enamel | S97-2 | Sanauli | India | LM2 | 0.71946 | *Homo* |  | 97 |
| Valentine 2013 | first half of the 2nd millennium BC | | Human tooth enamel | S97-3 | Sanauli | India | LM3 | 0.7194 | *Homo* |  | 97 |
| Valentine 2013 | first half of the 2nd millennium BC | | Human tooth enamel | S98-1 | Sanauli | India | LM1 | 0.72021 | *Homo* |  | 98 |
| Valentine 2013 | first half of the 2nd millennium BC | | Human tooth enamel | S98-2 | Sanauli | India | LM2 | 0.72048 | *Homo* |  | 98 |
| Valentine 2013 | first half of the 2nd millennium BC | | Human tooth enamel | S98-3 | Sanauli | India | LM3 | 0.72137 | *Homo* |  | 98 |
| Valentine 2013 | first half of the 2nd millennium BC | | Human tooth enamel | S99-d22 | Sanauli | India | LdM2 | 0.7199 | *Homo* |  | 99 |
| Valentine 2013 | first half of the 2nd millennium BC | | Human tooth enamel | S99-1 | Sanauli | India | LM1 | 0.72008 | *Homo* |  | 99 |
| Valentine 2013 | first half of the 2nd millennium BC | | Human tooth enamel | S112-1 | Sanauli | India | LM1 | 0.71846 | *Homo* |  | 112 |
| Valentine 2013 | first half of the 2nd millennium BC | | Human tooth enamel | S112-2 | Sanauli | India | RM2 | 0.71858 | *Homo* |  | 112 |
| Valentine 2013 | first half of the 2nd millennium BC | | Human tooth enamel | S112-3 | Sanauli | India | LM3 | 0.71962 | *Homo* |  | 112 |
| Lagad et al 2017 | Modern Rice | | Modern Rice | 79 | New Delhi | India |  | 0.716736 | *Oryza Sativa* |  |  |
| Lagad et al 2017 | Modern Rice | | Modern Rice | 80 | New Delhi | India |  | 0.717627 | *Oryza Sativa* |  |  |
| Lagad et al 2017 | Modern Rice | | Modern Rice | 81 | New Delhi | India |  | 0.718005 | *Oryza Sativa* |  |  |
| Lagad et al 2017 | Modern Rice | | Modern Rice | 82 | Delhi | India |  | 0.717599 | *Oryza Sativa* |  |  |
| Vanentine et al 2015^80^ | Harappa Phase (2600–1900 BC) | | Human Tooth Enamel | F2-1 | Farmana | India | LM_1_ | 0.71529 | *Homo* |  |  |
| Vanentine et al 2015 | Harappa Phase (2600–1900 BC) | | Human Tooth Enamel | F2-2 | Farmana | India | LM_2_ | 0.71529 | *Homo* |  |  |
| Vanentine et al 2015 | Harappa Phase (2600–1900 BC) | | Human Tooth Enamel | F2-3 | Farmana | India | LM_3_ | 0.71541 | *Homo* |  |  |
| Vanentine et al 2015 | Harappa Phase (2600–1900 BC) | | Human Tooth Enamel | F6-1 | Farmana | India | LM_1_ | 0.71582 | *Homo* |  |  |
| Vanentine et al 2015 | Harappa Phase (2600–1900 BC) | | Human Tooth Enamel | F6-2 | Farmana | India | LM_2_ | 0.71584 | *Homo* |  |  |
| Vanentine et al 2015 | Harappa Phase (2600–1900 BC) | | Human Tooth Enamel | F6-3 | Farmana | India | LM_3_ | 0.71581 | *Homo* |  |  |
| Vanentine et al 2015 | Harappa Phase (2600–1900 BC) | | Human Tooth Enamel | F11-2 | Farmana | India | LM_2_ | 0.71581 | *Homo* |  |  |
| Vanentine et al 2015 | Harappa Phase (2600–1900 BC) | | Human Tooth Enamel | F11-3 | Farmana | India | LM_3_ | 0.71583 | *Homo* |  |  |
| Vanentine et al 2015 | Harappa Phase (2600–1900 BC) | | Human Tooth Enamel | F14-1 | Farmana | India | RM^1^ | 0.71551 | *Homo* |  |  |
| Vanentine et al 2015 | Harappa Phase (2600–1900 BC) | | Human Tooth Enamel | F14-2 | Farmana | India | LM^2^ | 0.71549 | *Homo* |  |  |
| Vanentine et al 2015 | Harappa Phase (2600–1900 BC) | | Human Tooth Enamel | F14-3 | Farmana | India | RM^3^ | 0.71560 | *Homo* |  |  |
| Vanentine et al 2015 | Harappa Phase (2600–1900 BC) | | Human Tooth Enamel | F18-1 | Farmana | India | LM^1^ | 0.71589 | *Homo* |  |  |
| Vanentine et al 2015 | Harappa Phase (2600–1900 BC) | | Human Tooth Enamel | F18-2 | Farmana | India | LM_2_ | 0.71587 | *Homo* |  |  |
| Vanentine et al 2015 | Harappa Phase (2600–1900 BC) | | Human Tooth Enamel | F18-3 | Farmana | India | RM_3_ | 0.71578 | *Homo* |  |  |
| Vanentine et al 2015 | Harappa Phase (2600–1900 BC) | | Human Tooth Enamel | F20-1 | Farmana | India | RM^1^ | 0.71572 | *Homo* |  |  |
| Vanentine et al 2015 | Harappa Phase (2600–1900 BC) | | Human Tooth Enamel | F20-3 | Farmana | India | RM^3^ | 0.71571 | *Homo* |  |  |
| Vanentine et al 2015 | Harappa Phase (2600–1900 BC) | | Human Tooth Enamel | F23-1 | Farmana | India | RM_1_ | 0.71592 | *Homo* |  |  |
| Vanentine et al 2015 | Harappa Phase (2600–1900 BC) | | Human Tooth Enamel | F23-2 | Farmana | India | RM_2_ | 0.71584 | *Homo* |  |  |
| Vanentine et al 2015 | Harappa Phase (2600–1900 BC) | | Human Tooth Enamel | F23-3 | Farmana | India | RM_3_ | 0.71579 | *Homo* |  |  |
| Vanentine et al 2015 | Harappa Phase (2600–1900 BC) | | Human Tooth Enamel | F26-2 | Farmana | India | RM^2^ | 0.71589 | *Homo* |  |  |
| Vanentine et al 2015 | Harappa Phase (2600–1900 BC) | | Human Tooth Enamel | F26-3 | Farmana | India | RM^3^ | 0.71600 | *Homo* |  |  |
| Vanentine et al 2015 | Harappa Phase (2600–1900 BC) | | Human Tooth Enamel | F41-1 | Farmana | India | RM^1^ | 0.71576 | *Homo* |  |  |
| Vanentine et al 2015 | Harappa Phase (2600–1900 BC) | | Human Tooth Enamel | F45-p3 | Farmana | India | RP^3^ | 0.71756 | *Homo* |  |  |
| Vanentine et al 2015 | Harappa Phase (2600–1900 BC) | | Human Tooth Enamel | F47-p4 | Farmana | India | RP^4^ | 0.71588 | *Homo* |  |  |
| Vanentine et al 2015 | Harappa Phase (2600–1900 BC) | | Human Tooth Enamel | F54-1 | Farmana | India | RM_1_ | 0.71620 | *Homo* |  |  |
| Vanentine et al 2015 | Harappa Phase (2600–1900 BC) | | Human Tooth Enamel | F58-2 | Farmana | India | RM_2_ | 0.71570 | *Homo* |  |  |
| Vanentine et al 2015 | Harappa Phase (2600–1900 BC) | | Human Tooth Enamel | F62-1 | Farmana | India | LM^1^ | 0.71572 | *Homo* |  |  |
| Vanentine et al 2015 | Harappa Phase (2600–1900 BC) | | Human Tooth Enamel | F62-3 | Farmana | India | LM^3^ | 0.71590 | *Homo* |  |  |
| Vanentine et al 2015 | Harappa Phase (2600–1900 BC) | | Human Tooth Enamel | F65-1 | Farmana | India | RM_1_ | 0.71578 | *Homo* |  |  |
| Vanentine et al 2015 | Harappa Phase (2600–1900 BC) | | Human Tooth Enamel | F65-2 | Farmana | India | RM_2_ | 0.71588 | *Homo* |  |  |
| Vanentine et al 2015 | Harappa Phase (2600–1900 BC) | | Human Tooth Enamel | F66-3 | Farmana | India | RM^3^ | 0.71704 | *Homo* |  |  |
| Vanentine et al 2015 | Harappa Phase (2600–1900 BC) | | Human Tooth Enamel | F67-2 | Farmana | India | LM^2^ | 0.71975 | *Homo* |  |  |
| Vanentine et al 2015 | Harappa Phase (2600–1900 BC) | | Human Tooth Enamel | F67-3 | Farmana | India | LM_3_ | 0.72038 | *Homo* |  |  |
| Lagad et al 2017 | Modern Rice |  | Modern Rice | 24 | Punjab | India |  | 0.715031 | *Oryza Sativa* |  |  |
| Lagad et al 2017 | Modern Rice |  | Modern Rice | 25 | Punjab | India |  | 0.715616 | *Oryza Sativa* |  |  |
| Lagad et al 2017 | Modern Rice |  | Modern Rice | 26 | Punjab | India |  | 0.717944 | *Oryza Sativa* |  |  |
| Lagad et al 2017 | Modern Rice |  | Modern Rice | 69 | Punjab | India |  | 0.715440 | *Oryza Sativa* |  |  |
| Lagad et al 2017 | Modern Rice |  | Modern Rice | 70 | Punjab | India |  | 0.715366 | *Oryza Sativa* |  |  |
| Lagad et al 2017 | Modern Rice |  | Modern Rice | 71 | Punjab | India |  | 0.717112 | *Oryza Sativa* |  |  |
| Lagad et al 2017 | Modern Rice |  | Modern Rice | 72 | Punjab | India |  | 0.716828 | *Oryza Sativa* |  |  |
| Lagad et al 2017 | Modern Rice |  | Modern Rice | 73 | Punjab | India |  | 0.718648 | *Oryza Sativa* |  |  |
| Lagad et al 2017 | Modern Rice |  | Modern Rice | 74 | Punjab | India |  | 0.718455 | *Oryza Sativa* |  |  |
| Lagad et al 2017 | Modern Rice |  | Modern Rice | 75 | Punjab | India |  | 0.719350 | *Oryza Sativa* |  |  |
| Lagad et al 2017 | Modern Rice |  | Modern Rice | 76 | Punjab | India |  | 0.716586 | *Oryza Sativa* |  |  |
| Lagad et al 2017 | Modern Rice |  | Modern Rice | 77 | Punjab | India |  | 0.721307 | *Oryza Sativa* |  |  |
| Lagad et al 2017 | Modern Rice |  | Modern Rice | 78 | Punjab | India |  | 0.717574 | *Oryza Sativa* |  |  |
| Lagad et al 2017 | Modern Rice |  | Modern Rice | 16 | Haryana | India |  | 0.716692 | *Oryza Sativa* |  |  |
| Lagad et al 2017 | Modern Rice |  | Modern Rice | 17 | Haryana | India |  | 0.715872 | *Oryza Sativa* |  |  |
| Lagad et al 2017 | Modern Rice |  | Modern Rice | 18 | Haryana | India |  | 0.715431 | *Oryza Sativa* |  |  |
| Lagad et al 2017 | Modern Rice |  | Modern Rice | 19 | Haryana | India |  | 0.716408 | *Oryza Sativa* |  |  |
| Lagad et al 2017 | Modern Rice |  | Modern Rice | 20 | Haryana | India |  | 0.716327 | *Oryza Sativa* |  |  |
| Lagad et al 2017 | Modern Rice |  | Modern Rice | 21 | Haryana | India |  | 0.716390 | *Oryza Sativa* |  |  |
| Lagad et al 2017 | Modern Rice |  | Modern Rice | 22 | Haryana | India |  | 0.718971 | *Oryza Sativa* |  |  |
| Lagad et al 2017 | Modern Rice |  | Modern Rice | 23 | Haryana | India |  | 0.717781 | *Oryza Sativa* |  |  |
| Lagad et al 2017 | Modern Rice |  | Modern Rice | 43 | Haryana | India |  | 0.716879 | *Oryza Sativa* |  |  |
| Lagad et al 2017 | Modern Rice |  | Modern Rice | 44 | Haryana | India |  | 0.717042 | *Oryza Sativa* |  |  |
| Lagad et al 2017 | Modern Rice |  | Modern Rice | 45 | Haryana | India |  | 0.716433 | *Oryza Sativa* |  |  |
| Lagad et al 2017 | Modern Rice |  | Modern Rice | 46 | Haryana | India |  | 0.717344 | *Oryza Sativa* |  |  |
| Lagad et al 2017 | Modern Rice |  | Modern Rice | 47 | Haryana | India |  | 0.716766 | *Oryza Sativa* |  |  |
| Lagad et al 2017 | Modern Rice |  | Modern Rice | 48 | Haryana | India |  | 0.717385 | *Oryza Sativa* |  |  |
| Lagad et al 2017 | Modern Rice |  | Modern Rice | 49 | Haryana | India |  | 0.717065 | *Oryza Sativa* |  |  |
| Lagad et al 2017 | Modern Rice |  | Modern Rice | 50 | Haryana | India |  | 0.717006 | *Oryza Sativa* |  |  |
| Lagad et al 2017 | Modern Rice |  | Modern Rice | 51 | Haryana | India |  | 0.715844 | *Oryza Sativa* |  |  |
| Lagad et al 2017 | Modern Rice |  | Modern Rice | 52 | Haryana | India |  | 0.719191 | *Oryza Sativa* |  |  |
| Lagad et al 2017 | Modern Rice |  | Modern Rice | 53 | Haryana | India |  | 0.718376 | *Oryza Sativa* |  |  |
| Lagad et al 2017 | Modern Rice |  | Modern Rice | 54 | Haryana | India |  | 0.717515 | *Oryza Sativa* |  |  |
| Lagad et al 2017 | Modern Rice |  | Modern Rice | 55 | Haryana | India |  | 0.719535 | *Oryza Sativa* |  |  |
| Lagad et al 2017 | Modern Rice |  | Modern Rice | 56 | Haryana | India |  | 0.716651 | *Oryza Sativa* |  |  |
| Lagad et al 2017 | Modern Rice |  | Modern Rice | 57 | Haryana | India |  | 0.713535 | *Oryza Sativa* |  |  |
| Lagad et al 2017 | Modern Rice |  | Modern Rice | 58 | Haryana | India |  | 0.718987 | *Oryza Sativa* |  |  |
| Lagad et al 2017 | Modern Rice |  | Modern Rice | 59 | Haryana | India |  | 0.718766 | *Oryza Sativa* |  |  |
| Lagad et al 2017 | Modern Rice |  | Modern Rice | 60 | Haryana | India |  | 0.721062 | *Oryza Sativa* |  |  |
| Lagad et al 2017 | Modern Rice |  | Modern Rice | 61 | Haryana | India |  | 0.717220 | *Oryza Sativa* |  |  |
| Lagad et al 2017 | Modern Rice |  | Modern Rice | 62 | Haryana | India |  | 0.725799 | *Oryza Sativa* |  |  |
| Lagad et al 2017 | Modern Rice |  | Modern Rice | 63 | Haryana | India |  | 0.719573 | *Oryza Sativa* |  |  |
| Lagad et al 2017 | Modern Rice |  | Modern Rice | 64 | Haryana | India |  | 0.733144 | *Oryza Sativa* |  |  |
| Lagad et al 2017 | Modern Rice |  | Modern Rice | 65 | Haryana | India |  | 0.716477 | *Oryza Sativa* |  |  |
| Lagad et al 2017 | Modern Rice |  | Modern Rice | 66 | Haryana | India |  | 0.719712 | *Oryza Sativa* |  |  |
| Lagad et al 2017 | Modern Rice |  | Modern Rice | 67 | Haryana | India |  | 0.729433 | *Oryza Sativa* |  |  |
| Lagad et al 2017 | Modern Rice |  | Modern Rice | 68 | Haryana | India |  | 0.718864 | *Oryza Sativa* |  |  |
| Valentine 2013^79^ | Harappa Phase (2600-1900 BC) | | Archaeological faunal tooth enamel | RS1 | Rakhigarhi | India |  | 0.71574 | Sus |  |  |
| Valentine 2013 | Harappa Phase (2600-1900 BC) | | Archaeological faunal tooth enamel | RS2 | Rakhigarhi | India |  | 0.71585 | Sus |  |  |
| Valentine 2013 | Harappa Phase (2600-1900 BC) | | Archaeological faunal tooth enamel | RS3 | Rakhigarhi | India |  | 0.71568 | Sus |  |  |
| Valentine 2013 | Harappa Phase (2600-1900 BC) | | Archaeological faunal tooth enamel | RS4 | Rakhigarhi | India |  | 0.71471 | Sus |  |  |
| Valentine 2013 | Harappa Phase (2600-1900 BC) | | Archaeological faunal tooth enamel | RS5 | Rakhigarhi | India |  | 0.71903 | Sus |  |  |
| Valentine 2013 | Harappa Phase (2600-1900 BC) | | Archaeological faunal tooth enamel | RS6 | Rakhigarhi | India |  | 0.71702 | Sus |  |  |
| Valentine 2013 | Harappa Phase (2600-1900 BC) | | Archaeological faunal tooth enamel | RS7 | Rakhigarhi | India |  | 0.71582 | Sus |  |  |
| Valentine 2013 | Harappa Phase (2600-1900 BC) | | Archaeological faunal tooth enamel | RS8 | Rakhigarhi | India |  | 0.71556 | Sus |  |  |
| Chase et al 2018^81^ | Modern |  | Faunal herbivore dung |  | Mainland | India |  | 0.7084 | cattle/buffalo | Gujarat, Western India |  |
| Chase et al 2018 | Modern |  | Faunal herbivore dung | | Mainland | India |  | 0.7095 | cattle/buffalo | Gujarat, Western India |  |
| Chase et al 2018 | Modern |  | Faunal herbivore dung | | Mainland | India |  | 0.7088 | cattle/buffalo | Gujarat, Western India |  |
| Chase et al 2018 | Modern |  | Faunal herbivore dung | | Mainland | India |  | 0.7103 | cattle/buffalo | Gujarat, Western India |  |
| Chase et al 2018 | Modern |  | Faunal herbivore dung | | Mainland | India |  | 0.7103 | cattle/buffalo | Gujarat, Western India |  |
| Chase et al 2018 | Modern |  | Faunal herbivore dung | | Mainland | India |  | 0.7105 | goat/sheep | Gujarat, Western India |  |
| Chase et al 2018 | Modern |  | Faunal herbivore dung | | Mainland | India |  | 0.7101 | goat/sheep | Gujarat, Western India |  |
| Chase et al 2018 | Modern |  | Faunal herbivore dung | | Mainland | India |  | 0.7103 | nilgai | Gujarat, Western India |  |
| Chase et al 2018 | Modern |  | Faunal herbivore dung | | Mainland | India |  | 0.7107 | cattle/buffalo | Gujarat, Western India |  |
| Chase et al 2018 | Modern |  | Faunal herbivore dung | | Mainland | India |  | 0.7109 | cattle/buffalo | Gujarat, Western India |  |
| Chase et al 2018 | Modern |  | Faunal herbivore dung | | Mainland | India |  | 0.7106 | goat/sheep | Gujarat, Western India |  |
| Chase et al 2018 | Modern |  | Faunal herbivore dung | | Mainland | India |  | 0.7097 | cattle/buffalo | Gujarat, Western India |  |
| Chase et al 2018 | Modern |  | Faunal herbivore dung | | Mainland | India |  | 0.7097 | cattle/buffalo | Gujarat, Western India |  |
| Chase et al 2018 | Modern |  | Faunal herbivore dung | | Mainland | India |  | 0.7098 | snailshell | Gujarat, Western India |  |
| Chase et al 2018 | Modern |  | Faunal herbivore dung | | Mainland | India |  | 0.7106 | cattle/buffalo | Gujarat, Western India |  |
| Chase et al 2018 | Modern |  | Faunal herbivore dung | | Mainland | India |  | 0.7106 | cattle/buffalo | Gujarat, Western India |  |
| Chase et al 2018 | Modern |  | Faunal herbivore dung | | Mainland | India |  | 0.7105 | goat/sheep | Gujarat, Western India |  |
| Chase et al 2018 | Modern |  | Faunal herbivore dung | | Mainland | India |  | 0.7105 | cattle/buffalo | Gujarat, Western India |  |
| Chase et al 2018 | Modern |  | Faunal herbivore dung | | Mainland | India |  | 0.7106 | cattle/buffalo | Gujarat, Western India |  |
| Chase et al 2018 | Modern |  | Faunal herbivore dung | | Mainland | India |  | 0.7106 | cattle/buffalo | Gujarat, Western India |  |
| Chase et al 2018 | Modern |  | Faunal herbivore dung | | Mainland | India |  | 0.7104 | goat/sheep | Gujarat, Western India |  |
| Chase et al 2018 | Modern |  | Faunal herbivore dung | | Mainland | India |  | 0.7104 | goat/sheep | Gujarat, Western India |  |
| Chase et al 2018 | Modern |  | Faunal herbivore dung | | Mainland | India |  | 0.7106 | goat/sheep | Gujarat, Western India |  |
| Chase et al 2018 | Modern |  | Faunal herbivore dung | | Mainland | India |  | 0.7101 | cattle/buffalo | Gujarat, Western India |  |
| Chase et al 2018 | Modern |  | Faunal herbivore dung | | Mainland | India |  | 0.7101 | cattle/buffalo | Gujarat, Western India |  |
| Chase et al 2018 | Modern |  | Faunal herbivore dung | | Mainland | India |  | 0.7101 | goat/sheep | Gujarat, Western India |  |
| Chase et al 2018 | Modern |  | Faunal herbivore dung | | Saurashtra | India |  | 0.7095 | cattle/buffalo | Gujarat, Western India |  |
| Chase et al 2018 | Modern |  | Faunal herbivore dung | | Saurashtra | India |  | 0.7095 | goat/sheep | Gujarat, Western India |  |
| Chase et al 2018 | Modern |  | Faunal herbivore dung | | Saurashtra | India |  | 0.7094 | goat/sheep | Gujarat, Western India |  |
| Chase et al 2018 | Modern |  | Faunal herbivore dung | | Saurashtra | India |  | 0.7094 | cattle/buffalo | Gujarat, Western India |  |
| Chase et al 2018 | Modern |  | Faunal herbivore dung | | Saurashtra | India |  | 0.7094 | cattle/buffalo | Gujarat, Western India |  |
| Chase et al 2018 | Modern |  | Faunal herbivore dung | | Saurashtra | India |  | 0.7102 | cattle/buffalo | Gujarat, Western India |  |
| Chase et al 2018 | Modern |  | Faunal herbivore dung | | Saurashtra | India |  | 0.7099 | goat/sheep | Gujarat, Western India |  |
| Chase et al 2018 | Modern |  | Faunal herbivore dung | | Saurashtra | India |  | 0.7099 | nilgai | Gujarat, Western India |  |
| Chase et al 2018 | Modern |  | Faunal herbivore dung | | Saurashtra | India |  | 0.7103 | cattle/buffalo | Gujarat, Western India |  |
| Chase et al 2018 | Modern |  | Faunal herbivore dung | | Saurashtra | India |  | 0.7099 | cattle/buffalo | Gujarat, Western India |  |
| Chase et al 2018 | Modern |  | Faunal herbivore dung | | Saurashtra | India |  | 0.7099 | nilgai | Gujarat, Western India |  |
| Chase et al 2018 | Modern |  | Faunal herbivore dung | | Saurashtra | India |  | 0.7092 | cattle/buffalo | Gujarat, Western India |  |
| Chase et al 2018 | Modern |  | Faunal herbivore dung | | Saurashtra | India |  | 0.7092 | goat/sheep | Gujarat, Western India |  |
| Chase et al 2018 | Modern |  | Faunal herbivore dung | | Saurashtra | India |  | 0.7092 | goat/sheep | Gujarat, Western India |  |
| Chase et al 2018 | Modern |  | Faunal herbivore dung | | Saurashtra | India |  | 0.7095 | cattle/buffalo | Gujarat, Western India |  |
| Chase et al 2018 | Modern |  | Faunal herbivore dung | | Saurashtra | India |  | 0.7094 | goat/sheep | Gujarat, Western India |  |
| Chase et al 2018 | Modern |  | Faunal herbivore dung | | Saurashtra | India |  | 0.7094 | nilgai | Gujarat, Western India |  |
| Chase et al 2018 | Modern |  | Faunal herbivore dung | | Saurashtra | India |  | 0.7092 | cattle/buffalo | Gujarat, Western India |  |
| Chase et al 2018 | Modern |  | Faunal herbivore dung | | Saurashtra | India |  | 0.7087 | cattle/buffalo | Gujarat, Western India |  |
| Chase et al 2018 | Modern |  | Faunal herbivore dung | | Saurashtra | India |  | 0.7092 | cattle/buffalo | Gujarat, Western India |  |
| Chase et al 2018 | Modern |  | Faunal herbivore dung | | Saurashtra | India |  | 0.7094 | cattle/buffalo | Gujarat, Western India |  |
| Chase et al 2018 | Modern |  | Faunal herbivore dung | | Saurashtra | India |  | 0.7095 | cattle/buffalo | Gujarat, Western India |  |
| Chase et al 2018 | Modern |  | Faunal herbivore dung | | Saurashtra | India |  | 0.7093 | cattle/buffalo | Gujarat, Western India |  |
| Chase et al 2018 | Modern |  | Faunal herbivore dung | | Saurashtra | India |  | 0.7094 | goat/sheep | Gujarat, Western India |  |
| Chase et al 2018 | Modern |  | Faunal herbivore dung | | Saurashtra | India |  | 0.7093 | goat/sheep | Gujarat, Western India |  |
| Chase et al 2018 | Modern |  | Faunal herbivore dung | | Saurashtra | India |  | 0.7094 | goat/sheep | Gujarat, Western India |  |
| Chase et al 2018 | Modern |  | Faunal herbivore dung | | Saurashtra | India |  | 0.7094 | cattle/buffalo | Gujarat, Western India |  |
| Chase et al 2018 | Modern |  | Faunal herbivore dung | | Saurashtra | India |  | 0.7094 | cattle/buffalo | Gujarat, Western India |  |
| Chase et al 2018 | Modern |  | Faunal herbivore dung | | Saurashtra | India |  | 0.7093 | cattle/buffalo | Gujarat, Western India |  |
| Chase et al 2018 | Modern |  | Faunal herbivore dung | | Saurashtra | India |  | 0.7093 | goat/sheep | Gujarat, Western India |  |
| Chase et al 2018 | Modern |  | Faunal herbivore dung | | Saurashtra | India |  | 0.7093 | goat/sheep | Gujarat, Western India |  |
| Chase et al 2018 | Modern |  | Faunal herbivore dung | | Saurashtra | India |  | 0.7093 | nilgai | Gujarat, Western India |  |
| Chase et al 2018 | Modern |  | Faunal herbivore dung | | Saurashtra | India |  | 0.7093 | snailshell | Gujarat, Western India |  |
| Chase et al 2018 | Modern |  | Faunal herbivore dung | | Saurashtra | India |  | 0.7094 | snailshell | Gujarat, Western India |  |
| Chase et al 2018 | Modern |  | Faunal herbivore dung | | Saurashtra | India |  | 0.7092 | cattle/buffalo | Gujarat, Western India |  |
| Chase et al 2018 | Modern |  | Faunal herbivore dung | | Saurashtra | India |  | 0.7093 | cattle/buffalo | Gujarat, Western India |  |
| Chase et al 2018 | Modern |  | Faunal herbivore dung | | Saurashtra | India |  | 0.7092 | cattle/buffalo | Gujarat, Western India |  |
| Chase et al 2018 | Modern |  | Faunal herbivore dung | | Saurashtra | India |  | 0.7092 | cattle/buffalo | Gujarat, Western India |  |
| Chase et al 2018 | Modern |  | Faunal herbivore dung | | Saurashtra | India |  | 0.7092 | cattle/buffalo | Gujarat, Western India |  |
| Chase et al 2018 | Modern |  | Faunal herbivore dung | | Saurashtra | India |  | 0.7092 | cattle/buffalo | Gujarat, Western India |  |
| Chase et al 2018 | Modern |  | Faunal herbivore dung | | Saurashtra | India |  | 0.7093 | cattle/buffalo | Gujarat, Western India |  |
| Chase et al 2018 | Modern |  | Faunal herbivore dung | | Saurashtra | India |  | 0.7094 | cattle/buffalo | Gujarat, Western India |  |
| Chase et al 2018 | Modern |  | Faunal herbivore dung | | Saurashtra | India |  | 0.7094 | goat/sheep | Gujarat, Western India |  |
| Chase et al 2018 | Modern |  | Faunal herbivore dung | | Saurashtra | India |  | 0.7097 | cattle/buffalo | Gujarat, Western India |  |
| Chase et al 2018 | Modern |  | Faunal herbivore dung | | Saurashtra | India |  | 0.7097 | cattle/buffalo | Gujarat, Western India |  |
| Chase et al 2018 | Modern |  | Faunal herbivore dung | | Saurashtra | India |  | 0.7097 | goat/sheep | Gujarat, Western India |  |
| Chase et al 2018 | Modern |  | Faunal herbivore dung | | Saurashtra | India |  | 0.7092 | cattle/buffalo | Gujarat, Western India |  |
| Chase et al 2018 | Modern |  | Faunal herbivore dung | | Saurashtra | India |  | 0.7093 | cattle/buffalo | Gujarat, Western India |  |
| Chase et al 2018 | Modern |  | Faunal herbivore dung | | Saurashtra | India |  | 0.7092 | goat/sheep | Gujarat, Western India |  |
| Chase et al 2018 | Modern |  | Faunal herbivore dung | | Saurashtra | India |  | 0.7085 | cattle/buffalo | Gujarat, Western India |  |
| Chase et al 2018 | Modern |  | Faunal herbivore dung | | Saurashtra | India |  | 0.7081 | cattle/buffalo | Gujarat, Western India |  |
| Chase et al 2018 | Modern |  | Faunal herbivore dung | | Saurashtra | India |  | 0.7083 | cattle/buffalo | Gujarat, Western India |  |
| Chase et al 2018 | Modern |  | Faunal herbivore dung | | Saurashtra | India |  | 0.7091 | cattle/buffalo | Gujarat, Western India |  |
| Chase et al 2018 | Modern |  | Faunal herbivore dung | | Saurashtra | India |  | 0.7090 | goat/sheep | Gujarat, Western India |  |
| Chase et al 2018 | Modern |  | Faunal herbivore dung | | Saurashtra | India |  | 0.7090 | goat/sheep | Gujarat, Western India |  |
| Chase et al 2018 | Modern |  | Faunal herbivore dung | | Saurashtra | India |  | 0.7092 | cattle/buffalo | Gujarat, Western India |  |
| Chase et al 2018 | Modern |  | Faunal herbivore dung | | Saurashtra | India |  | 0.7092 | goat/sheep | Gujarat, Western India |  |
| Chase et al 2018 | Modern |  | Faunal herbivore dung | | Saurashtra | India |  | 0.7092 | nilgai | Gujarat, Western India |  |
| Chase et al 2018 | Modern |  | Faunal herbivore dung | | Kachchh | India |  | 0.7101 | cattle/buffalo | Gujarat, Western India |  |
| Chase et al 2018 | Modern |  | Faunal herbivore dung | | Kachchh | India |  | 0.7100 | cattle/buffalo | Gujarat, Western India |  |
| Chase et al 2018 | Modern |  | Faunal herbivore dung | | Kachchh | India |  | 0.7100 | goat/sheep | Gujarat, Western India |  |
| Chase et al 2018 | Modern |  | Faunal herbivore dung | | Kachchh | India |  | 0.7096 | cattle/buffalo | Gujarat, Western India |  |
| Chase et al 2018 | Modern |  | Faunal herbivore dung | | Kachchh | India |  | 0.7098 | cattle/buffalo | Gujarat, Western India |  |
| Chase et al 2018 | Modern |  | Faunal herbivore dung | | Kachchh | India |  | 0.7096 | goat/sheep | Gujarat, Western India |  |
| Chase et al 2018 | Modern |  | Faunal herbivore dung | | Kachchh | India |  | 0.7094 | goat/sheep | Gujarat, Western India |  |
| Chase et al 2018 | Modern |  | Faunal herbivore dung | | Kachchh | India |  | 0.7095 | goat/sheep | Gujarat, Western India |  |
| Chase et al 2018 | Modern |  | Faunal herbivore dung | | Kachchh | India |  | 0.7095 | goat/sheep | Gujarat, Western India |  |
| Chase et al 2018 | Modern |  | Faunal herbivore dung | | Kachchh | India |  | 0.7095 | nilgai | Gujarat, Western India |  |
| Chase et al 2018 | Modern |  | Faunal herbivore dung | | Kachchh | India |  | 0.7095 | nilgai | Gujarat, Western India |  |
| Chase et al 2018 | Modern |  | Faunal herbivore dung | | Kachchh | India |  | 0.7096 | nilgai | Gujarat, Western India |  |
| Chase et al 2018 | Modern |  | Faunal herbivore dung | | Kachchh | India |  | 0.7097 | cattle/buffalo | Gujarat, Western India |  |
| Chase et al 2018 | Modern |  | Faunal herbivore dung | | Kachchh | India |  | 0.7097 | cattle/buffalo | Gujarat, Western India |  |
| Chase et al 2018 | Modern |  | Faunal herbivore dung | | Kachchh | India |  | 0.7096 | goat/sheep | Gujarat, Western India |  |
| Chase et al 2018 | Modern |  | Faunal herbivore dung | | Kachchh | India |  | 0.7100 | cattle/buffalo | Gujarat, Western India |  |
| Chase et al 2018 | Modern |  | Faunal herbivore dung | | Kachchh | India |  | 0.7101 | cattle/buffalo | Gujarat, Western India |  |
| Chase et al 2018 | Modern |  | Faunal herbivore dung | | Kachchh | India |  | 0.7101 | goat/sheep | Gujarat, Western India |  |
| Chase et al 2018 | Modern |  | Faunal herbivore dung | | Kachchh | India |  | 0.7098 | cattle/buffalo | Gujarat, Western India |  |
| Chase et al 2018 | Modern |  | Faunal herbivore dung | | Kachchh | India |  | 0.7099 | cattle/buffalo | Gujarat, Western India |  |
| Chase et al 2018 | Modern |  | Faunal herbivore dung | | Kachchh | India |  | 0.7097 | goat/sheep | Gujarat, Western India |  |
| Chase et al 2018 | Modern |  | Faunal herbivore dung | | Kachchh | India |  | 0.7099 | goat/sheep | Gujarat, Western India |  |
| Chase et al 2018 | Modern |  | Faunal herbivore dung | | Kachchh | India |  | 0.7099 | goat/sheep | Gujarat, Western India |  |
| Chase et al 2018 | Modern |  | Faunal herbivore dung | | Kachchh | India |  | 0.7098 | cattle/buffalo | Gujarat, Western India |  |
| Chase et al 2018 | Modern |  | Faunal herbivore dung | | Kachchh | India |  | 0.7099 | goat/sheep | Gujarat, Western India |  |
| Chase et al 2018 | Modern |  | Faunal herbivore dung | | Kachchh | India |  | 0.7100 | goat/sheep | Gujarat, Western India |  |
| Chase et al 2018 | Modern |  | Faunal herbivore dung | | Kachchh | India |  | 0.7096 | nilgai | Gujarat, Western India |  |
| Chase et al 2018 | Modern |  | Faunal herbivore dung | | Kachchh | India |  | 0.7095 | cattle/buffalo | Gujarat, Western India |  |
| Chase et al 2018 | Modern |  | Faunal herbivore dung | | Kachchh | India |  | 0.7093 | goat/sheep | Gujarat, Western India |  |
| Chase et al 2018 | Modern |  | Faunal herbivore dung | | Kachchh | India |  | 0.7092 | goat/sheep | Gujarat, Western India |  |
| Chase et al 2018 | Modern |  | Faunal herbivore dung | | Kachchh | India |  | 0.7103 | cattle/buffalo | Gujarat, Western India |  |
| Chase et al 2018 | Modern |  | Faunal herbivore dung | | Kachchh | India |  | 0.7101 | cattle/buffalo | Gujarat, Western India |  |
| Chase et al 2018 | Modern |  | Faunal herbivore dung | | Kachchh | India |  | 0.7100 | cattle/buffalo | Gujarat, Western India |  |
| Chase et al 2018 | Modern |  | Faunal herbivore dung | | Kachchh | India |  | 0.7098 | cattle/buffalo | Gujarat, Western India |  |
| Chase et al 2018 | Modern |  | Faunal herbivore dung | | Kachchh | India |  | 0.7100 | cattle/buffalo | Gujarat, Western India |  |
| Chase et al 2018 | Modern |  | Faunal herbivore dung | | Kachchh | India |  | 0.7099 | cattle/buffalo | Gujarat, Western India |  |
| Chase et al 2018 | Modern |  | Faunal herbivore dung | | Kachchh | India |  | 0.7090 | cattle/buffalo | Gujarat, Western India |  |
| Chase et al 2018 | Modern |  | Faunal herbivore dung | | Kachchh | India |  | 0.7089 | cattle/buffalo | Gujarat, Western India |  |
| Chase et al 2018 | Modern |  | Faunal herbivore dung | | Kachchh | India |  | 0.7093 | goat/sheep | Gujarat, Western India |  |
| Chase et al 2018 | Modern |  | Faunal herbivore dung | | Kachchh | India |  | 0.7090 | goat/sheep | Gujarat, Western India |  |
| Chase et al 2018 | Modern |  | Faunal herbivore dung | | Kachchh | India |  | 0.7085 | nilgai | Gujarat, Western India |  |
| Chase et al 2018 | Modern |  | Faunal herbivore dung | | Kachchh | India |  | 0.7095 | cattle/buffalo | Gujarat, Western India |  |
| Chase et al 2018 | Modern |  | Faunal herbivore dung | | Kachchh | India |  | 0.7096 | cattle/buffalo | Gujarat, Western India |  |
| Chase et al 2018 | Modern |  | Faunal herbivore dung | | Kachchh | India |  | 0.7095 | cattle/buffalo | Gujarat, Western India |  |
| Chakraborty^82^ | Mature period (2600–1900 BCE) | | Faunal tooth enamel | KTB-22 | Kotada Bhadli | India |  | 0.7095 | Cattle/Buffalo | Gujarat, Western India |  |
| Chakraborty | Mature period (2600–1900 BCE) | | Faunal tooth enamel | KTB-23 | Kotada Bhadli | India |  | 0.7096 | Cattle/Buffalo | Gujarat, Western India |  |
| Chakraborty | Mature period (2600–1900 BCE) | | Faunal tooth enamel | KTB-24 | Kotada Bhadli | India |  | 0.7104 | Cattle/Buffalo | Gujarat, Western India |  |
| Chakraborty | Mature period (2600–1900 BCE) | | Faunal tooth enamel | KTB-25 | Kotada Bhadli | India |  | 0.7096 | Cattle/Buffalo | Gujarat, Western India |  |
| Chakraborty | Mature period (2600–1900 BCE) | | Faunal tooth enamel | KTB-26 | Kotada Bhadli | India |  | 0.7095 | Cattle/Buffalo | Gujarat, Western India |  |
| Chakraborty | Mature period (2600–1900 BCE) | | Faunal tooth enamel | KTB-27 | Kotada Bhadli | India |  | 0.7095 | Cattle/Buffalo | Gujarat, Western India |  |
| Chakraborty | Mature period (2600–1900 BCE) | | Faunal tooth enamel | KTB-29 | Kotada Bhadli | India |  | 0.7094 | Cattle/Buffalo | Gujarat, Western India |  |
| Chakraborty | Mature period (2600–1900 BCE) | | Faunal tooth enamel | KTB-30 | Kotada Bhadli | India |  | 0.7095 | Cattle/Buffalo | Gujarat, Western India |  |
| Chakraborty | Mature period (2600–1900 BCE) | | Faunal tooth enamel | KTB-31 | Kotada Bhadli | India |  | 0.7094 | Cattle/Buffalo | Gujarat, Western India |  |
| Chakraborty | Mature period (2600–1900 BCE) | | Faunal tooth enamel | KTB-32 | Kotada Bhadli | India |  | 0.7096 | Sheep | Gujarat, Western India |  |
| Chakraborty | Mature period (2600–1900 BCE) | | Faunal tooth enamel | KTB-33 | Kotada Bhadli | India |  | 0.7096 | SheepGoat | Gujarat, Western India |  |
| Chakraborty | Mature period (2600–1900 BCE) | | Faunal tooth enamel | KTB-38 | Kotada Bhadli | India |  | 0.7097 | SheepGoat | Gujarat, Western India |  |
| Chakraborty | Mature period (2600–1900 BCE) | | Faunal tooth enamel | KTB-39 | Kotada Bhadli | India |  | 0.7095 | SheepGoat | Gujarat, Western India |  |
| Chakraborty | Mature period (2600–1900 BCE) | | Faunal tooth enamel | KTB-40 | Kotada Bhadli | India |  | 0.7096 | SheepGoat | Gujarat, Western India |  |
| Chakraborty | Mature period (2600–1900 BCE) | | Faunal tooth enamel | KTB-41 | Kotada Bhadli | India |  | 0.7095 | SheepGoat | Gujarat, Western India |  |
| Chakraborty | Mature period (2600–1900 BCE) | | Faunal tooth enamel | KTB-42 | Kotada Bhadli | India |  | 0.7095 | SheepGoat | Gujarat, Western India |  |
| Chakraborty | Mature period (2600–1900 BCE) | | Faunal tooth enamel | KTB-43 | Kotada Bhadli | India |  | 0.7095 | SheepGoat | Gujarat, Western India |  |
| Chakraborty | Mature period (2600–1900 BCE) | | Faunal tooth enamel | KTB-44 | Kotada Bhadli | India |  | 0.7093 | SheepGoat | Gujarat, Western India |  |
|  |  |  |  |  |  |  |  |  |  |  |  |
| Kenoyer 2013^83^ | Late Harappa Phase/Harappan Phase/Early Harappan Phases | | Human tooth enamel | F4928 | Harappa | Pakistan | LLM2 | 0.7124 | *Homo* |  | H94/253_18 |
| Kenoyer 2013 | Late Harappa Phase/Harappan Phase/Early Harappan Phases | | Human tooth enamel | F4912 | Harappa | Pakistan | LLM2 | 0.7124 | *Homo* |  | H88/439_4b |
| Kenoyer 2013 | Late Harappa Phase/Harappan Phase/Early Harappan Phases | | Human tooth enamel | F4915 | Harappa | Pakistan | LLM2 | 0.7125 | *Homo* |  | H88/130_147a |
| Kenoyer 2013 | Late Harappa Phase/Harappan Phase/Early Harappan Phases | | Human tooth enamel | F4914 | Harappa | Pakistan | LLM1 | 0.7126 | *Homo* |  | H88/114_127a |
| Kenoyer 2013 | Late Harappa Phase/Harappan Phase/Early Harappan Phases | | Human tooth enamel | F4899 | Harappa | Pakistan | LRM1 | 0.7126 | *Homo* |  | H87/40-89_34b.1 |
| Kenoyer 2013 | Late Harappa Phase/Harappan Phase/Early Harappan Phases | | Human tooth enamel | F3930 | Harappa | Pakistan | LM3 | 0.7126 | *Homo* |  | H87/136_147a |
| Kenoyer 2013 | Late Harappa Phase/Harappan Phase/Early Harappan Phases | | Human tooth enamel | F4927 | Harappa | Pakistan | LLM1 | 0.7127 | *Homo* |  | H94/253_18 |
| Kenoyer 2013 | Late Harappa Phase/Harappan Phase/Early Harappan Phases | | Human tooth enamel | F4913 | Harappa | Pakistan | LRM1 | 0.7131 | *Homo* |  | H88/439_4a |
| Kenoyer 2013 | Late Harappa Phase/Harappan Phase/Early Harappan Phases | | Human tooth enamel | F4922 | Harappa | Pakistan | LLM2 | 0.7132 | *Homo* |  | H88/194_196a |
| Kenoyer 2013 | Late Harappa Phase/Harappan Phase/Early Harappan Phases | | Human tooth enamel | F4926 | Harappa | Pakistan | LLM2 | 0.7132 | *Homo* |  | H88/217_220a |
| Kenoyer 2013 | Late Harappa Phase/Harappan Phase/Early Harappan Phases | | Human tooth enamel | F4909 | Harappa | Pakistan | LLM1 | 0.7135 | *Homo* |  | H87/116_128a |
| Kenoyer 2013 | Late Harappa Phase/Harappan Phase/Early Harappan Phases | | Human tooth enamel | F4907 | Harappa | Pakistan | LRM1 | 0.7137 | *Homo* |  | H87/40_34a.2 |
| Kenoyer 2013 | Late Harappa Phase/Harappan Phase/Early Harappan Phases | | Human tooth enamel | F4906 | Harappa | Pakistan | LM | 0.7138 | *Homo* |  | H87/85_49d.1 |
| Kenoyer 2013 | Late Harappa Phase/Harappan Phase/Early Harappan Phases | | Human tooth enamel | F4898 | Harappa | Pakistan | URM1 | 0.7138 | *Homo* |  | H87/72_49b |
| Kenoyer 2013 | Late Harappa Phase/Harappan Phase/Early Harappan Phases | | Human tooth enamel | F4905 | Harappa | Pakistan | LRM2 | 0.7141 | *Homo* |  | H87/85_74a |
| Kenoyer 2013 | Late Harappa Phase/Harappan Phase/Early Harappan Phases | | Human tooth enamel | F4929 | Harappa | Pakistan | LRM1 | 0.7147 | *Homo* |  | H94/250_17 |
| Kenoyer 2013 | Late Harappa Phase/Harappan Phase/Early Harappan Phases | | Human tooth enamel | F4896 | Harappa | Pakistan | LLM1 | 0.7148 | *Homo* |  | H87/25_18A |
| Kenoyer 2013 | Late Harappa Phase/Harappan Phase/Early Harappan Phases | | Human tooth enamel | F4897 | Harappa | Pakistan | LLM2 | 0.7150 | *Homo* |  | H87/40_34A |
| Kenoyer 2013 | Late Harappa Phase/Harappan Phase/Early Harappan Phases | | Human tooth enamel | F4930 | Harappa | Pakistan | LLM1 | 0.7151 | *Homo* |  | H94/245_7 |
| Kenoyer 2013 | Late Harappa Phase/Harappan Phase/Early Harappan Phases | | Human tooth enamel | F4918 | Harappa | Pakistan | URM1 | 0.7153 | *Homo* |  | H88/174_126b.2 |
| Kenoyer 2013 | Late Harappa Phase/Harappan Phase/Early Harappan Phases | | Human tooth enamel | F4901 | Harappa | Pakistan | LM1 | 0.7160 | *Homo* |  | H87/71_49c |
| Kenoyer 2013 | Late Harappa Phase/Harappan Phase/Early Harappan Phases | | Human tooth enamel | F4931 | Harappa | Pakistan | LLM2 | 0.7161 | *Homo* |  | H94/243_27 |
| Kenoyer 2013 | Late Harappa Phase/Harappan Phase/Early Harappan Phases | | Human tooth enamel | F4908 | Harappa | Pakistan | LRM2 | 0.7169 | *Homo* |  | H87/200_203a |
| Kenoyer 2013 | Late Harappa Phase/Harappan Phase/Early Harappan Phases | | Human tooth enamel | F3927 | Harappa | Pakistan | LM2 | 0.7169 | *Homo* |  | H88/161_170 |
| Kenoyer 2013 | Late Harappa Phase/Harappan Phase/Early Harappan Phases | | Human tooth enamel | F4902 | Harappa | Pakistan | LRM2 | 0.7177 | *Homo* |  | H87/85_49h |
| Kenoyer 2013 | Late Harappa Phase/Harappan Phase/Early Harappan Phases | | Human tooth enamel | F3931 | Harappa | Pakistan | RM3 | 0.7178 | *Homo* |  | H87/72_49h |
| Kenoyer 2013 | Late Harappa Phase/Harappan Phase/Early Harappan Phases | | Human tooth enamel | F4900 | Harappa | Pakistan | LM3 | 0.7183 | *Homo* |  | H87/71_49c |
| Kenoyer 2013 | Late Harappa Phase/Harappan Phase/Early Harappan Phases | | Human tooth enamel | F4924 | Harappa | Pakistan | LM | 0.7184 | *Homo* |  | H88/206_208a |
| Kenoyer 2013 | Late Harappa Phase/Harappan Phase/Early Harappan Phases | | Human tooth enamel | F3928 | Harappa | Pakistan | *** | 0.7185 | *Homo* |  | H88/185_186 |
| Kenoyer 2013 | Late Harappa Phase/Harappan Phase/Early Harappan Phases | | Human tooth enamel | F4911 | Harappa | Pakistan | LLM1 | 0.7187 | *Homo* |  | H87/145_156a |
| Kenoyer 2013 | Late Harappa Phase/Harappan Phase/Early Harappan Phases | | Human tooth enamel | F4925 | Harappa | Pakistan | ULM1 | 0.7189 | *Homo* |  | H88/216_219a |
| Kenoyer 2013 | Late Harappa Phase/Harappan Phase/Early Harappan Phases | | Human tooth enamel | F4916 | Harappa | Pakistan | LRm2 | 0.7193 | *Homo* |  | H88/173_133a.10 |
| Kenoyer 2013 | Late Harappa Phase/Harappan Phase/Early Harappan Phases | | Human tooth enamel | F4917 | Harappa | Pakistan | LLM2 | 0.7193 | *Homo* |  | H88/173_133a.10 |
| Kenoyer 2013 | Late Harappa Phase/Harappan Phase/Early Harappan Phases | | Human tooth enamel | F4903 | Harappa | Pakistan | LLM2 | 0.7193 | *Homo* |  | H87/85_49g |
| Kenoyer 2013 | Late Harappa Phase/Harappan Phase/Early Harappan Phases | | Human tooth enamel | F4910 | Harappa | Pakistan | ULM1 | 0.7195 | *Homo* |  | H87/141_152a |
| Kenoyer 2013 | Late Harappa Phase/Harappan Phase/Early Harappan Phases | | Human tooth enamel | F3932 | Harappa | Pakistan | *** | 0.7210 | *Homo* |  | H87/72_49h |
| Kenoyer 2013 | Late Harappa Phase/Harappan Phase/Early Harappan Phases | | Human tooth enamel | F3929 | Harappa | Pakistan | *** | 0.7212 | *Homo* |  | H88/162_121 |
| Kenoyer 2013 | Late Harappa Phase/Harappan Phase/Early Harappan Phases | | Human tooth enamel | F4923 | Harappa | Pakistan | UM | 0.7216 | *Homo* |  | H88/206_208a |
| Kenoyer 2013 | Late Harappa Phase/Harappan Phase/Early Harappan Phases | | Human tooth enamel | F4904 | Harappa | Pakistan | *** | 0.7218 | *Homo* |  | H87/85_49g |
| Kenoyer 2013 | Late Harappa Phase/Harappan Phase/Early Harappan Phases | | Human tooth enamel | F4921 | Harappa | Pakistan | URM2 | 0.7276 | *Homo* |  | H88/191_185c.1 |
| Kenoyer 2013 | Modern |  | Faunal tooth enamel | F3926 | Harappa | Pakistan |  | 0.7158 | Bos |  |  |
| Kenoyer 2013 | Modern |  | Faunal tooth enamel | F3924 | Harappa | Pakistan |  | 0.7167 | Bos |  |  |
| Kenoyer 2013 | Modern |  | Faunal tooth enamel | F3918 | Harappa | Pakistan |  | 0.7168 | o/c |  |  |
| Kenoyer 2013 | Modern |  | Faunal tooth enamel | F3920 | Harappa | Pakistan |  | 0.7175 | o/c |  |  |
| Kenoyer 2013 | Modern |  | Faunal tooth enamel | F3925 | Harappa | Pakistan |  | 0.7183 | Bos |  |  |
| Kenoyer 2013 | Modern |  | Faunal tooth enamel | F3919 | Harappa | Pakistan |  | 0.7183 | o/c |  |  |
| Kenoyer 2013 | Modern |  | Faunal tooth enamel | F3923 | Harappa | Pakistan |  | 0.7187 | Sus |  |  |
| Kenoyer 2013 | Modern |  | Faunal tooth enamel | F3921 | Harappa | Pakistan |  | 0.7187 | Sus |  |  |
| Kenoyer 2013 | Modern |  | Faunal tooth enamel | F3922 | Harappa | Pakistan |  | 0.7189 | Sus |  |  |
| Vanentine et al 2015 | Harappa Phase (2600–1900 BC) | | Human Tooth Enamel | H87/ 25 18a | Harappa | Pakistan | LM_1_ | 0.71485 | *Homo* |  |  |
| Vanentine et al 2015 | Harappa Phase (2600–1900 BC) | | Human Tooth Enamel | H87/ 40 34a | Harappa | Pakistan | LM_2_ | 0.71459 | *Homo* |  |  |
| Vanentine et al 2015 | Harappa Phase (2600–1900 BC) | | Human Tooth Enamel | H87/ 40 34a.2 | Harappa | Pakistan | RM_1_ | 0.71360 | *Homo* |  |  |
| Vanentine et al 2015 | Harappa Phase (2600–1900 BC) | | Human Tooth Enamel | H87/ 40-89 34b.1 | Harappa | Pakistan | RM_1_ | 0.71236 | *Homo* |  |  |
| Vanentine et al 2015 | Harappa Phase (2600–1900 BC) | | Human Tooth Enamel | H87/ 71 49b1 | Harappa | Pakistan | RM_3_ | 0.71343 | *Homo* |  |  |
| Vanentine et al 2015 | Harappa Phase (2600–1900 BC) | | Human Tooth Enamel | H87/ 71 49c | Harappa | Pakistan | M_1_ | 0.71585 | *Homo* |  |  |
| Vanentine et al 2015 | Harappa Phase (2600–1900 BC) | | Human Tooth Enamel | H87/ 71 49c | Harappa | Pakistan | M_3_ | 0.71828 | *Homo* |  |  |
| Vanentine et al 2015 | Harappa Phase (2600–1900 BC) | | Human Tooth Enamel | H87/ 72 49h | Harappa | Pakistan | LI_1_ | 0.71321 | *Homo* |  |  |
| Vanentine et al 2015 | Harappa Phase (2600–1900 BC) | | Human Tooth Enamel | H87/ 72 49h | Harappa | Pakistan | LC | 0.71741 | *Homo* |  |  |
| Vanentine et al 2015 | Harappa Phase (2600–1900 BC) | | Human Tooth Enamel | H87/ 72 49h | Harappa | Pakistan | M_1_ | 0.71754 | *Homo* |  |  |
| Vanentine et al 2015 | Harappa Phase (2600–1900 BC) | | Human Tooth Enamel | H87/ 72 49b | Harappa | Pakistan | RM^1^ | 0.71415 | *Homo* |  |  |
| Vanentine et al 2015 | Harappa Phase (2600–1900 BC) | | Human Tooth Enamel | H87/ 85 49g | Harappa | Pakistan | LM_2_ | 0.71922 | *Homo* |  |  |
| Vanentine et al 2015 | Harappa Phase (2600–1900 BC) | | Human Tooth Enamel | H87/ 85 49g | Harappa | Pakistan | LM_2_ | 0.72190 | *Homo* |  |  |
| Vanentine et al 2015 | Harappa Phase (2600–1900 BC) | | Human Tooth Enamel | H87/ 85 49h | Harappa | Pakistan | RM_2_ | 0.71760 | *Homo* |  |  |
| Vanentine et al 2015 | Harappa Phase (2600–1900 BC) | | Human Tooth Enamel | H87/ 91 80a | Harappa | Pakistan | RM^2^ | 0.71659 | *Homo* |  |  |
| Vanentine et al 2015 | Harappa Phase (2600–1900 BC) | | Human Tooth Enamel | H87/ 92 81a | Harappa | Pakistan | LM^3^ | 0.71817 | *Homo* |  |  |
| Vanentine et al 2015 | Harappa Phase (2600–1900 BC) | | Human Tooth Enamel | H87/ 108 126a | Harappa | Pakistan | RM^3^ | 0.71880 | *Homo* |  |  |
| Vanentine et al 2015 | Harappa Phase (2600–1900 BC) | | Human Tooth Enamel | H87/ 116 128a | Harappa | Pakistan | LM_1_ | 0.71305 | *Homo* |  |  |
| Vanentine et al 2015 | Harappa Phase (2600–1900 BC) | | Human Tooth Enamel | H87/ 136 147a | Harappa | Pakistan | LM_1_ | 0.71272 | *Homo* |  |  |
| Vanentine et al 2015 | Harappa Phase (2600–1900 BC) | | Human Tooth Enamel | H87/ 141 152a | Harappa | Pakistan | LM^1^ | 0.71962 | *Homo* |  |  |
| Vanentine et al 2015 | Harappa Phase (2600–1900 BC) | | Human Tooth Enamel | H87/ 145 156a | Harappa | Pakistan | LM_1_ | 0.71853 | *Homo* |  |  |
| Vanentine et al 2015 | Harappa Phase (2600–1900 BC) | | Human Tooth Enamel | H87/ 200 203a | Harappa | Pakistan | RM_2_ | 0.71691 | *Homo* |  |  |
| Vanentine et al 2015 | Harappa Phase (2600–1900 BC) | | Human Tooth Enamel | H88/ 114 127a | Harappa | Pakistan | LM_1_ | 0.71230 | *Homo* |  |  |
| Vanentine et al 2015 | Harappa Phase (2600–1900 BC) | | Human Tooth Enamel | H88/ 130 147a | Harappa | Pakistan | LM_2_ | 0.71202 | *Homo* |  |  |
| Vanentine et al 2015 | Harappa Phase (2600–1900 BC) | | Human Tooth Enamel | H88/ 162 121 | Harappa | Pakistan | RP^3^ | 0.72123 | *Homo* |  |  |
| Vanentine et al 2015 | Harappa Phase (2600–1900 BC) | | Human Tooth Enamel | H88/ 173 133a.10 | Harappa | Pakistan | LM_2_ | 0.71930 | *Homo* |  |  |
| Vanentine et al 2015 | Harappa Phase (2600–1900 BC) | | Human Tooth Enamel | H88/ 174 126b.2 | Harappa | Pakistan | RM^1^ | 0.71546 | *Homo* |  |  |
| Vanentine et al 2015 | Harappa Phase (2600–1900 BC) | | Human Tooth Enamel | H88/ 174 126b.2 | Harappa | Pakistan | RM^3^ | 0.71543 | *Homo* |  |  |
| Vanentine et al 2015 | Harappa Phase (2600–1900 BC) | | Human Tooth Enamel | H88/ 180 170a.17 | Harappa | Pakistan | LM^1^ | 0.71367 | *Homo* |  |  |
| Vanentine et al 2015 | Harappa Phase (2600–1900 BC) | | Human Tooth Enamel | H88/ 180 170a.17 | Harappa | Pakistan | LM^3^ | 0.71286 | *Homo* |  |  |
| Vanentine et al 2015 | Harappa Phase (2600–1900 BC) | | Human Tooth Enamel | H88/ 191 185c.1 | Harappa | Pakistan | RM^2^ | 0.72707 | *Homo* |  |  |
| Vanentine et al 2015 | Harappa Phase (2600–1900 BC) | | Human Tooth Enamel | H88/ 191 185f | Harappa | Pakistan | LM_1_ | 0.72802 | *Homo* |  |  |
| Vanentine et al 2015 | Harappa Phase (2600–1900 BC) | | Human Tooth Enamel | H88/ 194 196a | Harappa | Pakistan | LM_2_ | 0.71275 | *Homo* |  |  |
| Vanentine et al 2015 | Harappa Phase (2600–1900 BC) | | Human Tooth Enamel | H88/ 198 200a | Harappa | Pakistan | LM^2^ | 0.71980 | *Homo* |  |  |
| Vanentine et al 2015 | Harappa Phase (2600–1900 BC) | | Human Tooth Enamel | H88/ 201 204a | Harappa | Pakistan | RM_1_ | 0.71951 | *Homo* |  |  |
| Vanentine et al 2015 | Harappa Phase (2600–1900 BC) | | Human Tooth Enamel | H88/ 216 219a | Harappa | Pakistan | LM^1^ | 0.71896 | *Homo* |  |  |
| Vanentine et al 2015 | Harappa Phase (2600–1900 BC) | | Human Tooth Enamel | H88/ 217 220a | Harappa | Pakistan | LM_2_ | 0.71300 | *Homo* |  |  |
| Vanentine et al 2015 | Harappa Phase (2600–1900 BC) | | Human Tooth Enamel | H88/ 439 4b | Harappa | Pakistan | LM_2_ | 0.71258 | *Homo* |  |  |
| Vanentine et al 2015 | Harappa Phase (2600–1900 BC) | | Human Tooth Enamel | H94/ 243 5#2 | Harappa | Pakistan | LM^2^ | 0.71113 | *Homo* |  |  |
| Vanentine et al 2015 | Harappa Phase (2600–1900 BC) | | Human Tooth Enamel | H94/ 243 27 | Harappa | Pakistan | LM_2_ | 0.71604 | *Homo* |  |  |
| Vanentine et al 2015 | Harappa Phase (2600–1900 BC) | | Human Tooth Enamel | H94/ 245 7 | Harappa | Pakistan | LM_1_ | 0.71475 | *Homo* |  |  |
| Vanentine et al 2015 | Harappa Phase (2600–1900 BC) | | Human Tooth Enamel | H94/ 250 17 | Harappa | Pakistan | RM_1_ | 0.71480 | *Homo* |  |  |
| Vanentine et al 2015 | Harappa Phase (2600–1900 BC) | | Human Tooth Enamel | H94/ 253 18 | Harappa | Pakistan | LM_1_ | 0.71274 | *Homo* |  |  |
| Vanentine et al 2015 | Harappa Phase (2600–1900 BC) | | Human Tooth Enamel | H94/ 253 18 | Harappa | Pakistan | LM_2_ | 0.71248 | *Homo* |  |  |
| Valentine 2013 | Harappa Phase (2600-1900 BC) | | Archaeological faunal tooth enamel | HC1 | Harappa | Pakistan |  | 0.71828 | Canis |  |  |
| Valentine 2013 | Harappa Phase (2600-1900 BC) | | Archaeological faunal tooth enamel | HC2 | Harappa | Pakistan |  | 0.71797 | Canis |  |  |
| Valentine 2013 | Harappa Phase (2600-1900 BC) | | Archaeological faunal tooth enamel | HC3 | Harappa | Pakistan |  | 0.71828 | Canis |  |  |
| Valentine 2013 | Harappa Phase (2600-1900 BC) | | Archaeological faunal tooth enamel | HS1 | Harappa | Pakistan |  | 0.71913 | Sus |  |  |
| Valentine 2013 | Harappa Phase (2600-1900 BC) | | Archaeological faunal tooth enamel | HS2 | Harappa | Pakistan |  | 0.72112 | Sus |  |  |
| Valentine 2013 | Harappa Phase (2600-1900 BC) | | Archaeological faunal tooth enamel | HS3 | Harappa | Pakistan |  | 0.71796 | Sus |  |  |
| Valentine 2013 | Harappa Phase (2600-1900 BC) | | Archaeological faunal tooth enamel | HS4 | Harappa | Pakistan |  | 0.72084 | Sus |  |  |
| Valentine 2013 | Harappa Phase (2600-1900 BC) | | Archaeological faunal tooth enamel | HS5 | Harappa | Pakistan |  | 0.71569 | Sus |  |  |
| Valentine 2013 | Harappa Phase (2600-1900 BC) | | Archaeological faunal tooth enamel | HS6 | Harappa | Pakistan |  | 0.71855 | Sus |  |  |
| Valentine 2013 | Harappa Phase (2600-1900 BC) | | Archaeological faunal tooth enamel | HS7 | Harappa | Pakistan |  | 0.71795 | Sus |  |  |
| Valentine 2013 | Harappa Phase (2600-1900 BC) | | Archaeological faunal tooth enamel | HS8 | Harappa | Pakistan |  | 0.71908 | Sus |  |  |
| Valentine 2013 | Harappa Phase (2600-1900 BC) | | Archaeological faunal tooth enamel | HA05 | Harappa | Pakistan |  | 0.71892 | Sus |  |  |
| Valentine 2013 | Harappa Phase (2600-1900 BC) | | Archaeological faunal tooth enamel | HA06 | Harappa | Pakistan |  | 0.71869 | Sus |  |  |
| Gregoricka 2013^84^ | Bronze age: Umm an-Nar | | Faunal Tooth Enamel | ALL88 | Allahdino | Pakistan | RM2 | 0.708645 | Sheep/goat |  |  |
| Gregoricka 2013 | Bronze age: Umm an-Nar | | Faunal Tooth Enamel | ALL89 | Allahdino | Pakistan | LM2/3 | 0.708759 | Sheep/goat |  |  |
| Gregoricka 2013 | Bronze age: Umm an-Nar | | Faunal Tooth Enamel | ALL90 | Allahdino | Pakistan | RM2 | 0.708764 | Goat |  |  |
| Gregoricka 2013 | Bronze age: Umm an-Nar | | Faunal Tooth Enamel | ALL91 | Allahdino | Pakistan | LM2 | 0.708719 | Goat |  |  |
| Gregoricka 2013 | Bronze age: Umm an-Nar | | Faunal Tooth Enamel | ALL92 | Allahdino | Pakistan | LM2 | 0.708757 | Sheep/goat |  |  |
| Gregoricka 2013 | Bronze age: Umm an-Nar | | Faunal Tooth Enamel | ALL93 | Allahdino | Pakistan | LM3 | 0.709133 | Goat |  |  |
| Gregoricka 2013 | Bronze age: Umm an-Nar | | Faunal Tooth Enamel | ALL94 | Allahdino | Pakistan | RPM4 | 0.708720 | Cattle |  |  |
| Gregoricka 2013 | Bronze age: Umm an-Nar | | Faunal Tooth Enamel | ALL95 | Allahdino | Pakistan | LM1 | 0.710417 | Goat |  |  |
| Gregoricka 2013 | Bronze age: Umm an-Nar | | Faunal Tooth Enamel | ALL96 | Allahdino | Pakistan | LM2 | 0.708781 | Goat |  |  |
| Gregoricka 2013 | Bronze age: Umm an-Nar | | Faunal Tooth Enamel | ALL97 | Allahdino | Pakistan | RM3 | 0.708446 | Goat |  |  |
| Valentine 2013 | Harappa Phase (2600-1900 BC) | | Archaeological faunal tooth enamel | AS1 | Allahdino | Pakistan |  | 0.70876 | Sus |  |  |
| Valentine 2013 | Harappa Phase (2600-1900 BC) | | Archaeological faunal tooth enamel | AS2 | Allahdino | Pakistan |  | 0.71082 | Sus |  |  |
| Valentine 2013 | Harappa Phase (2600-1900 BC) | | Archaeological faunal tooth enamel | AS3b | Allahdino | Pakistan |  | 0.70873 | Sus |  |  |
| Valentine 2013 | Harappa Phase (2600-1900 BC) | | Archaeological faunal tooth enamel | AS4 | Allahdino | Pakistan |  | 0.71088 | Sus |  |  |
| Valentine 2013 | Harappa Phase (2600-1900 BC) | | Archaeological faunal tooth enamel | AS5 | Allahdino | Pakistan |  | 0.70870 | Sus |  |  |
| Valentine 2013 | Harappa Phase (2600-1900 BC) | | Archaeological faunal tooth enamel | MC1 | Mehrgarh | Pakistan |  | 0.70815 | Canis |  |  |
| Valentine 2013 | Harappa Phase (2600-1900 BC) | | Archaeological faunal tooth enamel | MC2 | Mehrgarh | Pakistan |  | 0.70802 | Canis |  |  |
| Valentine 2013 | Harappa Phase (2600-1900 BC) | | Archaeological faunal tooth enamel | NE1 | Nausharo | Pakistan |  | 0.70827 | Equus |  |  |
| Valentine 2013 | Harappa Phase (2600-1900 BC) | | Archaeological faunal tooth enamel | NE2 | Nausharo | Pakistan |  | 0.70825 | Equus |  |  |
| Valentine 2013 | Harappa Phase (2600-1900 BC) | | Archaeological faunal tooth enamel | NE3 | Nausharo | Pakistan |  | 0.70821 | Equus |  |  |
| Valentine 2013 | Harappa Phase (2600-1900 BC) | | Archaeological faunal tooth enamel | NE4 | Nausharo | Pakistan |  | 0.7082 | Equus |  |  |
| Valentine 2013 | Harappa Phase (2600-1900 BC) | | Archaeological faunal tooth enamel | NG1 | Nausharo | Pakistan |  | 0.70811 | Gazella |  |  |
| Valentine 2013 | Harappa Phase (2600-1900 BC) | | Archaeological faunal tooth enamel | NG2 | Nausharo | Pakistan |  | 0.70816 | Gazella |  |  |
| Gregoricka 2013 | Bronze age: Umm an-Nar | | Faunal Tooth Enamel | Bal78 | Balakot | Pakistan | LM2 | 0.709113 | Gazelle |  |  |
| Gregoricka 2013 | Bronze age: Umm an-Nar | | Faunal Tooth Enamel | Bal79 | Balakot | Pakistan | RI1 | 0.708855 | Pig |  |  |
| Gregoricka 2013 | Bronze age: Umm an-Nar | | Faunal Tooth Enamel | Bal80 | Balakot | Pakistan | RM1 | 0.709668 | Cattle |  |  |
| Gregoricka 2013 | Bronze age: Umm an-Nar | | Faunal Tooth Enamel | Bal81 | Balakot | Pakistan | LM1/2 | 0.708886 | Cattle |  |  |
| Gregoricka 2013 | Bronze age: Umm an-Nar | | Faunal Tooth Enamel | Bal82 | Balakot | Pakistan | RM1/2 | 0.708955 | Sheep/goat |  |  |
| Gregoricka 2013 | Bronze age: Umm an-Nar | | Faunal Tooth Enamel | Bal83 | Balakot | Pakistan | RM3 | 0.708901 | Sheep/goat |  |  |
| Gregoricka 2013 | Bronze age: Umm an-Nar | | Faunal Tooth Enamel | Bal84 | Balakot | Pakistan | RM3 | 0.708745 | Cattle |  |  |
| Gregoricka 2013 | Bronze age: Umm an-Nar | | Faunal Tooth Enamel | Bal85 | Balakot | Pakistan | RM3 | 0.708887 | Cattle |  |  |
| Gregoricka 2013 | Bronze age: Umm an-Nar | | Faunal Tooth Enamel | Bal86 | Balakot | Pakistan | LM2 | 0.708911 | Cattle |  |  |
| Gregoricka 2013 | Bronze age: Umm an-Nar | | Faunal Tooth Enamel | Bal87 | Balakot | Pakistan | RM3 | 0.708877 | Cattle |  |  |
| Gregoricka 2013 | Bronze age: Umm an-Nar | | Faunal Tooth Enamel | TY68 | TepeYahya | Iran | LM3 | 0.708301 | Cattle |  |  |
| Gregoricka 2013 | Bronze age: Umm an-Nar | | Faunal Tooth Enamel | TY69 | TepeYahya | Iran | RPM3 | 0.708214 | Cattle |  |  |
| Gregoricka 2013 | Bronze age: Umm an-Nar | | Faunal Tooth Enamel | TY70 | TepeYahya | Iran | RM1/2 | 0.708094 | Cattle |  |  |
| Gregoricka 2013 | Bronze age: Umm an-Nar | | Faunal Tooth Enamel | TY71 | TepeYahya | Iran | RM3 | 0.708652 | Sheep/goat |  |  |
| Gregoricka 2013 | Bronze age: Umm an-Nar | | Faunal Tooth Enamel | TY72 | TepeYahya | Iran | LM3 | 0.708184 | Sheep/goat |  |  |
| Gregoricka 2013 | Bronze age: Umm an-Nar | | Faunal Tooth Enamel | TY73 | TepeYahya | Iran | LM3 | 0.708267 | Sheep/goat |  |  |
| Gregoricka 2013 | Bronze age: Umm an-Nar | | Faunal Tooth Enamel | TY74 | TepeYahya | Iran | LM3 | 0.708221 | Sheep/goat |  |  |
| Gregoricka 2013 | Bronze age: Umm an-Nar | | Faunal Tooth Enamel | TY75 | TepeYahya | Iran | LM3 | 0.708382 | Sheep/goat |  |  |
| Gregoricka 2013 | Bronze age: Umm an-Nar | | Faunal Tooth Enamel | TY76 | TepeYahya | Iran | RM3 | 0.708121 | Pig |  |  |
| Gregoricka 2013 | Bronze age: Umm an-Nar | | Faunal Tooth Enamel | TY77 | TepeYahya | Iran | RM3 | 0.708132 | Pig |  |  |
| Kenoyer 2013 | Late Harappa Phase/Harappan Phase/Early Harappan Phases | | Human tooth enamel | F4894 | Ur | Iraq |  | 0.7080 | *Homo* |  | 30-12-551 |
| Kenoyer 2013^83^ | Late Harappa Phase/Harappan Phase/Early Harappan Phases | | Human tooth enamel | F4895 | Ur | Iraq |  | 0.7081 | *Homo* |  | B17312 |
| Gregoricka 2013 | Bronze age: Umm an-Nar | | Faunal Tooth Enamel | FAI21 | Failaka | Kuwait | LM2 | 0.708861 | Cattle |  |  |
| Gregoricka 2013 | Bronze age: Umm an-Nar | | Faunal Tooth Enamel | FAI22 | Failaka | Kuwait | RM2 | 0.708580 | Cattle |  |  |
| Gregoricka 2013 | Bronze age: Umm an-Nar | | Faunal Tooth Enamel | FAI23 | Failaka | Kuwait | RM3 | 0.708669 | Cattle |  |  |
| Gregoricka 2013 | Bronze age: Umm an-Nar | | Faunal Tooth Enamel | FAI24 | Failaka | Kuwait | LM3 | 0.708750 | Cattle |  |  |
| Gregoricka 2013 | Bronze age: Umm an-Nar | | Faunal Tooth Enamel | FAI25 | Failaka | Kuwait | LM2 | 0.708571 | Cattle |  |  |
| Gregoricka 2013 | Bronze age: Umm an-Nar | | Faunal Tooth Enamel | FAI26 | Failaka | Kuwait | LM2 | 0.708669 | Cattle |  |  |
| Gregoricka 2013 | Bronze age: Umm an-Nar | | Faunal Tooth Enamel | FAI27 | Failaka | Kuwait | LM2 | 0.708417 | Cattle |  |  |
| Gregoricka 2013 | Bronze age: Umm an-Nar | | Faunal Tooth Enamel | FAI28 | Failaka | Kuwait | LM3 | 0.708423 | Sheep/goat |  |  |
| Gregoricka 2013 | Bronze age: Umm an-Nar | | Faunal Tooth Enamel | FAI29 | Failaka | Kuwait | LM2 | 0.709023 | Sheep/goat |  |  |
| Gregoricka 2013 | Bronze age: Umm an-Nar | | Faunal Tooth Enamel | FAI30 | Failaka | Kuwait | RM2 | 0.709011 | Sheep/goat |  |  |
| Gregoricka 2013 | Bronze age: Umm an-Nar | | Faunal Tooth Enamel | FAI31 | Failaka | Kuwait | RM3 | 0.709024 | Sheep/goat |  |  |
| Gregoricka 2013 | Bronze age: Umm an-Nar | | Faunal Tooth Enamel | FAI32 | Failaka | Kuwait | LM3 | 0.708427 | Sheep/goat |  |  |
| Gregoricka 2013 | Bronze age: Umm an-Nar | | Faunal Tooth Enamel | FAI33 | Failaka | Kuwait | LM3 | 0.708392 | Sheep/goat |  |  |
| Gregoricka 2013 | Bronze age: Umm an-Nar | | Faunal Tooth Enamel | FAI34 | Failaka | Kuwait | LM1/2 | 0.708447 | Sheep/goat |  |  |
| Gregoricka 2013 | Bronze age: Umm an-Nar | | Faunal Tooth Enamel | FAI35 | Failaka | Kuwait | RM2 | 0.708410 | Sheep/goat |  |  |
|  |  |  |  |  |  |  |  |  |  |  |  |
| Gregoricka 2013 | Bronze age: Umm an-Nar | | Faunal Tooth Enamel | BM1 | AaliBurialMounds | Bahrain | LM1 | 0.708263 | Sheep/goat |  |  |
| Gregoricka 2013 | Bronze age: Umm an-Nar | | Faunal Tooth Enamel | BM2 | AaliBurialMounds | Bahrain | LM2 | 0.708233 | Sheep/goat |  |  |
| Gregoricka 2013 | Bronze age: Umm an-Nar | | Faunal Tooth Enamel | BM3 | AaliBurialMounds | Bahrain | LM3 | 0.708168 | Sheep/goat |  |  |
| Gregoricka 2013 | Bronze age: Umm an-Nar | | Faunal Tooth Enamel | BM4 | AaliBurialMounds | Bahrain | LM2 | 0.708209 | Sheep/goat |  |  |
| Gregoricka 2013 | Bronze age: Umm an-Nar | | Faunal Tooth Enamel | BM5 | AaliBurialMounds | Bahrain | LM2 | 0.708523 | Sheep/goat |  |  |
| Gregoricka 2013 | Bronze age: Umm an-Nar | | Faunal Tooth Enamel | Bar6 | Barbar | Bahrain | LM1 | 0.708107 | Cattle |  |  |
| Gregoricka 2013 | Bronze age: Umm an-Nar | | Faunal Tooth Enamel | Bar7 | Barbar | Bahrain | LM2 | 0.708303 | Cattle |  |  |
| Gregoricka 2013 | Bronze age: Umm an-Nar | | Faunal Tooth Enamel | Bar8 | Barbar | Bahrain | RM3 | 0.708184 | Cattle |  |  |
| Gregoricka 2013 | Bronze age: Umm an-Nar | | Faunal Tooth Enamel | Bar9 | Barbar | Bahrain | RM1 | 0.708108 | Cattle |  |  |
| Gregoricka 2013 | Bronze age: Umm an-Nar | | Faunal Tooth Enamel | Bar10 | Barbar | Bahrain | LM1 | 0.708082 | Cattle |  |  |
| Gregoricka 2013 | Bronze age: Umm an-Nar | | Faunal Tooth Enamel | Bar11 | Barbar | Bahrain | RM1 | 0.708572 | Cattle |  |  |
| Gregoricka 2013 | Bronze age: Umm an-Nar | | Faunal Tooth Enamel | Bar12 | Barbar | Bahrain | LM2 | 0.708174 | Cattle |  |  |
| Gregoricka 2013 | Bronze age: Umm an-Nar | | Faunal Tooth Enamel | Bar13 | Barbar | Bahrain | LM2 | 0.708170 | Cattle |  |  |
| Gregoricka 2013 | Bronze age: Umm an-Nar | | Faunal Tooth Enamel | Bar14 | Barbar | Bahrain | LM3 | 0.708472 | Sheep/goat |  |  |
| Gregoricka 2013 | Bronze age: Umm an-Nar | | Faunal Tooth Enamel | Bar15 | Barbar | Bahrain | LM3 | 0.708320 | Sheep/goat |  |  |
| Gregoricka 2013 | Bronze age: Umm an-Nar | | Faunal Tooth Enamel | Bar16 | Barbar | Bahrain | LM3 | 0.708256 | Sheep/goat |  |  |
| Gregoricka 2013 | Bronze age: Umm an-Nar | | Faunal Tooth Enamel | Bar17 | Barbar | Bahrain | RM3 | 0.708349 | Sheep/goat |  |  |
| Gregoricka 2013 | Bronze age: Umm an-Nar | | Faunal Tooth Enamel | Bar18 | Barbar | Bahrain | RM1 | 0.708240 | Sheep/goat |  |  |
| Gregoricka 2013 | Bronze age: Umm an-Nar | | Faunal Tooth Enamel | Bar19 | Barbar | Bahrain | RM1 | 0.708114 | Sheep/goat |  |  |
| Gregoricka 2013 | Bronze age: Umm an-Nar | | Faunal Tooth Enamel | Bar20 | Barbar | Bahrain | LM1 | 0.708518 | Sheep/goat |  |  |
|  |  |  |  |  |  |  |  |  |  |  |  |
| Gregoricka 2013 | Bronze age: Umm an-Nar | | Faunal Tooth Enamel | Qid52 | Qidfa | UAE | RM1 | 0.708535 | Sheep/goat |  |  |
| Gregoricka et al 2014^85^ | Bronze age |  | Human tooth enamel | Qid250/251 | Qidfa | UAE | M1 | 0.70867 | *Homo* |  |  |
| Gregoricka et al 2014 | Bronze age |  | Human tooth enamel | Qid250/251 | Qidfa | UAE | M2 | 0.70870 | *Homo* |  |  |
| Gregoricka et al 2014 | Bronze age |  | Human tooth enamel | Bid245/246 | Bidya | UAE | M1 | 0.70864 | *Homo* |  |  |
| Gregoricka et al 2014 | Bronze age |  | Human tooth enamel | Bid245/246 | Bidya | UAE | M2 | 0.70823 | *Homo* |  |  |
| Gregoricka 2013 | Bronze age: Umm an-Nar | | Faunal Tooth Enamel | Dib51 | Dibba | UAE | LM1 | 0.708686 | Sheep/goat |  |  |
| Gregoricka et al 2014 | Bronze age |  | Human tooth enamel | Dib252/253 | Dibba | UAE | M2 | 0.70880 | *Homo* |  |  |
| Gregoricka et al 2014 | Bronze age |  | Human tooth enamel | Dib252/253 | Dibba | UAE | M3 | 0.70883 | *Homo* |  |  |
| Gregoricka et al 2014 | Bronze age |  | Human tooth enamel | Dib254/255 | Dibba | UAE | M1 | 0.70906 | *Homo* |  |  |
| Gregoricka et al 2014 | Bronze age |  | Human tooth enamel | Dib254/255 | Dibba | UAE | M2 | 0.70876 | *Homo* |  |  |
| Gregoricka 2013 | Bronze age: Umm an-Nar | 2400e2200BC | Human tooth enamel | RAK216 | Shimal | UAE | LM1 | 0.708819 | *Homo* | Ras_al-Khaimah | Unar_1 |
| Gregoricka 2013 | Bronze age: Umm an-Nar | 2400e2200BC | Human tooth enamel | RAK217 | Shimal | UAE | LM1 | 0.708757 | *Homo* | Ras_al-Khaimah | Unar_1 |
| Gregoricka 2013 | Bronze age: Umm an-Nar | 2400e2200BC | Human tooth enamel | RAK218 | Shimal | UAE | LM1 | 0.708789 | *Homo* | Ras_al-Khaimah | Unar_1 |
| Gregoricka 2013 | Bronze age: Umm an-Nar | 2400e2200BC | Human tooth enamel | RAK219 | Shimal | UAE | LM1 | 0.708770 | *Homo* | Ras_al-Khaimah | Unar_1 |
| Gregoricka 2013 | Bronze age: Umm an-Nar | 2400e2200BC | Human tooth enamel | RAK220 | Shimal | UAE | LM1 | 0.708748 | *Homo* | Ras_al-Khaimah | Unar_1 |
| Gregoricka 2013 | Bronze age: Umm an-Nar | 2400e2200BC | Human tooth enamel | RAK221 | Shimal | UAE | LM1 | 0.709012 | *Homo* | Ras_al-Khaimah | Unar_1 |
| Gregoricka 2013 | Bronze age: Umm an-Nar | 2400e2200BC | Human tooth enamel | RAK222 | Shimal | UAE | LM1 | 0.708795 | *Homo* | Ras_al-Khaimah | Unar_1 |
| Gregoricka 2013 | Bronze age: Umm an-Nar | 2400e2200BC | Human tooth enamel | RAK223 | Shimal | UAE | LM1 | 0.708767 | *Homo* | Ras_al-Khaimah | Unar_1 |
| Gregoricka 2013 | Bronze age: Umm an-Nar | 2400e2200BC | Human tooth enamel | RAK224 | Shimal | UAE | LM1 | 0.708793 | *Homo* | Ras_al-Khaimah | Unar_1 |
| Gregoricka 2013 | Bronze age: Umm an-Nar | 2400e2200BC | Human tooth enamel | RAK225 | Shimal | UAE | LM1 | 0.708788 | *Homo* | Ras_al-Khaimah | Unar_1 |
| Gregoricka 2013 | Bronze age: Umm an-Nar | 2400e2200BC | Human tooth enamel | RAK226 | Shimal | UAE | LM1 | 0.708820 | *Homo* | Ras_al-Khaimah | Unar_1 |
| Gregoricka 2013 | Bronze age: Umm an-Nar | 2400e2200BC | Human tooth enamel | RAK228 | Shimal | UAE | LM1 | 0.708777 | *Homo* | Ras_al-Khaimah | Unar_1 |
| Gregoricka 2013 | Bronze age: Umm an-Nar | 2400e2200BC | Human tooth enamel | RAK229 | Shimal | UAE | LM1 | 0.708737 | *Homo* | Ras_al-Khaimah | Unar_1 |
| Gregoricka 2013 | Bronze age: Umm an-Nar | 2400e2200BC | Human tooth enamel | RAK230 | Shimal | UAE | LM1 | 0.708865 | *Homo* | Ras_al-Khaimah | Unar_1 |
| Gregoricka 2013 | Bronze age: Umm an-Nar | 2400e2200BC | Human tooth enamel | RAK231 | Shimal | UAE | LM1 | 0.708789 | *Homo* | Ras_al-Khaimah | Unar_1 |
| Gregoricka 2013 | Bronze age: Umm an-Nar | 2400e2200BC | Human tooth enamel | RAK232 | Shimal | UAE | LM1 | 0.708758 | *Homo* | Ras_al-Khaimah | Unar_1 |
| Gregoricka 2013 | Bronze age: Umm an-Nar | 2400e2200BC | Human tooth enamel | RAK233 | Shimal | UAE | LM1 | 0.708750 | *Homo* | Ras_al-Khaimah | Unar_1 |
| Gregoricka 2013 | Bronze age: Umm an-Nar | 2400e2200BC | Human tooth enamel | RAK235 | Shimal | UAE | LM1 | 0.708754 | *Homo* | Ras_al-Khaimah | Unar_1 |
| Gregoricka 2013 | Bronze age: Umm an-Nar | 2400e2200BC | Human tooth enamel | RAK236 | Shimal | UAE | LM1 | 0.708783 | *Homo* | Ras_al-Khaimah | Unar_1 |
| Gregoricka 2013 | Bronze age: Umm an-Nar | 2400e2200BC | Human tooth enamel | RAK237 | Shimal | UAE | LM1 | 0.708852 | *Homo* | Ras_al-Khaimah | Unar_1 |
| Gregoricka 2013 | Bronze age: Umm an-Nar | 2400e2200BC | Human tooth enamel | RAK238 | Shimal | UAE | LM1 | 0.708968 | *Homo* | Ras_al-Khaimah | Unar_1 |
| Gregoricka 2013 | Bronze age: Umm an-Nar | 2400e2200BC | Human tooth enamel | RAK239 | Shimal | UAE | LM1 | 0.708820 | *Homo* | Ras_al-Khaimah | Unar_1 |
| Gregoricka 2013 | Bronze age: Umm an-Nar | 2400e2200BC | Human tooth enamel | RAK241 | Shimal | UAE | LM1 | 0.708784 | *Homo* | Ras_al-Khaimah | Unar_1 |
| Gregoricka 2013 | Bronze age: Umm an-Nar | 2400e2200BC | Human tooth enamel | RAK242 | Shimal | UAE | LM1 | 0.708801 | *Homo* | Ras_al-Khaimah | Unar_1 |
| Gregoricka 2013 | Bronze age: Umm an-Nar | 2400e2200BC | Human tooth enamel | RAK243 | Shimal | UAE | LM1 | 0.708832 | *Homo* | Ras_al-Khaimah | Unar_1 |
| Gregoricka 2013 | Bronze age: Umm an-Nar | | Faunal Tooth Enamel | SH110 | Shimal | UAE | RM1/2 | 0.708890 | Sheep/goat |  |  |
| Gregoricka 2013 | Bronze age: Umm an-Nar | | Faunal Tooth Enamel | SH111 | Shimal | UAE | RM1/2 | 0.708646 | Sheep/goat |  |  |
| Gregoricka 2013 | Bronze age: Umm an-Nar | | Faunal Tooth Enamel | SH112 | Shimal | UAE | LM3 | 0.708768 | Sheep/goat |  |  |
| Gregoricka 2013 | Bronze age: Umm an-Nar | | Faunal Tooth Enamel | SH113 | Shimal | UAE | RM1/2 | 0.708846 | Sheep/goat |  |  |
| Gregoricka 2013 | Bronze age: Umm an-Nar | | Faunal Tooth Enamel | SH114 | Shimal | UAE | LM1 | 0.708973 | Sheep/goat |  |  |
| Gregoricka 2013 | Bronze age: Umm an-Nar | | Faunal Tooth Enamel | SH115 | Shimal | UAE | LM1/2 | 0.708686 | Sheep/goat |  |  |
| Gregoricka 2013 | Bronze age: Umm an-Nar | | Faunal Tooth Enamel | SH117 | Shimal | UAE | RM1 | 0.708856 | Sheep/goat |  |  |
| Gregoricka 2013 | Bronze age: Umm an-Nar | | Faunal Tooth Enamel | SH118 | Shimal | UAE | LM3 | 0.708649 | Sheep/goat |  |  |
| Gregoricka 2013 | Bronze age: Umm an-Nar | | Faunal Tooth Enamel | SH119 | Shimal | UAE | LM1 | 0.708859 | Sheep/goat |  |  |
| Gregoricka 2013 | Bronze age: Umm an-Nar | | Faunal Tooth Enamel | SH116 | Shimal | UAE | LP3/4 | 0.708711 | Sheep/goat |  |  |
| Gregoricka et al 2014 | Bronze age |  | Human tooth enamel | RAK226/227 | Unar1 | UAE | M1 | 0.70882 | *Homo* |  |  |
| Gregoricka et al 2014 | Bronze age |  | Human tooth enamel | RAK226/227 | Unar1 | UAE | M2 | 0.70881 | *Homo* |  |  |
| Gregoricka et al 2014 | Bronze age |  | Human tooth enamel | RAK233/234 | Unar1 | UAE | M1 | 0.70875 | *Homo* |  |  |
| Gregoricka et al 2014 | Bronze age |  | Human tooth enamel | RAK233/234 | Unar1 | UAE | M2 | 0.70876 | *Homo* |  |  |
| Gregoricka et al 2014 | Bronze age |  | Human tooth enamel | RAK239/240 | Unar1 | UAE | M1 | 0.70882 | *Homo* |  |  |
| Gregoricka et al 2014 | Bronze age |  | Human tooth enamel | RAK239/240 | Unar1 | UAE | M2 | 0.70890 | *Homo* |  |  |
| Kutterer et al 2015^86^ | Middle Sasanian period | | Human tooth enamel | 549 | Jabel Emeilah | UAE | M1 | 0.7081 | *Homo* |  |  |
| Kutterer et al 2015 | Middle Sasanian period | | Human tooth enamel | 550 | Jabel Emeilah | UAE | M3 | 0.7080 | *Homo* |  |  |
| Kutterer et al 2015 | Middle Sasanian period | | Human tooth enamel | 552 | Jabel Emeilah | UAE | M1 | 0.7080 | *Homo* |  |  |
| Kutterer et al 2015 | Middle Sasanian period | | Human tooth enamel | 553 | Jabel Emeilah | UAE | M3 | 0.7081 | *Homo* |  |  |
| Gregoricka 2013 | Bronze age: Umm an-Nar | 2300e2100BC | Human tooth enamel | MW188 | Mowaihat | UAE | LM1 | 0.708866 | *Homo* | Ajman | Tomb_B |
| Gregoricka 2013 | Bronze age: Umm an-Nar | 2300e2100BC | Human tooth enamel | MW189 | Mowaihat | UAE | LM1 | 0.708835 | *Homo* | Ajman | Tomb_B |
| Gregoricka 2013 | Bronze age: Umm an-Nar | 2300e2100BC | Human tooth enamel | MW190 | Mowaihat | UAE | LM1 | 0.708865 | *Homo* | Ajman | Tomb_B |
| Gregoricka 2013 | Bronze age: Umm an-Nar | 2300e2100BC | Human tooth enamel | MW192 | Mowaihat | UAE | LM1 | 0.708863 | *Homo* | Ajman | Tomb_B |
| Gregoricka 2013 | Bronze age: Umm an-Nar | 2300e2100BC | Human tooth enamel | MW194 | Mowaihat | UAE | LM1 | 0.708868 | *Homo* | Ajman | Tomb_B |
| Gregoricka 2013 | Bronze age: Umm an-Nar | 2300e2100BC | Human tooth enamel | MW195 | Mowaihat | UAE | LM1 | 0.708858 | *Homo* | Ajman | Tomb_B |
| Gregoricka 2013 | Bronze age: Umm an-Nar | 2300e2100BC | Human tooth enamel | MW197 | Mowaihat | UAE | LM1 | 0.708582 | *Homo* | Ajman | Tomb_B |
| Gregoricka 2013 | Bronze age: Umm an-Nar | 2300e2100BC | Human tooth enamel | MW198 | Mowaihat | UAE | LM1 | 0.708844 | *Homo* | Ajman | Tomb_B |
| Gregoricka 2013 | Bronze age: Umm an-Nar | 2300e2100BC | Human tooth enamel | MW199 | Mowaihat | UAE | LM1 | 0.708859 | *Homo* | Ajman | Tomb_B |
| Gregoricka 2013 | Bronze age: Umm an-Nar | 2300e2100BC | Human tooth enamel | MW200 | Mowaihat | UAE | LM1 | 0.708873 | *Homo* | Ajman | Tomb_B |
| Gregoricka 2013 | Bronze age: Umm an-Nar | 2300e2100BC | Human tooth enamel | MW202 | Mowaihat | UAE | LM1 | 0.708879 | *Homo* | Ajman | Tomb_B |
| Gregoricka 2013 | Bronze age: Umm an-Nar | 2300e2100BC | Human tooth enamel | MW204 | Mowaihat | UAE | LM1 | 0.708838 | *Homo* | Ajman | Tomb_B |
| Gregoricka 2013 | Bronze age: Umm an-Nar | 2300e2100BC | Human tooth enamel | MW206 | Mowaihat | UAE | LM1 | 0.708860 | *Homo* | Ajman | Tomb_B |
| Gregoricka et al 2014 | Bronze age |  | Human tooth enamel | MW190/191 | Mowaihat | UAE | M1 | 0.70887 | *Homo* |  |  |
| Gregoricka et al 2014 | Bronze age |  | Human tooth enamel | MW190/191 | Mowaihat | UAE | M3 | 0.70886 | *Homo* |  |  |
| Gregoricka et al 2014 | Bronze age |  | Human tooth enamel | MW192/193 | Mowaihat | UAE | M1 | 0.70886 | *Homo* |  |  |
| Gregoricka et al 2014 | Bronze age |  | Human tooth enamel | MW192/193 | Mowaihat | UAE | M3 | 0.70887 | *Homo* |  |  |
| Gregoricka et al 2014 | Bronze age |  | Human tooth enamel | MW195/196 | Mowaihat | UAE | M1 | 0.70886 | *Homo* |  |  |
| Gregoricka et al 2014 | Bronze age |  | Human tooth enamel | MW195/196 | Mowaihat | UAE | M2 | 0.70886 | *Homo* |  |  |
| Gregoricka et al 2014 | Bronze age |  | Human tooth enamel | MW200/201 | Mowaihat | UAE | M1 | 0.70887 | *Homo* |  |  |
| Gregoricka et al 2014 | Bronze age |  | Human tooth enamel | MW200/201 | Mowaihat | UAE | M2 | 0.70887 | *Homo* |  |  |
| Gregoricka et al 2014 | Bronze age |  | Human tooth enamel | MW202/203 | Mowaihat | UAE | M1 | 0.70888 | *Homo* |  |  |
| Gregoricka et al 2014 | Bronze age |  | Human tooth enamel | MW202/203 | Mowaihat | UAE | M3 | 0.70888 | *Homo* |  |  |
| Gregoricka et al 2014 | Bronze age |  | Human tooth enamel | MW204/205 | Mowaihat | UAE | M1 | 0.70884 | *Homo* |  |  |
| Gregoricka et al 2014 | Bronze age |  | Human tooth enamel | MW204/205 | Mowaihat | UAE | M3 | 0.70888 | *Homo* |  |  |
| Kutterer et al 2017^87^ | Neolithic |  | Faunal tooth enamel | 331 | al-Buhais 18 | UAE |  | 0.70863 | Bos primigenius |  |  |
| Kutterer et al 2017 | Neolithic |  | Faunal tooth enamel | 334 | al-Buhais 18 | UAE |  | 0.70865 | Bos primigenius |  |  |
| Kutterer et al 2017 | Neolithic |  | Faunal tooth enamel | 337 | al-Buhais 18 | UAE |  | 0.70866 | BOS |  |  |
| Kutterer et al 2017 | Neolithic |  | Faunal tooth enamel | 338 | al-Buhais 18 | UAE |  | 0.70865 | BOS |  |  |
| Kutterer et al 2017 | Neolithic |  | Faunal tooth enamel | 347 | al-Buhais 18 | UAE |  | 0.70866 | BOS |  |  |
| Kutterer et al 2017 | Neolithic |  | Faunal tooth enamel | 353 | al-Buhais 18 | UAE |  | 0.70865 | BOS |  |  |
| Kutterer et al 2017 | Neolithic |  | Faunal tooth enamel | 784 | al-Buhais 18 | UAE |  | 0.70869 | Capra aegagrus |  |  |
| Kutterer et al 2017 | Neolithic |  | Faunal tooth enamel | 785 | al-Buhais 18 | UAE |  | 0.70862 | Capra aegagrus |  |  |
| Kutterer et al 2017 | Neolithic |  | Human tooth enamel | AK | al-Buhais 18 | UAE | M1 | 0.70866 | *Homo* |  | 534/535 |
| Kutterer et al 2017 | Neolithic |  | Human tooth enamel | AK | al-Buhais 18 | UAE | M3 | 0.70868 | *Homo* |  |  |
| Kutterer et al 2017 | Neolithic |  | Human tooth enamel | BN | al-Buhais 18 | UAE | M1 | 0.70867 | *Homo* |  | 774/775 |
| Kutterer et al 2017 | Neolithic |  | Human tooth enamel | BN | al-Buhais 18 | UAE | M3 | 0.70867 | *Homo* |  |  |
| Kutterer et al 2017 | Neolithic |  | Human tooth enamel | BP | al-Buhais 18 | UAE | M1 | 0.70862 | *Homo* |  | 586/587 |
| Kutterer et al 2017 | Neolithic |  | Human tooth enamel | BP | al-Buhais 18 | UAE | M3 | 0.70862 | *Homo* |  |  |
| Kutterer et al 2017 | Neolithic |  | Human tooth enamel | BX | al-Buhais 18 | UAE | M1 | 0.70867 | *Homo* |  | 780/781 |
| Kutterer et al 2017 | Neolithic |  | Human tooth enamel | BX | al-Buhais 18 | UAE | M3 | 0.70866 | *Homo* |  |  |
| Kutterer et al 2017 | Neolithic |  | Human tooth enamel | BY | al-Buhais 18 | UAE | M1 | 0.70864 | *Homo* |  | 778/779 |
| Kutterer et al 2017 | Neolithic |  | Human tooth enamel | BY | al-Buhais 18 | UAE | M3 | 0.70860 | *Homo* |  |  |
| Kutterer et al 2017 | Neolithic |  | Human tooth enamel | CN | al-Buhais 18 | UAE | M1 | 0.70863 | *Homo* |  | 590/591 |
| Kutterer et al 2017 | Neolithic |  | Human tooth enamel | CN | al-Buhais 18 | UAE | M3 | 0.70866 | *Homo* |  |  |
| Kutterer et al 2017 | Neolithic |  | Human tooth enamel | CR | al-Buhais 18 | UAE | M1 | 0.70875 | *Homo* |  | 580/581 |
| Kutterer et al 2017 | Neolithic |  | Human tooth enamel | CR | al-Buhais 18 | UAE | M3 | 0.70874 | *Homo* |  |  |
| Kutterer et al 2017 | Neolithic |  | Human tooth enamel | CU | al-Buhais 18 | UAE | M1 | 0.70869 | *Homo* |  | 567/568 |
| Kutterer et al 2017 | Neolithic |  | Human tooth enamel | CU | al-Buhais 18 | UAE | M3 | 0.70863 | *Homo* |  |  |
| Kutterer et al 2017 | Neolithic |  | Human tooth enamel | DD | al-Buhais 18 | UAE | M1 | 0.70859 | *Homo* |  | 782/783 |
| Kutterer et al 2017 | Neolithic |  | Human tooth enamel | DD | al-Buhais 18 | UAE | M3 | 0.70865 | *Homo* |  |  |
| Kutterer et al 2017 | Neolithic |  | Human tooth enamel | DN | al-Buhais 18 | UAE | M1 | 0.70869 | *Homo* |  | 582/583 |
| Kutterer et al 2017 | Neolithic |  | Human tooth enamel | DN | al-Buhais 18 | UAE | M3 | 0.70869 | *Homo* |  |  |
| Kutterer et al 2017 | Neolithic |  | Human tooth enamel | EG | al-Buhais 18 | UAE | M1 | 0.70861 | *Homo* |  | 776/777 |
| Kutterer et al 2017 | Neolithic |  | Human tooth enamel | EG | al-Buhais 18 | UAE | M3 | 0.70863 | *Homo* |  |  |
| Kutterer et al 2017 | Neolithic |  | Human tooth enamel | EH | al-Buhais 18 | UAE | M1 | 0.70869 | *Homo* |  | 536/537 |
| Kutterer et al 2017 | Neolithic |  | Human tooth enamel | EH | al-Buhais 18 | UAE | M3 | 0.7087 | *Homo* |  |  |
| Kutterer et al 2017 | Neolithic |  | Human tooth enamel | ET | al-Buhais 18 | UAE | M1 | 0.70867 | *Homo* |  | 592/593 |
| Kutterer et al 2017 | Neolithic |  | Human tooth enamel | ET | al-Buhais 18 | UAE | M3 | 0.70871 | *Homo* |  |  |
| Kutterer et al 2017 | Neolithic |  | Human tooth enamel | FJ | al-Buhais 18 | UAE | M1 | 0.70862 | *Homo* |  | 770/771 |
| Kutterer et al 2017 | Neolithic |  | Human tooth enamel | FJ | al-Buhais 18 | UAE | M3 | 0.70864 | *Homo* |  |  |
| Kutterer et al 2017 | Neolithic |  | Human tooth enamel | FQ1 | al-Buhais 18 | UAE | M1 | 0.70865 | *Homo* |  | 697 |
| Kutterer et al 2017 | Neolithic |  | Human tooth enamel | FQ2 | al-Buhais 18 | UAE | M1 | 0.70865 | *Homo* |  | 696 |
| Kutterer et al 2017 | Neolithic |  | Human tooth enamel | GQ | al-Buhais 18 | UAE | M1 | 0.70865 | *Homo* |  | 772/773 |
| Kutterer et al 2017 | Neolithic |  | Human tooth enamel | GQ | al-Buhais 18 | UAE | M3 | 0.70866 | *Homo* |  |  |
| Kutterer et al 2017 | Neolithic |  | Human tooth enamel | HS | al-Buhais 18 | UAE | M1 | 0.70868 | *Homo* |  | 768/769 |
| Kutterer et al 2017 | Neolithic |  | Human tooth enamel | HS | al-Buhais 18 | UAE | M3 | 0.70863 | *Homo* |  |  |
| Kutterer et al 2017 | Neolithic |  | Human tooth enamel | TG | al-Buhais 18 | UAE | M1 | 0.70865 | *Homo* |  | 538/539 |
| Kutterer et al 2017 | Neolithic |  | Human tooth enamel | TG | al-Buhais 18 | UAE | M3 | 0.70864 | *Homo* |  |  |
| Kutterer et al 2017 | Neolithic |  | Human tooth enamel | TO | al-Buhais 18 | UAE | M1 | 0.70866 | *Homo* |  | 594/595 |
| Kutterer et al 2017 | Neolithic |  | Human tooth enamel | TO | al-Buhais 18 | UAE | M3 | 0.70865 | *Homo* |  |  |
| Kutterer et al 2017 | Neolithic |  | Human tooth enamel | TP | al-Buhais 18 | UAE | M1 | 0.7086 | *Homo* |  | 588/589 |
| Kutterer et al 2017 | Neolithic |  | Human tooth enamel | TP | al-Buhais 18 | UAE | M3 | 0.70867 | *Homo* |  |  |
| Kutterer et al 2017 | Neolithic |  | Human tooth enamel | Sk. 4 | Umm al-Quwain 2 | UAE | M1 | 0.70858 | *Homo* |  | 540/541 |
| Kutterer et al 2017 | Neolithic |  | Human tooth enamel | Sk. 4 | Umm al-Quwain 2 | UAE | M3 | 0.70858 | *Homo* |  | 540/541 |
| Kutterer et al 2017 | Neolithic |  | Human tooth enamel | Sk. 13 | Umm al-Quwain 2 | UAE | M1 | 0.70888 | *Homo* |  | 558/559 |
| Kutterer et al 2017 | Neolithic |  | Human tooth enamel | Sk. 13 | Umm al-Quwain 2 | UAE | M3 | 0.70892 | *Homo* |  | 558/559 |
| Kutterer et al 2017 | Neolithic |  | Human tooth enamel | Sk. 18 | Umm al-Quwain 2 | UAE | M1 | 0.70881 | *Homo* |  | 560/561 |
| Kutterer et al 2017 | Neolithic |  | Human tooth enamel | Sk. 18 | Umm al-Quwain 2 | UAE | M3 | 0.70882 | *Homo* |  | 560/561 |
| Kutterer et al 2017 | Neolithic |  | Human tooth enamel | Sk. 20 | Umm al-Quwain 2 | UAE | M1 | 0.70884 | *Homo* |  | 556/557 |
| Kutterer et al 2017 | Neolithic |  | Human tooth enamel | Sk. 20 | Umm al-Quwain 2 | UAE | M3 | 0.70883 | *Homo* |  | 556/557 |
| Kutterer et al 2017 | Neolithic |  | Human tooth enamel | Sk. 32 | Umm al-Quwain 2 | UAE | M1 | 0.70878 | *Homo* |  | 554/555 |
| Kutterer et al 2017 | Neolithic |  | Human tooth enamel | Sk. 32 | Umm al-Quwain 2 | UAE | M3 | 0.70879 | *Homo* |  | 554/555 |
| Gregoricka 2013 | Bronze age: Umm an-Nar | 2200e2000BC | Human tooth enamel | TA158 | TellAbraq | UAE | LM1 | 0.708887 | *Homo* | Sharjah | TellAbraq |
| Gregoricka 2013 | Bronze age: Umm an-Nar | 2200e2000BC | Human tooth enamel | TA159 | TellAbraq | UAE | LM1 | 0.708899 | *Homo* | Sharjah | TellAbraq |
| Gregoricka 2013 | Bronze age: Umm an-Nar | 2200e2000BC | Human tooth enamel | TA160 | TellAbraq | UAE | LM1 | 0.708866 | *Homo* | Sharjah | TellAbraq |
| Gregoricka 2013 | Bronze age: Umm an-Nar | 2200e2000BC | Human tooth enamel | TA161 | TellAbraq | UAE | LM1 | 0.710661 | *Homo* | Sharjah | TellAbraq |
| Gregoricka 2013 | Bronze age: Umm an-Nar | 2200e2000BC | Human tooth enamel | TA162 | TellAbraq | UAE | LM1 | 0.708875 | *Homo* | Sharjah | TellAbraq |
| Gregoricka 2013 | Bronze age: Umm an-Nar | 2200e2000BC | Human tooth enamel | TA163 | TellAbraq | UAE | LM1 | 0.708839 | *Homo* | Sharjah | TellAbraq |
| Gregoricka 2013 | Bronze age: Umm an-Nar | 2200e2000BC | Human tooth enamel | TA164 | TellAbraq | UAE | LM1 | 0.708877 | *Homo* | Sharjah | TellAbraq |
| Gregoricka 2013 | Bronze age: Umm an-Nar | 2200e2000BC | Human tooth enamel | TA165 | TellAbraq | UAE | LM1 | 0.708179 | *Homo* | Sharjah | TellAbraq |
| Gregoricka 2013 | Bronze age: Umm an-Nar | 2200e2000BC | Human tooth enamel | TA166 | TellAbraq | UAE | LM1 | 0.708906 | *Homo* | Sharjah | TellAbraq |
| Gregoricka 2013 | Bronze age: Umm an-Nar | 2200e2000BC | Human tooth enamel | TA167 | TellAbraq | UAE | LM1 | 0.708878 | *Homo* | Sharjah | TellAbraq |
| Gregoricka 2013 | Bronze age: Umm an-Nar | 2200e2000BC | Human tooth enamel | TA168 | TellAbraq | UAE | LM1 | 0.708869 | *Homo* | Sharjah | TellAbraq |
| Gregoricka 2013 | Bronze age: Umm an-Nar | 2200e2000BC | Human tooth enamel | TA169 | TellAbraq | UAE | LM1 | 0.708878 | *Homo* | Sharjah | TellAbraq |
| Gregoricka 2013 | Bronze age: Umm an-Nar | 2200e2000BC | Human tooth enamel | TA170 | TellAbraq | UAE | LM1 | 0.708862 | *Homo* | Sharjah | TellAbraq |
| Gregoricka 2013 | Bronze age: Umm an-Nar | 2200e2000BC | Human tooth enamel | TA171 | TellAbraq | UAE | LM1 | 0.708884 | *Homo* | Sharjah | TellAbraq |
| Gregoricka 2013 | Bronze age: Umm an-Nar | 2200e2000BC | Human tooth enamel | TA172 | TellAbraq | UAE | LM1 | 0.708886 | *Homo* | Sharjah | TellAbraq |
| Gregoricka 2013 | Bronze age: Umm an-Nar | 2200e2000BC | Human tooth enamel | TA173 | TellAbraq | UAE | LM1 | 0.708856 | *Homo* | Sharjah | TellAbraq |
| Gregoricka 2013 | Bronze age: Umm an-Nar | 2200e2000BC | Human tooth enamel | TA174 | TellAbraq | UAE | LM1 | 0.708863 | *Homo* | Sharjah | TellAbraq |
| Gregoricka 2013 | Bronze age: Umm an-Nar | 2200e2000BC | Human tooth enamel | TA175 | TellAbraq | UAE | LM1 | 0.708896 | *Homo* | Sharjah | TellAbraq |
| Gregoricka 2013 | Bronze age: Umm an-Nar | 2200e2000BC | Human tooth enamel | TA176 | TellAbraq | UAE | LM1 | 0.708901 | *Homo* | Sharjah | TellAbraq |
| Gregoricka 2013 | Bronze age: Umm an-Nar | 2200e2000BC | Human tooth enamel | TA177 | TellAbraq | UAE | LM1 | 0.708862 | *Homo* | Sharjah | TellAbraq |
| Gregoricka 2013 | Bronze age: Umm an-Nar | 2200e2000BC | Human tooth enamel | TA178 | TellAbraq | UAE | LM1 | 0.708871 | *Homo* | Sharjah | TellAbraq |
| Gregoricka 2013 | Bronze age: Umm an-Nar | 2200e2000BC | Human tooth enamel | TA179 | TellAbraq | UAE | LM1 | 0.708820 | *Homo* | Sharjah | TellAbraq |
| Gregoricka 2013 | Bronze age: Umm an-Nar | 2200e2000BC | Human tooth enamel | TA180 | TellAbraq | UAE | LM1 | 0.708888 | *Homo* | Sharjah | TellAbraq |
| Gregoricka 2013 | Bronze age: Umm an-Nar | 2200e2000BC | Human tooth enamel | TA181 | TellAbraq | UAE | LM1 | 0.708884 | *Homo* | Sharjah | TellAbraq |
| Gregoricka 2013 | Bronze age: Umm an-Nar | 2200e2000BC | Human tooth enamel | TA182 | TellAbraq | UAE | LM1 | 0.708863 | *Homo* | Sharjah | TellAbraq |
| Gregoricka 2013 | Bronze age: Umm an-Nar | 2200e2000BC | Human tooth enamel | TA183 | TellAbraq | UAE | LM1 | 0.708878 | *Homo* | Sharjah | TellAbraq |
| Gregoricka 2013 | Bronze age: Umm an-Nar | 2200e2000BC | Human tooth enamel | TA184 | TellAbraq | UAE | LM1 | 0.708839 | *Homo* | Sharjah | TellAbraq |
| Gregoricka 2013 | Bronze age: Umm an-Nar | 2200e2000BC | Human tooth enamel | TA185 | TellAbraq | UAE | LM1 | 0.708876 | *Homo* | Sharjah | TellAbraq |
| Gregoricka 2013 | Bronze age: Umm an-Nar | 2200e2000BC | Human tooth enamel | TA186 | TellAbraq | UAE | LM1 | 0.708866 | *Homo* | Sharjah | TellAbraq |
| Gregoricka 2013 | Bronze age: Umm an-Nar | | Faunal Tooth Enamel | TA98 | TellAbraq | UAE | RM3 | 0.708816 | Goat |  |  |
| Gregoricka 2013 | Bronze age: Umm an-Nar | | Faunal Tooth Enamel | TA99 | TellAbraq | UAE | RM3 | 0.708701 | Goat |  |  |
| Gregoricka 2013 | Bronze age: Umm an-Nar | | Faunal Tooth Enamel | TA100 | TellAbraq | UAE | RM1 | 0.708745 | Sheep |  |  |
| Gregoricka 2013 | Bronze age: Umm an-Nar | | Faunal Tooth Enamel | TA101 | TellAbraq | UAE | RM1 | 0.708843 | Sheep |  |  |
| Gregoricka 2013 | Bronze age: Umm an-Nar | | Faunal Tooth Enamel | TA102 | TellAbraq | UAE | LM3 | 0.708746 | Sheep |  |  |
| Gregoricka 2013 | Bronze age: Umm an-Nar | | Faunal Tooth Enamel | TA103 | TellAbraq | UAE | RM1 | 0.708668 | Sheep |  |  |
| Gregoricka 2013 | Bronze age: Umm an-Nar | | Faunal Tooth Enamel | TA104 | TellAbraq | UAE | LM3 | 0.708760 | Sheep |  |  |
| Gregoricka 2013 | Bronze age: Umm an-Nar | | Faunal Tooth Enamel | TA105 | TellAbraq | UAE | RM1/2 | 0.708756 | Cattle |  |  |
| Gregoricka 2013 | Bronze age: Umm an-Nar | | Faunal Tooth Enamel | TA106 | TellAbraq | UAE | RM2 | 0.708788 | Cattle |  |  |
| Gregoricka 2013 | Bronze age: Umm an-Nar | | Faunal Tooth Enamel | TA107 | TellAbraq | UAE | LM2 | 0.708797 | Cattle |  |  |
| Gregoricka 2013 | Bronze age: Umm an-Nar | | Faunal Tooth Enamel | TA108 | TellAbraq | UAE | RM2/3 | 0.708826 | Cattle |  |  |
| Gregoricka 2013 | Bronze age: Umm an-Nar | | Faunal Tooth Enamel | TA109 | TellAbraq | UAE | RM1 | 0.708862 | Cattle |  |  |
| Gregoricka et al 2014 | Bronze age |  | Human tooth enamel | TA186/187 | TellAbraq | UAE | M1 | 0.70887 | *Homo* |  |  |
| Gregoricka et al 2014 | Bronze age |  | Human tooth enamel | TA186/187 | TellAbraq | UAE | M2 | 0.70887 | *Homo* |  |  |
| Gregoricka 2013 | Bronze age: Umm an-Nar | 2700e2500BC | Human tooth enamel | UaN143 | Umm_an-Nar_Island | UAE | LM1 | 0.709035 | *Homo* | Abu_Dhabi | TombV |
| Gregoricka 2013 | Bronze age: Umm an-Nar | 2700e2500BC | Human tooth enamel | UaN144 | Umm_an-Nar_Island | UAE | LM1 | 0.708918 | *Homo* | Abu_Dhabi | TombV |
| Gregoricka 2013 | Bronze age: Umm an-Nar | 2700e2500BC | Human tooth enamel | UaN146 | Umm_an-Nar_Island | UAE | LM1 | 0.708871 | *Homo* | Abu_Dhabi | TombV |
| Gregoricka 2013 | Bronze age: Umm an-Nar | 2700e2500BC | Human tooth enamel | UaN147 | Umm_an-Nar_Island | UAE | LM1 | 0.708958 | *Homo* | Abu_Dhabi | TombV |
| Gregoricka 2013 | Bronze age: Umm an-Nar | 2700e2500BC | Human tooth enamel | UaN148 | Umm_an-Nar_Island | UAE | LM1 | 0.708959 | *Homo* | Abu_Dhabi | TombV |
| Gregoricka 2013 | Bronze age: Umm an-Nar | 2700e2500BC | Human tooth enamel | UaN149 | Umm_an-Nar_Island | UAE | LM1 | 0.708939 | *Homo* | Abu_Dhabi | TombV |
| Gregoricka 2013 | Bronze age: Umm an-Nar | 2700e2500BC | Human tooth enamel | UaN150 | Umm_an-Nar_Island | UAE | LM1 | 0.709029 | *Homo* | Abu_Dhabi | TombV |
| Gregoricka 2013 | Bronze age: Umm an-Nar | 2700e2500BC | Human tooth enamel | UaN151 | Umm_an-Nar_Island | UAE | LM1 | 0.708933 | *Homo* | Abu_Dhabi | TombV |
| Gregoricka 2013 | Bronze age: Umm an-Nar | 2700e2500BC | Human tooth enamel | UaN152 | Umm_an-Nar_Island | UAE | LM1 | 0.708971 | *Homo* | Abu_Dhabi | TombV |
| Gregoricka 2013 | Bronze age: Umm an-Nar | 2700e2500BC | Human tooth enamel | UaN153 | Umm_an-Nar_Island | UAE | LM1 | 0.708903 | *Homo* | Abu_Dhabi | TombV |
| Gregoricka 2013 | Bronze age: Umm an-Nar | 2700e2500BC | Human tooth enamel | UaN154 | Umm_an-Nar_Island | UAE | LM1 | 0.708903 | *Homo* | Abu_Dhabi | TombV |
| Gregoricka 2013 | Bronze age: Umm an-Nar | 2700e2500BC | Human tooth enamel | UaN155 | Umm_an-Nar_Island | UAE | LM1 | 0.708881 | *Homo* | Abu_Dhabi | TombV |
| Gregoricka 2013 | Bronze age: Umm an-Nar | 2700e2500BC | Human tooth enamel | UaN156 | Umm_an-Nar_Island | UAE | LM1 | 0.708947 | *Homo* | Abu_Dhabi | TombV |
| Gregoricka 2013 | Bronze age: Umm an-Nar | 2700e2500BC | Human tooth enamel | UaN157 | Umm_an-Nar_Island | UAE | LM1 | 0.708908 | *Homo* | Abu_Dhabi | TombV |
| Gregoricka 2013 | Bronze age: Umm an-Nar | 2500e2300BC | Human tooth enamel | UaN120 | Umm_an-Nar_Island | UAE | RM1 | 0.708795 | *Homo* | Abu_Dhabi | Tomb_I |
| Gregoricka 2013 | Bronze age: Umm an-Nar | 2500e2300BC | Human tooth enamel | UaN121 | Umm_an-Nar_Island | UAE | RM1 | 0.708872 | *Homo* | Abu_Dhabi | Tomb_I |
| Gregoricka 2013 | Bronze age: Umm an-Nar | 2500e2300BC | Human tooth enamel | UaN122 | Umm_an-Nar_Island | UAE | RM1 | 0.709001 | *Homo* | Abu_Dhabi | Tomb_I |
| Gregoricka 2013 | Bronze age: Umm an-Nar | 2500e2300BC | Human tooth enamel | UaN123 | Umm_an-Nar_Island | UAE | RM1 | 0.708905 | *Homo* | Abu_Dhabi | Tomb_I |
| Gregoricka 2013 | Bronze age: Umm an-Nar | 2500e2300BC | Human tooth enamel | UaN125 | Umm_an-Nar_Island | UAE | LM1 | 0.708896 | *Homo* | Abu_Dhabi | Tomb_II |
| Gregoricka 2013 | Bronze age: Umm an-Nar | 2500e2300BC | Human tooth enamel | UaN127 | Umm_an-Nar_Island | UAE | LM1 | 0.708916 | *Homo* | Abu_Dhabi | Tomb_II |
| Gregoricka 2013 | Bronze age: Umm an-Nar | 2500e2300BC | Human tooth enamel | UaN128 | Umm_an-Nar_Island | UAE | LM1 | 0.708906 | *Homo* | Abu_Dhabi | Tomb_II |
| Gregoricka 2013 | Bronze age: Umm an-Nar | 2500e2300BC | Human tooth enamel | UaN130 | Umm_an-Nar_Island | UAE | LM1 | 0.708649 | *Homo* | Abu_Dhabi | Tomb_II |
| Gregoricka 2013 | Bronze age: Umm an-Nar | 2500e2300BC | Human tooth enamel | UaN132 | Umm_an-Nar_Island | UAE | LM1 | 0.708862 | *Homo* | Abu_Dhabi | Tomb_II |
| Gregoricka 2013 | Bronze age: Umm an-Nar | 2500e2300BC | Human tooth enamel | UaN133 | Umm_an-Nar_Island | UAE | LM1 | 0.708884 | *Homo* | Abu_Dhabi | Tomb_II |
| Gregoricka 2013 | Bronze age: Umm an-Nar | 2500e2300BC | Human tooth enamel | UaN134 | Umm_an-Nar_Island | UAE | LM1 | 0.708942 | *Homo* | Abu_Dhabi | Tomb_II |
| Gregoricka 2013 | Bronze age: Umm an-Nar | 2500e2300BC | Human tooth enamel | UaN135 | Umm_an-Nar_Island | UAE | LM1 | 0.708894 | *Homo* | Abu_Dhabi | Tomb_II |
| Gregoricka 2013 | Bronze age: Umm an-Nar | 2500e2300BC | Human tooth enamel | UaN136 | Umm_an-Nar_Island | UAE | LM1 | 0.708733 | *Homo* | Abu_Dhabi | Tomb_II |
| Gregoricka 2013 | Bronze age: Umm an-Nar | 2500e2300BC | Human tooth enamel | UaN137 | Umm_an-Nar_Island | UAE | LM1 | 0.708765 | *Homo* | Abu_Dhabi | Tomb_II |
| Gregoricka 2013 | Bronze age: Umm an-Nar | 2500e2300BC | Human tooth enamel | UaN138 | Umm_an-Nar_Island | UAE | LM1 | 0.708939 | *Homo* | Abu_Dhabi | Tomb_II |
| Gregoricka 2013 | Bronze age: Umm an-Nar | 2500e2300BC | Human tooth enamel | UaN139 | Umm_an-Nar_Island | UAE | LM1 | 0.708998 | *Homo* | Abu_Dhabi | Tomb_II |
| Gregoricka 2013 | Bronze age: Umm an-Nar | 2500e2300BC | Human tooth enamel | UaN140 | Umm_an-Nar_Island | UAE | LM1 | 0.708897 | *Homo* | Abu_Dhabi | Tomb_II |
| Gregoricka 2013 | Bronze age: Umm an-Nar | 2500e2300BC | Human tooth enamel | UaN141 | Umm_an-Nar_Island | UAE | LM1 | 0.708918 | *Homo* | Abu_Dhabi | Tomb_II |
| Gregoricka 2013 | Bronze age: Umm an-Nar | 2500e2300BC | Human tooth enamel | UaN142 | Umm_an-Nar_Island | UAE | LM1 | 0.708853 | *Homo* | Abu_Dhabi | Tomb_II |
| Gregoricka 2013 | Bronze age: Umm an-Nar | | Faunal Tooth Enamel | UaN36 | Umm_an-Nar_Island | UAE | LM1 | 0.708752 | Cattle |  |  |
| Gregoricka 2013 | Bronze age: Umm an-Nar | | Faunal Tooth Enamel | UaN37 | Umm_an-Nar_Island | UAE | RM3 | 0.708900 | Cattle |  |  |
| Gregoricka 2013 | Bronze age: Umm an-Nar | | Faunal Tooth Enamel | UaN38 | Umm_an-Nar_Island | UAE | RM1 | 0.708826 | Sheep/goat |  |  |
| Gregoricka 2013 | Bronze age: Umm an-Nar | | Faunal Tooth Enamel | UaN39 | Umm_an-Nar_Island | UAE | LM1 | 0.708765 | Sheep/goat |  |  |
| Gregoricka 2013 | Bronze age: Umm an-Nar | | Faunal Tooth Enamel | UaN40 | Umm_an-Nar_Island | UAE | LM2 | 0.708838 | Sheep/goat |  |  |
| Gregoricka 2013 | Bronze age: Umm an-Nar | | Faunal Tooth Enamel | UaN41 | Umm_an-Nar_Island | UAE | RM2 | 0.708892 | Sheep/goat |  |  |
| Gregoricka 2013 | Bronze age: Umm an-Nar | | Faunal Tooth Enamel | UaN43 | Umm_an-Nar_Island | UAE | LM2 | 0.708631 | Sheep/goat |  |  |
| Gregoricka 2013 | Bronze age: Umm an-Nar | | Faunal Tooth Enamel | UaN44 | Umm_an-Nar_Island | UAE | RM1 | 0.708779 | Sheep/goat |  |  |
| Gregoricka 2013 | Bronze age: Umm an-Nar | | Faunal Tooth Enamel | UaN45 | Umm_an-Nar_Island | UAE | LM1 | 0.708695 | Sheep/goat |  |  |
| Gregoricka 2013 | Bronze age: Umm an-Nar | | Faunal Tooth Enamel | UaN46 | Umm_an-Nar_Island | UAE | RM1 | 0.708792 | Sheep/goat |  |  |
| Gregoricka 2013 | Bronze age: Umm an-Nar | | Faunal Tooth Enamel | UaN47 | Umm_an-Nar_Island | UAE | LM2 | 0.708591 | Sheep/goat |  |  |
| Gregoricka 2013 | Bronze age: Umm an-Nar | | Faunal Tooth Enamel | UaN48 | Umm_an-Nar_Island | UAE | RM2 | 0.708867 | Sheep/goat |  |  |
| Gregoricka 2013 | Bronze age: Umm an-Nar | | Faunal Tooth Enamel | UaN49 | Umm_an-Nar_Island | UAE | RM2 | 0.708845 | Sheep/goat |  |  |
| Gregoricka 2013 | Bronze age: Umm an-Nar | | Faunal Tooth Enamel | UaN50 | Umm_an-Nar_Island | UAE | RM2 | 0.708867 | Sheep/goat |  |  |
| Gregoricka 2013 | Bronze age: Umm an-Nar | | Faunal Tooth Enamel | UaN42 | Umm_an-Nar_Island | UAE | RM3 | 0.708775 | Oryx |  |  |
| Gregoricka et al 2014 | Bronze age |  | Human tooth enamel | UaN123/124 | Umm_an-Nar_Island | UAE | M1 | 0.70891 | *Homo* |  |  |
| Gregoricka et al 2014 | Bronze age |  | Human tooth enamel | UaN123/124 | Umm_an-Nar_Island | UAE | M3 | 0.70893 | *Homo* |  |  |
| Gregoricka et al 2014 | Bronze age |  | Human tooth enamel | UaN125/126 | Umm_an-Nar_Island | UAE | M1 | 0.70890 | *Homo* |  |  |
| Gregoricka et al 2014 | Bronze age |  | Human tooth enamel | UaN125/126 | Umm_an-Nar_Island | UAE | M2 | 0.70891 | *Homo* |  |  |
| Gregoricka et al 2014 | Bronze age |  | Human tooth enamel | UaN128/129 | Umm_an-Nar_Island | UAE | M1 | 0.70891 | *Homo* |  |  |
| Gregoricka et al 2014 | Bronze age |  | Human tooth enamel | UaN128/129 | Umm_an-Nar_Island | UAE | M3 | 0.70900 | *Homo* |  |  |
| Gregoricka et al 2014 | Bronze age |  | Human tooth enamel | UaN130/131 | Umm_an-Nar_Island | UAE | M1 | 0.70865 | *Homo* |  |  |
| Gregoricka et al 2014 | Bronze age |  | Human tooth enamel | UaN130/131 | Umm_an-Nar_Island | UAE | M3 | 0.70873 | *Homo* |  |  |
| Gregoricka et al 2014 | Bronze age |  | Human tooth enamel | UaN144/145 | Umm_an-Nar_Island | UAE | M1 | 0.70892 | *Homo* |  |  |
| Gregoricka et al 2014 | Bronze age |  | Human tooth enamel | UaN144/145 | Umm_an-Nar_Island | UAE | M3 | 0.70893 | *Homo* |  |  |
|  |  |  |  |  |  |  |  |  |  |  |  |
| Kutterer et al 2017 | Modern |  | Modern wood | 1 | SE Arabia |  |  | 0.70917 |  |  |  |
| Kutterer et al 2017 | Modern |  | Modern wood | 2 | SE Arabia |  |  | 0.70916 |  |  |  |
| Kutterer et al 2017 | Modern |  | Modern wood | 3 | SE Arabia |  |  | 0.70895 |  |  |  |
| Kutterer et al 2017 | Modern |  | Modern wood | 4 | SE Arabia |  |  | 0.70890 |  |  |  |
| Kutterer et al 2017 | Modern |  | Modern wood | 5 | SE Arabia |  |  | 0.70879 |  |  |  |
| Kutterer et al 2017 | Modern |  | Modern wood | 6 | SE Arabia |  |  | 0.70874 |  |  |  |
| Kutterer et al 2017 | Modern |  | Modern wood | 7 | SE Arabia |  |  | 0.70871 |  |  |  |
| Kutterer et al 2017 | Modern |  | Modern wood | 8 | SE Arabia |  |  | 0.70871 |  |  |  |
| Kutterer et al 2017 | Modern |  | Modern wood | 9 | SE Arabia |  |  | 0.70869 |  |  |  |
| Kutterer et al 2017 | Modern |  | Modern wood | 10 | SE Arabia |  |  | 0.70868 |  |  |  |
| Kutterer et al 2017 | Modern |  | Modern wood | 11 | SE Arabia |  |  | 0.70867 |  |  |  |
| Kutterer et al 2017 | Modern |  | Modern wood | 12 | SE Arabia |  |  | 0.70867 |  |  |  |
| Kutterer et al 2017 | Modern |  | Modern wood | 13 | SE Arabia |  |  | 0.70865 |  |  |  |
| Kutterer et al 2017 | Modern |  | Modern wood | 14 | SE Arabia |  |  | 0.70863 |  |  |  |
| Kutterer et al 2017 | Modern |  | Modern wood | 15 | SE Arabia |  |  | 0.70863 |  |  |  |
| Kutterer et al 2017 | Modern |  | Modern wood | 16 | SE Arabia |  |  | 0.70863 |  |  |  |
| Kutterer et al 2017 | Modern |  | Modern wood | 17 | SE Arabia |  |  | 0.70861 |  |  |  |
| Kutterer et al 2017 | Modern |  | Modern wood | 18 | SE Arabia |  |  | 0.70860 |  |  |  |
| Kutterer et al 2017 | Modern |  | Modern wood | 19 | SE Arabia |  |  | 0.70855 |  |  |  |
| Kutterer et al 2017 | Modern |  | Modern wood | 20 | SE Arabia |  |  | 0.70854 |  |  |  |
| Kutterer et al 2017 | Modern |  | Modern wood | 21 | SE Arabia |  |  | 0.70854 |  |  |  |
| Kutterer et al 2017 | Modern |  | Modern wood | 22 | SE Arabia |  |  | 0.70845 |  |  |  |
| Kutterer et al 2017 | Modern |  | Modern wood | 23 | SE Arabia |  |  | 0.70845 |  |  |  |
| Kutterer et al 2017 | Modern |  | Modern wood | 24 | SE Arabia |  |  | 0.70843 |  |  |  |
| Kutterer et al 2017 | Modern |  | Modern wood | 25 | SE Arabia |  |  | 0.70852 |  |  |  |
| Kutterer et al 2017 | Modern |  | Modern wood | 26 | SE Arabia |  |  | 0.70914 |  |  |  |
| Kutterer et al 2017 | Modern |  | Modern wood | 27 | SE Arabia |  |  | 0.70850 |  |  |  |
| Kutterer et al 2017 | Modern |  | Modern wood | 28 | SE Arabia |  |  | 0.70849 |  |  |  |
|  |  |  |  |  |  |  |  |  |  |  |  |
| Current study | Late Pre-Islamic | | Modern Plant ML |  | Mleiha | UAE |  | 0.708602 |  |  |  |
| Current study | Late Pre-Islamic | | Textile |  | Mleiha | UAE |  | 0.708836 |  |  |  |
| Current study | Late Pre-Islamic | | Textile |  | Mleiha | UAE |  | 0.711216 |  |  |  |
| Current study | Late Pre-Islamic | | Textile |  | Mleiha | UAE |  | 0.710391 |  |  |  |
| Current study | Late Pre-Islamic | | Textile |  | Mleiha | UAE |  | 0.714131 |  |  |  |
| Current study | Late Pre-Islamic | | Textile |  | Mleiha | UAE |  | 0.709800 |  |  |  |
| Current study | Late Pre-Islamic | | Textile |  | Mleiha | UAE |  | 0.708978 |  |  |  |
| Current study | Late Pre-Islamic | | Textile |  | Mleiha | UAE |  | 0.709343 |  |  |  |
| Current study | Late Pre-Islamic | | Arch Cotton Seed |  | Mleiha | UAE |  | 0.710422 |  |  |  |
| Current study | Late Pre-Islamic | | Arch Cotton Seed |  | Mleiha | UAE |  | 0.712766 |  |  |  |
| Current study | Late Pre-Islamic | | Arch Cotton Seed |  | Mleiha | UAE |  | 0.709696 |  |  |  |
| Current study | Late Pre-Islamic | | Arch non cot seed |  | Mleiha | UAE |  | 0.708738 |  |  |  |
| Current study | Late Pre-Islamic | | Arch non cot seed |  | Mleiha | UAE |  | 0.708695 |  |  |  |

**Table S6**. Strontium isotope data for all comparative sites. * Some of the animals used in these studies may have been imported, as could some of the humans have been migrants, but the relatively narrow range of values, with low within-site variability, nevertheless provides a useful estimate of the strontium isotope ratios over the wide geographical extent.

**SI References**

1. Dalongeville, R. Mleiha: étude physique et paléoenvironment. , Lyon, pp. . in *Mleiha I; Environment, Statégies de Subsistance et Artisanats* (ed. Mouton, M.) 33–54 (Travaux Maison de l’Orient 29, 1999).

2. Mouton, M. & Schiettecatte, J. In the desert margins: the settlement process in ancient South and East Arabia. *Rome ‘L’Erma’ Di Bretschneider* (2014).

3. Mouton, M. Mleiha et le peuplement de la péninsule d’Oman a la période Pré-Islamique Récente. *L’Erma di Bretschneider* (2010).

4. Cuny, J. & Mouton, M. La transition vers la période sassanide dans la péninsule d’Oman: chronologie et modes de peuplement. in *L’Arabie à la veille de l’Islam: Bilan Clinique* (eds. Schiettecatte, J. & Robin, C.) 91–133 (De Boccard, Paris, 2008).

5. Benoist, A., Mouton, M. & Schiettecatte, J. The artefacts from the fort at Mleiha: distribution, origins, trade and dating. *Proc. Semin. Arab. Stud.* (2003).

6. Mouton, M. *et al.* Building H at Mleiha: new evidence of the late pre-Islamic period D phase (PIR.D) in the Oman peninsula (second to mid-third century AD). in *Proceedings of the Seminar for Arabian Studies 42* 205–222 (2012).

7. Dabrowski, V. Supplying strategies and plant resources management in eastern Arabia during Classical and Islamic times (IVth c. BC – XVIth c. AD) : archaeobotanical and archaeoentomological approches. (Muséum national d’histoire naturelle, 2019).

8. Goudie, A. S., Parker, A. G. & Al-Farraj, A. Coastal change in Ras Al Khaimah (United Arab Emirates): A Cartographic Analysis. *Geogr. J.* (2000). doi:10.1111/j.1475-4959.2000.tb00003.x

9. Ministry of energy UAE. *Geological map of the Northern Emirates, 1:250,000 series Sheet 250-1.* (2006).

10. Al Farraj, A. & Harvey, A. M. Late Quaternary interactions between aeolian and fluvial processes: A case study in the northern UAE. *J. Arid Environ.* (2004). doi:10.1016/S0140-1963(03)00054-5

11. Parker, A. G. & Goudie, A. S. Geomorphological and palaeoenvironmental investigations in the southeastern Arabian Gulf region and the implication for the archaeology of the region. *Geomorphology* (2008). doi:10.1016/j.geomorph.2007.04.028

12. Bulliet, R. W. *Cotton, Climate, and Camels in Early Islamic Iran*. (Columbia University Press, 2009).

13. Théophraste. *Recherches sur les plantes: à l’origine de la botanique*. (Belin, 2010).

14. Hojlund, F. & Andersen, H. H. *Qala’at al-Bahrain vol. 1. The northern city wall and the islamic fortress*. (Aarhus University Press, 1994).

15. Rahman, M., Castillo, C. C., Murphy, C., Rahman, S. M. & Fuller, D. Q. Agricultural systems in Bangladesh: the first archaeobotanical results from Early Historic Wari-Bateshwar and Early Medieval Vikrampura. *Archaeol. Anthropol. Sci.* (2020). doi:10.1007/s12520-019-00991-5

16. Lamm, C. J. *Cotton in Mediaeval Textiles of the Near East*. (Paris, P. Geuthner, 1937).

17. Mazzaoui, M. F. *The Italian cotton industry in the later Middle Ages, 1100-1600*. (Cambridge University Press, 1981).

18. Chen, T. *et al.* Archaeobotanical Study of Ancient Food and Cereal Remains at the Astana Cemeteries, Xinjiang, China. in *PLoS ONE* **7**, (2012).

19. Bouchaud, C., Clapham, A. & Newton, C. Cottoning on to cotton (Gossypium sp.) in Arabia and Africa during Antiquity. in *Plants and People in the African Past Plants* (Springer, 2018).

20. Yvanez, E. & Wozniak, M. Cotton in ancient Sudan and Nubia: Archaeological sources and historical implications. *Rev. d’ethnoécologie* (2019). doi:10.4000/ethnoecologie.4429

21. Griffith, F. L. & Crowfoot, G. M. On the Early Use of Cotton in the Nile Valley. *J. Egypt. Archaeol.* **20**, 5–12 (1934).

22. Mayer-Thurman, C. C. & Williams, B. *Ancient textiles from Nubia: Meroitic, X-group, and christian fabrics from Ballana and Qustul*. (Art Institute of Chicago, 1979).

23. E. Strouhal. *Wadi Qitna and Kalabsha South I: Archaeology*. (Czech Institute of Egyptology, 1984).

24. Thanheiser, U. Plant remains from Kellis: First results. in *Dakhleh Oasis Project: Preliminary Reports on the 1992-1993 and 1993-1994 Field Seasons* (eds. Hope, C. & Mills, A.) 89–93 (Oxbow Books, Oxford, 1999).

25. Thanheiser, U. Roman agriculture and gardening in Egypt as seen from Kellis. in *Dakhleh Oasis Project: Preliminary Reports on the 1994-1995 to 1998-1999 Field Seasons* (eds. Hope, C. A. & Bowen, G. E.) 299–310 (Oxbow Books, Oxford, 2002).

26. Livingstone, R. J. Dress and identity in Kellis, a Roman-Period village in Egypt. (Monash University, 2015).

27. Bagnall, R. S. *The Kellis agricultural account book*. (Oxbow Books, 1997).

28. Thanheiser, U. & Walter, J. Plant use in a Romano-Egyptian household in the third century CE. in *Amheida II: A Late Romano-Egyptian House in the Dakhleh Oasis. Amheida House B2* (ed. Boozer, A. L.) 375–392 (The Institute for the study of the Ancient world, 2015).

29. Boozer, A. L. Woven material. in *Amheida II: A Late Romano-Egyptian House in the Dakhleh Oasis/Amheida House B2* (ed. Boozer, A. L.) (The Institute for the study of the Ancient world, 2015).

30. Bagnall, R. S. Cotton, and the Economy of the Small Oasis. *Bull. Am. Soc. Papyrol.* **45**, 21–30 (2008).

31. Bagnall, R. S. & Cribiore, R. *Women’s letters from Ancient Egypt, 300 BC-AD 800*. (University of Michigan Press, 2006).

32. Wild, J. P. Cotton in Roman Egypt: Some problems of origin. *Al-Rafidan* **18**, 287–98 (1997).

33. Wild, F. C. & Wild, J. P. Sails from the Roman port at Berenike, Egypt. *Int. J. Naut. Archaeol.* **30**, 211–220 (2001).

34. Wild, J. P. & Wild, F. C. Berenike and textile trade on the Indian ocean. in *Textile Trade and Distribution in Antiquity* (ed. Droß-Krüpe, K.) 91–110 (Harrassowitz, 2014).

35. Wild, J. P. & Wild, F. C. Qasr Ibrim: New perspectives on the changing textile cultures of Lower Nubia. in *Egypt in the First Millenium A.D., British Museum Publications on Egypt and Sudan* (ed. O’Connell, E. R.) 71–80 (Peeters Publishers, 2014).

36. Eastwood, G. Textiles. in *Quseir Al-Qadim 1980 : Preliminary Report* (eds. Whitcomb, D. & Johnson, J.) 285–326 (Undena Publications, 1982).

37. Whitewright, J. Roman rigging material from the red sea port of myos hormos. *Int. J. Naut. Archaeol.* (2007). doi:10.1111/j.1095-9270.2007.00150.x

38. Handley, F. J. L. Quseir al-Qadim 2003: The textiles. *Text. Newsl.* **38**, 27–30 (2004).

39. Gallazzi, C. Umm-el-Breigât (Tebtynis) : Campagna di scavo dell’anno 2012. *RIL* **146**, 87–110 (2012).

40. Kvavadze, E. & Gagoshidze, I. Fibres of silk, cotton and flax in a weaving workshop from the first century a.d. palace of Dedoplis Gora, Georgia. in *Vegetation History and Archaeobotany* (2008). doi:10.1007/s00334-008-0175-5

41. Reddy, A., Attaelmanan, A. G. & Mouton, M. Pots, plates and provenance: Sourcing Indian coarse wares from Mleiha using X-ray fluorescence (XRF) spectrometry analysis. in *IOP Conference Series: Materials Science and Engineering* (2012). doi:10.1088/1757-899X/37/1/012010

42. Casson, L. *The Periplus Maris Erythraei. Text with Introduction, Translation, and Commentary*. (Princeton, NJ: Princeton Univeristy Press, 1989).

43. Janaway, R. C. & Coningham, R. A. E. A review of archaeological textile evidence from South Asia. *South Asian Stud.* (1995). doi:10.1080/02666030.1995.9628502

44. Wild, J.-P. & Wild, F. C. Through Roman eyes: cotton textiles from Early Historic India. in *A Stitch in Time. Essays in Honour of Lise Bender Jørgensen, Gothenburg Archaeological studies* (eds. Bergerbrant, S. & Fossøy, S. H.) 209–235 (Gothenburg University, 2014).

45. Seland, E. H. Here, There and Everywhere. A Network Approach to Textile Trade in the Periplus Maris Erythraei. in *Textiles, Trade and Theories. From the Ancient Near East to the Mediterranean* (eds. Droß-Krüpe, K. & Nosch, M.-L.) 211–219 (Ugarit-Verlag, 2016).

46. D. Schlingloff. Cotton-manufacture in ancient India. *J. Econ. Soc. Hist. Orient/Journal l’histoire Econ. Soc. l’Orient* **17**, 81–90 (1974).

47. Fuller, D. Q. The spread of textile production and textile crops in India beyond the Harappan zone: an aspect of the emergence of craft specialization and systematic trade. *Linguist. Archaeol. Hum. past. Indus Proj. Kyoto Res. Inst. Humanit. Nat.* 1–26 (2008).

48. Pokharia, A. K. & Saraswat, K. S. Plant economy during Kushana period (100-300 AD) at ancient Sanghol. *Pragdhara* **9**, 75–122 (1999).

49. Cooke, M., Fuller, D. Q. & Rajan, K. Early Historic Agriculture in Southern Tamil Nadu: Archaeobotanical Research at Mangudi, Kodumanal and Perur. in *South Asian Archaeology* (eds. Linden, S., Weisshaar, J. & Franke-Vogt, U.) 329–334 (2005).

50. Pokharia, A. K. Plant macroremains from Sarethi: An Early Historic site in Saryu region of Ganga Plain, Uttar Pradesh. *Palaeobot.* **68**, 125–137 (2019).

51. Fuller, D. Archaeobotany. in *Transformations in Early Historic and Early Medieval India: Excavations at Paithan, Maharashtra 1996-1999* (eds. Kennet, D., Bai, K. M. & Rao, V. J.) 279–388 (Archaeological survey of India, British Association for South Asian Studies, 2019).

52. Connan, J. & Mouton, M. Study of some bituminous lumps of Mleiha : Preliminary results. in *Mleiha I: Environnement, Stratégies de Subsistance et Artisanats, Travaux de la Maison de l’Orient méditerranéen* (ed. Mouton, M.) 245–264 (De Boccard, 1999).

53. Quillien, L. Dissemination and price of cotton in Mesopotamia during the 1st millennium BCE. *Rev. d’ethnoécologie* (2019). doi:10.4000/ethnoecologie.4239

54. Rahmani, L. Y. Jason’s tomb. *Isr. Explor. Soc.* **17**, 61–100 (1967).

55. Shamir, O. Cotton textiles from the Byzantine period to the Medieval period in ancient Palestine. *Rev. d’ethnoécologie* (2019). doi:10.4000/ethnoecologie.4176

56. Müller, M. Identification of the textiles from Khirbet Qumran using microscopy and synchrotron radiation x-ray fibre diffraction. in *Khirbet Qumrân and ’Aïn Feshkha II. Studies of Anthropology, Physics and Chemistry* (eds. Humbert, J.-B. & Gunneweg, J.) 277–286 (Academic Press, Vandenhoeck & Ruprecht, 2003).

57. Granger-Taylor, H. The textiles from Khirbet Qazone (Jordan). in *Archéologie Des Textiles, Des Origines Au Ve Siècle: Actes Du Colloque de Lattes, Octobre 1999* (eds. Cardon, D. & Feugère, M.) 149–161 (Monique Mergoil, 2000).

58. Ramsay, J. H. & Parker, S. T. A diachronic look at the agricultural economy at the Red Sea Port of Aila: An archaeobotanical case for hinterland production in arid environments. *Bulletin of the American Schools of Oriental Research* (2016). doi:10.5615/bullamerschoorie.376.0101

59. Pelling, R. Garamantian agriculture and its significance in a wider North African context: The evidence of the plant remains from the Fazzan project. *J. North African Stud.* (2005). doi:10.1080/13629380500336763

60. Pelling, R. Garamantian agriculture: the plant remains from Jarma, Fazzan. *Libyan Stud.* (2008). doi:10.1017/s0263718900009997

61. Pelling, R. The archaeobotanical remains (Chapter 18) and Botanical data appendices (Chapter 28). in *The Archaeology of Fazzan, Vol. 4, Survey and Excavations at Old Jarma (Ancient Garama) Carried out by C.M. Daniels (1961-1969) and the Fazzan Project (1997-2001)* (ed. Mattingly, D. J.) 473–94, 841–52 (Society of Lybian Studies, Department of Antiquities, 2013).

62. Bouchaud, C., Tengberg, M. & Prà, P. D. Cotton cultivation and textile production in the Arabian Peninsula during antiquity; the evidence from Madâ’in Sâlih (Saudi Arabia) and Qal’at al-Bahrain (Bahrain). *Veg. Hist. Archaeobot.* (2011). doi:10.1007/s00334-011-0296-0

63. Murphy, C. *et al.* Early agriculture in Sri Lanka: New Archaeobotanical analyses and radiocarbon dates from the early historic sites of Kirinda and Kantharodai (Kandarodai). *Archaeol. Res. Asia* (2018). doi:10.1016/j.ara.2018.06.001

64. Fuller, D. Q. Agriculture innovation and state collapse in Meroitic Nubia. in *Archaeology of African Plant Use* (eds. Stevens, C. J., Nixon, S., Murray, M. A. & Fuller, D. Q.) 165–177 (Left Coast Press, 2014).

65. Massey, R. E. A note on the early history of cotton. *Sudan Notes Rec.* **6**, (1923).

66. Dunham, D. *The Royal cemeteries of Kush, volume V: The west and south cemeteries at Meroë*. (Museum of Fine Arts, 1963).

67. E. Littmann. *Deutsche Aksum-Expedition. Bd IV. Sabaische, griechische und altabessinische Inschriften*. (Museum of Berlin, 1913).

68. Yvanez, E. Les textiles des nécropoles méroïtiques de Saï. *CRIPEL* **29**, 331–344 (2012).

69. Vila, A. *Aksha II. Le cimetière méroïtique d’Aksha*. (Librairie Klincksieck, 1967).

70. Zabkar, L. V. & Zabkar, J. J. Semna South. A Preliminary Report on the 1966-68 Excavations of the University of Chicago Oriental Institute Expedition to Sudanese. *J. Am. Res. Cent. Egypt* **19**, 7–50 (1982).

71. Pfister, R. & Bellinger, L. *The Excavations at Dura-Europos. Final report IV. Part II, The Textiles, New Haven*. (Yale University Press, 1945).

72. A. Schmidt-Colinet. *Palmyra: Kulturbegegnung im Grenzbereich*. (Verlag Philipp von Zabern, 1995).

73. A. Stauffer. The Textiles from Palmyra: technical analyses and their evidence for archaeological research. in *Archéologie Des Textiles, Des Origines Au Ve Siècle: Actes Du Colloque de Lattes, Octobre 1999* (eds. Cardon, D. & Feugère, M.) 247–252 (Monique Mergoil, 2000).

74. Castillo, C. C., Bellina, B. & Fuller, D. Q. Rice, beans and trade crops on the early maritime Silk Route in Southeast Asia. *Antiquity* (2016). doi:10.15184/aqy.2016.175

75. Cameron, J. The archaeological textiles from ban don ta phet in broader perspectiven. in *50 Years of Archaeology in Southeast Asia: Essays in Honour of Ian Glover* (eds. Bellina-Pryce, B., Pryce, T. O., Bacus, E. & Wisseman-Christie, J.) 141–151 (River Books, 2010).

76. Glover, I. C. & Bellina, B. Ban Don Ta Phet and Khao Sam Kaeo: the earliest Indian contact reassessed. in *Early Interactions between South and Southeast Asia: Reflections on Cross-Cultural Movements* (eds. Manguin, P.-Y., Mani, A. & Wade, G.) 17–45 (Institute of Southeast Asian Studies, 2011).

77. Trombert, E. Une trajectoire d’Ouest en Est sur la Route de la soie. La diffusion du coton dans l’Asie Centrale sinisée.(6e-10e siècles). in *La Persia e l’Asia Centrale Da Alessandro al X Secolo (Roma, 9-12 Novembre 1994)* 205–227 (Instituto italiano per il medio ed estremo oriente, 1996).

78. Lagad, R. A., Singh, S. K. & Rai, V. K. Rare earth elements and 87Sr/86Sr isotopic characterization of Indian Basmati rice as potential tool for its geographical authenticity. *Food Chem.* **217**, 254–265 (2017).

79. Valentine, B. T. Immigrant identity in the Indus Civilization: A multi-site isotopic mortuary analysis. (UNIVERSITY OF FLORIDA, 2013).

80. Valentine, B. *et al.* Evidence for patterns of selective urban migration in the greater Indus Valley (2600-1900 BC): A lead and strontium isotope mortuary analysis. *PLoS One* (2015). doi:10.1371/journal.pone.0123103

81. Chase, B., Meiggs, D., Ajithprasad, P. & Slater, P. A. What is left behind: Advancing interpretation of pastoral land-use in Harappan Gujarat using herbivore dung to examine biosphere strontium isotope (87Sr/86Sr) variation. *J. Archaeol. Sci.* (2018). doi:10.1016/j.jas.2018.01.007

82. Chakraborty, K. S. *et al.* Enamel isotopic data from the domesticated animals at Kotada Bhadli, Gujarat, reveals specialized animal husbandry during the Indus Civilization. *J. Archaeol. Sci. Reports* (2018). doi:10.1016/j.jasrep.2018.06.031

83. Kenoyer, J. M., Price, T. D. & Burton, J. H. A new approach to tracking connections between the Indus Valley and Mesopotamia: Initial results of strontium isotope analyses from Harappa and Ur. *J. Archaeol. Sci.* (2013). doi:10.1016/j.jas.2012.12.040

84. Gregoricka, L. A. Residential mobility and social identity in the periphery: Strontium isotope analysis of archaeological tooth enamel from southeastern Arabia. *J. Archaeol. Sci.* **40**, 452–464 (2013).

85. Gregoricka, L. A. Assessing life history from commingled assemblages: The biogeochemistry of inter-tooth variability in Bronze Age Arabia. *J. Archaeol. Sci.* **47**, 10–21 (2014).

86. Kutterer, A., Jasim, S. A. & Yousif, E. Buried far from home: Sasanian graves at Jebel al-Emeilah (Sharjah, UAE). *Arab. Archaeol. Epigr.* **26**, 43–54 (2015).

87. Kutterer, A. & Uerpmann, H. P. Neolithic nomadism in south-east Arabia — strontium and oxygen isotope ratios in human tooth enamel from al-Buhais 18 and Umm al-Quwain 2 in the Emirates of Sharjah and Umm al-Quwain (UAE). *Arab. Archaeol. Epigr.* **28**, 75–89 (2017).
